# Genotoxic Effect of *Salmonella* Paratyphi A Infection on Human Primary Gallbladder Cells

**DOI:** 10.1128/mBio.01911-20

**Published:** 2020-09-22

**Authors:** Ludovico P. Sepe, Kimberly Hartl, Amina Iftekhar, Hilmar Berger, Naveen Kumar, Christian Goosmann, Sascha Chopra, Sven Christian Schmidt, Rajendra Kumar Gurumurthy, Thomas F. Meyer, Francesco Boccellato

**Affiliations:** a Department of Molecular Biology, Max Planck Institute for Infection Biology, Berlin, Germany; b Technische Universität Berlin, Institute of Biotechnology, Berlin, Germany; c Medical Department, Division of Gastroenterology and Hepatology, Charité University Medicine, Berlin, Germany; d Department of Surgery, Campus Charité Mitte and Campus Virchow Clinics, Charité University Medicine, Berlin, Germany; e Department of General and Visceral Surgery, Ernst von Bergmann Clinics, Potsdam, Germany; f Ludwig Institute for Cancer Research, Nuffield Department of Medicine, University of Oxford, Oxford, United Kingdom; University of Pittsburgh School of Medecine

**Keywords:** DNA damage, gallbladder, mucosoid cultures, organoid cultures, *Salmonella*, typhoid toxin, gallbladder cancer

## Abstract

Carcinoma of the gallbladder (GBC) is the most frequent tumor of the biliary tract. Despite epidemiological studies showing a correlation between chronic infection with Salmonella enterica Typhi/Paratyphi A and GBC, the underlying molecular mechanisms of this fatal connection are still uncertain. The murine serovar *Salmonella* Typhimurium has been shown to promote transformation of genetically predisposed cells by driving mitogenic signaling. However, insights from this strain remain limited as it lacks the typhoid toxin produced by the human serovars Typhi and Paratyphi A. In particular, the CdtB subunit of the typhoid toxin directly induces DNA breaks in host cells, likely promoting transformation. To assess the underlying principles of transformation, we used gallbladder organoids as an infection model for *Salmonella* Paratyphi A. In this model, bacteria can invade epithelial cells, and we observed host cell DNA damage. The induction of DNA double-strand breaks after infection depended on the typhoid toxin CdtB subunit and extended to neighboring, non-infected cells. By cultivating the organoid derived cells into polarized monolayers in air-liquid interphase, we could extend the duration of the infection, and we observed an initial arrest of the cell cycle that does not depend on the typhoid toxin. Non-infected intoxicated cells instead continued to proliferate despite the DNA damage. Our study highlights the importance of the typhoid toxin in causing genomic instability and corroborates the epidemiological link between *Salmonella* infection and GBC.

## Abstract (precis)

Bacterial infections are increasingly being recognized as risk factors for the development of adenocarcinomas. The strong epidemiological evidence linking Helicobacter pylori infection to stomach cancer has paved the way to the demonstration that bacterial infections cause DNA damage in the host cells, initiating transformation. In this regard, the role of bacterial genotoxins has become more relevant. Salmonella enterica serovars Typhi and Paratyphi A have been clinically associated with gallbladder cancer. By harnessing the stem cell potential of cells from healthy human gallbladder explant, we regenerated and propagated the epithelium of this organ *in vitro* and used these cultures to model *S.* Paratyphi A infection. This study demonstrates the importance of the typhoid toxin, encoded only by these specific serovars, in causing genomic instability in healthy gallbladder cells, posing intoxicated cells at risk of malignant transformation.

## INTRODUCTION

Gallbladder cancer (GBC) is an adenocarcinoma with very poor prognosis because early stages are often asymptomatic and few patients can be cured with surgery at initial presentation (1). Although uncommon in Western countries, it has relatively high incidence in the western parts of South America and in the northern part of the Indian subcontinent (2). An intriguing aspect is its putative link to chronic carriage of Salmonella enterica serovar Typhi/Paratyphi A. In these patients, *Salmonella* resides in the gallbladder (GB) both intracellularly and extracellularly by forming biofilms on gallstones (3–5), which serve as a reservoir from where bacteria are intermittently shed into the duodenum (6). A higher incidence of GBC in chronic carriers was first observed after an outbreak of Salmonella enterica in Aberdeen, Scotland (7), an observation confirmed by subsequent epidemiological studies (8, 9).

Epidemiological associations with cancer have also been shown for several other bacterial pathogens. However, studies that illuminate the underlying mechanisms are only just emerging and suggest that infection can lead to genomic instability, which may contribute to the development of cancer (10). Helicobacter pylori, Escherichia coli, and Chlamydia trachomatis have been shown to induce DNA double-strand breaks (DSBs) in host cells (11–15). Evidence suggests that infection with some species not only causes the production of reactive oxygen species (ROS) that induce DNA damage in the host, but can also modify the DNA damage response and thereby induce error-prone mechanisms of repair (10).

Salmonella enterica provokes direct genotoxicity through the action of a crucial effector, the typhoid toxin (16), which is only expressed by the human-specific serovars Typhi (17) and Paratyphi A (18). It has been hypothesized that Salmonella enterica delivers the typhoid toxin through secreted outer membrane vesicles after internalization into the host cell (19, 20). More recently, it has been found that a specific interaction of a subunit of the typhoid toxin (PtlB) with luminal receptors allows the loading of the toxin from the *Salmonella*-containing vacuoles into vesicle carriers (21).

Typhoid toxin is able to induce direct DNA DSBs via its CdtB subunit, a DNase that is translocated into the nucleus of the intoxicated cell (19, 20, 22). CdtB also exists as part of another bacterial toxin: the cytolethal distending toxin (CDT), which is produced by multiple Gram-negative bacterial species, including Helicobacter hepaticus (23). Here, as well, it has been directly linked to tumor development *in vivo* and *in vitro* (24, 25).

Commonly used cell lines in infection biology are mostly derived from cancerous tissues, limiting their utility for studies of early carcinogenic events, since they are already transformed and have alterations in key cellular signaling pathways. Since the epithelium is the prime target of infections and toxins, the development of organoid-based human primary cell models is an invaluable means for illuminating the molecular mechanisms by which bacteria could promote cancer. While organoid or derivative models of human gastrointestinal epithelia from the small intestine (26), colon (27), stomach (28, 29), and intrahepatic duct (30) are available, such a system was developed for murine (31) and human (32) gallbladders only very recently and has not yet been utilized for infection studies (33, 34). A robust *in vitro* model that recapitulates the infection dynamics in healthy human gallbladder epithelium would be of immense value in this regard.

Developing from the foregut, the outer lining of the gallbladder consists of a simple columnar epithelium without any gland or crypt structures. The cells tend to moderately produce mucins (35) and transport bile and organic ions (36–38). They share many similarities with the cholangiocytes of the intrahepatic bile duct (39), and therefore the stem cells of the adult gallbladder might express similar markers, such as CD44, CD13, and LGR5 (40, 41) and also depend on activation of the Wnt/β-catenin pathway for their maintenance (30).

Here, we describe the establishment of human gallbladder organoids and their adaptation into more physiological polarized monolayers. We use these systems to study the human-restricted, GBC-associated Salmonella enterica serovar Paratyphi A and, specifically, the effect of the typhoid toxin on healthy cells. These new models will serve as a useful resource to investigate the interaction of *Salmonella* and its toxin with authentic human tissue.

## RESULTS

### Maintenance of adult gallbladder epithelial stem cells depends on activation of the Wnt/β-catenin pathway.

**FIG 1:**
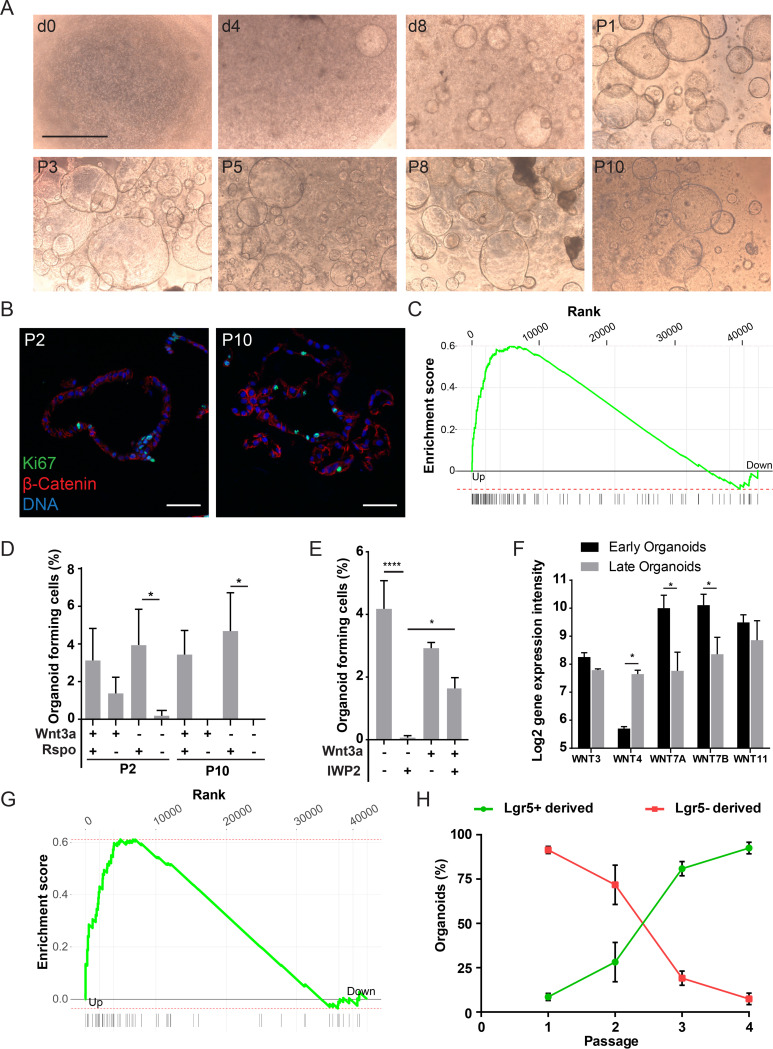
Cultivation of human gallbladder organoids and dependence on the Wnt/β-catenin pathway activation. (A) Gallbladder epithelial cells were isolated and grown as described in Materials and Methods. Pictures were taken 0, 4, and 8 days after seeding and at passages 1, 3, 5, 8, and 10. Scale bar, 1 mm. (B) Gallbladder organoids were fixed 7 days after seeding. Organoids were paraffinized, sectioned, and immunostained for the proliferation marker Ki67 (green), β-catenin (red). DRAQ5 was used to stain the nuclei (blue). (C) Gene set enrichment analysis of human pluripotent stem cell genes published by Mallon et al. (42) among genes regulated in early versus late organoids, as identified by microarray. Adjusted *P* value = 0.00039, enrichment score = 0.6, normalized enrichment score = 1.9. (D) Organoids at passage 1 were split to single cells and seeded, and the number of resulting organoids was counted 5 to 7 days later (i.e., at passage 2), in media + or − Wnt3A and + or − Rspo1. The organoids were kept in culture and the procedure was repeated after 8 passages (i.e., at passage 10). *, *P* < 0.05 (*t* test). (E) Organoids were split to single cells which were seeded in Matrigel and provided with media + or − the Wnt inhibitor IWP-2 and + or − 25% of Wnt3a conditioned medium. The number of resulting organoids was counted 5 to 7 days later. *, *P* < 0.05; ****, *P* < 0.00005. (F) Change in expression levels of Wnt family members observed in a microarray comparing early versus late organoids. Only transcripts with an average log_2_ expression of >6 are shown. *, *P* < 0.05 (*t* test). (G) Gene set enrichment analysis of β-catenin targets published by Herbst et al. (44) among genes regulated in early versus late organoids as identified by microarray. Adjusted *P* value = 0.0015, enrichment score = 0.61, normalized enrichment score = 1.8. (H) Lineage tracing of murine organoids derived from the Lgr5 reporter mouse, Lgr5-EGFP-IRES-CreERT2, ROSA-mTmG^floxed^ after HT induction. The number of organoids derived from Lgr5^+^ cells (green) and Lgr5^–^ cells (red) was counted at each passage 5 to 7 days after seeding. The plot shows the percentage of each population compared to the total number of organoids. Bars indicate the standard deviations (SD).

Primary epithelial cells from human and murine gallbladder (GB) were isolated and grown in Matrigel supplemented with defined medium (Table 1). After 3 to 5 days, the cells started to form hollow spheres, reaching up to 1 mm in diameter (Fig. 1A for humans; see Fig. S1A in the supplemental material for mice). Organoids were passaged every 7 to 10 days by enzymatic and mechanical shearing, and the resulting cells were seeded in fresh Matrigel for further expansion. Cultures expanded indefinitely for murine cells and for human cells. Fluorescence immunohistochemistry for the proliferation markers Ki67 and PCNA showed randomly distributed positive cells at early and late passages (Fig. 1B for humans; see Fig. S1B in the supplemental material for mice). Since only a small fraction of the cells has the ability to form organoids, we assumed that growing organoids accumulate mainly differentiated cells. We therefore analyzed the transcriptome profile of early (4-day-old) versus late (14-day-old) organoids, which confirmed that only the former were enriched in stem cell markers (42) (Fig. 1C and Table 2), indicating their undifferentiated state.

**TABLE 1:** Cultivation medium composition

Reagent^*a*^	Supplier	Catalog no.	Working concentration
Human medium			
Advanced/DMEM/F-12	Invitrogen	12634-010	
R-spondin 1 conditioned medium	In house		25%
B27	Invitrogen	17504-044	1×
N2	Invitrogen	17502-048	1×
Human epidermal growth factor (EGF)	Invitrogen	PHG0311	20 ng/ml
Human noggin	Peprotech	120-10C	150 ng/ml
Human fibroblast growth factor (FGF)-10	Peprotech	100-26	150 ng/ml
Nicotinamide (NIC)	Sigma	N0636	10 mM
A 83-01 (TGF-β type I receptor ALK-5 inhibitor)	Calbiochem	2939	1 μM
Forskolin (FSK)	Tocris	1099	10 μM
Human hepatocyte growth factor (HGF)	Peprotech	100-39	25 ng/ml
Y-27632 (ROCK inhibitor)*	Sigma	Y0503	7.5 μM
Penicillin-streptomycin**	Invitrogen	15140122	1 U/ml
IWP-2***	Merck Millipore	681671	10 μM
Wnt3a conditioned medium***	In house		25%
			
Murine medium			
Advanced/DMEM/F-12	Invitrogen	12634-010	
R-spondin 1 conditioned medium	In house		25%
B27	Invitrogen	17504-044	1×
N2	Invitrogen	17502-048	1×
Murine epidermal growth factor (mEGF)	Invitrogen	PMG8044	50 ng/ml
Murine noggin	Peprotech	250-38	100 ng/ml
Nicotinamide (NIC)	Sigma	N0636	10 mM
A 83-01 (TGF-β type I receptor ALK-5 inhibitor)	Calbiochem	2939	1 μM
Y-27632 (ROCK inhibitor)*	Sigma	Y0503	7.5 μM
Penicillin-streptomycin**	Invitrogen	15140122	1 U/ml

a *, Added only for the first 4 days after seeding; **, not added in infection experiments; ***, only if mentioned.

**TABLE 2:** List of differentially regulated stem cell related genes in early organoids versus late organoids

Probe	Gene symbol	RefSeq	Entrez ID	logFC	Avg expression	*t* score	*P*
A_23_P374844	*GAL*	NM_015973	51083	5.55	9.38	23.45	0.00
A_24_P225616	*RRM2*	NM_001034	6241	4.23	9.90	26.43	0.00
A_24_P397107	*CDC25A*	NM_001789	993	3.39	10.26	23.07	0.00
A_32_P194264	*CHAC2*	NM_001008708	494143	2.75	10.21	9.17	0.01
A_33_P3286208	*LRR1*	NM_203467	122769	2.61	8.83	20.53	0.00
A_33_P3332126	*SCLY*	ENST00000409736	51540	2.45	9.74	17.13	0.00
A_33_P3387856	*CENPN*	NM_001100625	55839	2.42	6.42	6.88	0.01
A_23_P88740	*CENPN*	NM_018455	55839	2.22	12.96	12.74	0.00
A_24_P83678	*MMS22L*	NM_198468	253714	2.14	8.86	8.09	0.01
A_23_P325040	*TMPO*	NM_003276	7112	2.14	8.95	13.02	0.00
A_23_P56553	*METTL8*	NM_024770	79828	2.07	10.98	12.13	0.00
A_33_P3253707	*LRR1*	NM_152329	122769	2.06	12.60	14.00	0.00
A_33_P3379886	*FGF2*	NM_002006	2247	1.99	6.35	3.18	0.07
A_23_P217637	*TIMM8A*	NM_004085	1678	1.94	11.61	11.10	0.00
A_33_P3419696	*FGF2*	NM_002006	2247	1.92	7.69	4.99	0.03
A_24_P49747	*HMGB3P24*	ENST00000433260	NA^*a*^	1.89	6.61	15.70	0.00
A_33_P3489737	*NLN*	NM_020726	57486	1.86	10.59	5.83	0.02
A_24_P244699	*NUDT15*	NM_018283	55270	1.81	9.55	6.06	0.02
A_24_P178093	*TOMM40*	NM_006114	10452	1.77	8.62	10.80	0.00
A_32_P95914	*MMS22L*	NM_198468	253714	1.75	9.70	6.71	0.01
A_23_P143958	*RPL22L1*	NM_001099645	200916	1.66	16.39	8.91	0.01
A_24_P336853	*PNO1*	NM_020143	56902	1.61	10.31	12.46	0.00
A_33_P3357082	*METTL8*	NM_024770	79828	1.59	9.10	13.84	0.00
A_23_P82823	*PINX1*	NM_017884	54984	1.57	10.41	10.49	0.00
A_23_P209337	*METTL21A*	NM_145280	151194	1.56	10.87	6.70	0.01
A_33_P3250861	*ZIC3*	ENST00000370606	7547	1.29	4.62	1.57	0.24
A_23_P144337	*CCRN4L*	NM_012118	25819	1.29	3.26	6.09	0.02
A_32_P25273	*HSPD1*	NM_002156	3329	1.24	17.11	11.51	0.00
A_23_P150092	*SEPHS1*	NM_012247	22929	1.21	12.66	7.32	0.01
A_21_P0000006	*TOMM40*	NM_001128917	10452	1.21	13.86	9.57	0.01
A_33_P3412613	*TMPO*	NM_001032283	7112	1.18	5.82	6.27	0.02
A_23_P202143	*NOLC1*	NM_004741	9221	1.13	12.02	4.96	0.03
A_33_P3619171	*PMAIP1*	NM_021127	5366	1.09	10.98	4.95	0.03
A_24_P253215	*EMG1*	NM_006331	10436	1.07	12.98	6.16	0.02
A_32_P71788	*FKBP4*	NM_002014	2288	1.06	8.96	9.26	0.01
A_24_P357266	*GRPR*	NM_005314	2925	1.01	4.42	1.66	0.22
A_23_P136504	*SLC25A21*	NM_030631	89874	1.01	5.54	3.14	0.07
A_24_P297888	*MTAP*	NM_002451	4507	1.00	9.97	7.12	0.01
A_33_P3256425	*BICD1*	NM_001714	636	0.94	6.25	2.98	0.08
A_23_P345065	*SCLY*	NM_016510	51540	0.93	10.96	3.47	0.06
A_23_P160881	*SMPDL3B*	NM_001009568	27293	0.93	9.95	2.62	0.10
A_23_P27167	*RNASEH1*	NM_002936	246243	0.93	11.31	5.67	0.02
A_23_P365060	*MDN1*	NM_014611	23195	0.92	6.39	7.63	0.01
A_33_P3388135	*MKKS*	NM_170784	8195	0.92	13.58	3.97	0.04
A_23_P164141	*PSME3*	NM_176863	10197	0.89	10.79	7.38	0.01
A_23_P156842	*EEF1E1*	NM_004280	9521	0.89	13.42	7.96	0.01
A_33_P3287815	*DDX21*	NM_004728	9188	0.87	13.48	8.11	0.01
A_23_P43726	*NUP160*	NM_015231	23279	0.86	11.39	6.96	0.01
A_21_P0011842	*EEF1E1*	NM_001135650	9521	0.86	13.35	5.47	0.02
A_23_P131954	*SNX5*	NM_014426	27131	0.86	15.01	4.18	0.04
A_23_P148484	*RLIM*	NM_016120	51132	0.86	10.97	3.37	0.06
A_23_P252362	*MRPS30*	NM_016640	10884	0.86	10.49	6.46	0.01
A_24_P134727	*TFAM*	NM_003201	7019	0.83	9.21	7.42	0.01
A_23_P214907	*MTHFD1L*	NM_015440	25902	0.82	9.75	2.11	0.15
A_23_P256148	*AKIRIN1*	NM_024595	79647	0.81	11.04	3.58	0.05
A_33_P3287502	*MSH2*	NM_000251	4436	0.77	11.49	5.50	0.02
A_23_P128991	*SLIRP*	NM_031210	81892	0.77	14.35	2.63	0.10
A_33_P3285444	*TERF1*	NM_017489	7013	0.76	4.75	1.48	0.26
A_24_P50458	*TERF1*	NM_017489	7013	0.76	12.20	3.75	0.05
A_33_P3242659	*KIF13A*	NM_022113	63971	0.70	5.56	3.56	0.05
A_33_P3329108	*MTAP*	NM_002451	4507	0.69	11.71	3.99	0.04
A_23_P333951	*DNAH14*	NM_144989	127602	0.67	9.74	2.67	0.10
A_23_P137484	*L1TD1*	NM_019079	54596	0.67	5.12	5.21	0.02
A_23_P128372	*FKBP4*	NM_002014	2288	0.65	12.78	4.38	0.04
A_33_P3294404	*AKIRIN1*	NM_024595	79647	0.64	10.32	3.56	0.05
A_23_P216149	*TERF1*	NM_017489	7013	0.64	11.57	1.77	0.20
A_23_P102471	*MSH2*	NM_000251	4436	0.59	12.22	5.40	0.02
A_24_P854913	*METTL21A*	NM_001127395	151194	0.56	11.08	1.39	0.28
A_23_P54540	*EIF2AK4*	NM_001013703	440275	0.55	11.76	3.94	0.04
A_23_P94636	*RC3H2*	NM_018835	54542	0.49	9.40	4.46	0.03
A_23_P146997	*TXLNG*	NM_018360	55787	0.48	9.65	1.61	0.23
A_33_P3389188	*TFAM*	NM_003201	7019	0.47	12.74	1.88	0.18
A_33_P3354267	*AKIRIN1*	NM_024595	79647	0.47	11.81	3.21	0.07
A_33_P3283906	*NIP7*	NM_016101	51388	0.46	12.12	3.33	0.06
A_33_P3345504	*RC3H2*	NM_018835	54542	0.45	7.47	4.19	0.04
A_33_P3299776	*NODAL*	NM_018055	4838	0.45	3.81	1.54	0.25
A_32_P220696	*TERF1*	NM_017489	7013	0.45	10.55	1.81	0.19
A_23_P213908	*PHAX*	NM_032177	51808	0.44	13.19	3.43	0.06
A_24_P192434	*TERF1*	NM_017489	7013	0.44	10.44	1.56	0.24
A_33_P3241786	*ADD2*	NM_017482	119	0.40	3.31	1.34	0.29
A_32_P87531	*DNAH14*	NM_001145154	127602	0.35	8.81	1.17	0.35
A_33_P3269453	*BPTF*	ENST00000342579	2186	0.34	10.51	1.74	0.20
A_21_P0000013	*TIMM8A*	NM_001145951	1678	0.34	10.14	2.88	0.08
A_33_P3278118	*CASP3*	NM_004346	836	0.32	8.43	0.86	0.47
A_23_P134008	*USP45*	ENST00000472914	85015	0.31	9.59	1.34	0.29
A_33_P3297978	*MYO1E*	NM_004998	4643	0.30	14.27	2.24	0.13
A_24_P127691	*DNAH14*	ENST00000495456	127602	0.29	4.94	1.91	0.18
A_33_P3289996	*USP45*	NM_001080481	85015	0.27	7.74	2.52	0.11
A_24_P281975	*GNPTAB*	NM_024312	79158	0.25	11.60	1.16	0.35
A_24_P215407	*DDX6*	NM_004397	1656	0.25	8.91	2.20	0.14
A_33_P3289995	*USP45*	ENST00000369232	85015	0.24	4.91	2.23	0.14
A_33_P3409506	*C9orf85*	NM_182505	138241	0.20	8.88	1.66	0.22
A_32_P104478	*FGD6*	NM_018351	55785	0.17	11.00	0.86	0.47
A_33_P3418294	*DNAH14*	NM_001373	127602	0.15	3.15	1.24	0.32
A_24_P51118	*MTAP*	NM_002451	4507	0.14	10.15	0.28	0.80
A_23_P214354	*EXOC2*	NM_018303	55770	0.11	8.27	0.33	0.77
A_33_P3235340	*DDX18*	NM_006773	8886	0.11	13.07	0.98	0.42
A_33_P3269976	*GAL*	ENST00000538401	51083	0.10	2.93	0.84	0.48
A_23_P86504	*C10orf76*	NM_024541	79591	0.10	11.60	0.74	0.53
A_33_P3291976	*TERF1*	ENST00000518695	7013	0.07	5.35	0.46	0.69
A_23_P92410	*CASP3*	NM_004346	836	0.06	14.49	0.52	0.65
A_32_P44775	*C9orf85*	NM_182505	138241	0.05	9.97	0.38	0.73
A_33_P3414669	*RLIM*	NM_183353	51132	0.04	6.26	0.40	0.72
A_23_P351215	*SKIL*	NM_005414	6498	0.04	7.47	0.34	0.76
A_24_P152404	*C10orf76*	ENST00000311122	79591	0.03	10.23	0.16	0.89
A_32_P135243	*MTHFD1L*	NM_015440	25902	0.03	10.60	0.11	0.92
A_32_P80255	*DDX6*	NM_004397	1656	0.03	10.53	0.15	0.89
A_32_P528967	*RTP1*	NM_153708	132112	0.02	3.05	0.18	0.87
A_21_P0013574	*MTHFD1L*	NM_001242767	25902	0.02	11.20	0.10	0.93
A_33_P3378972	*UNC5D*	NM_080872	137970	0.01	3.02	0.12	0.92
A_32_P741851	*GLB1L3*	NM_001080407	112937	0.01	2.96	0.11	0.92
A_23_P140362	*VRTN*	NM_018228	55237	0.01	2.86	0.11	0.92
A_33_P3241782	*ADD2*	NM_001617	119	0.01	2.76	0.08	0.94
A_23_P72817	*GDF3*	NM_020634	9573	0.01	2.91	0.05	0.96
A_23_P329798	*CER1*	NM_005454	9350	0.00	2.67	0.04	0.97
A_23_P5370	*RPRM*	NM_019845	56475	0.00	2.84	0.03	0.98
A_23_P327910	*ZIC3*	NM_003413	7547	0.00	2.83	0.03	0.98
A_33_P3419632	*GLB1L3*	ENST00000389887	112937	0.00	3.57	0.01	1.00
A_23_P216118	*UNC5D*	NM_080872	137970	0.00	2.95	0.02	0.99
A_21_P0014207	*LOC100506507*	XR_108853	NA	0.00	2.66	0.01	0.99
A_23_P380526	*DPPA4*	NM_018189	55211	0.00	2.81	−0.04	0.97
A_23_P421436	*ADD2*	NM_017488	119	−0.01	2.84	−0.05	0.96
A_19_P00318232	*SHISA9*	NM_001145205	729993	−0.01	2.81	−0.06	0.96
A_33_P3280729	*SHISA9*	NM_001145204	729993	−0.01	2.88	−0.06	0.96
A_23_P137573	*LEFTY2*	NM_003240	7044	−0.01	2.87	−0.06	0.96
A_24_P235049	*MTHFD1L*	NM_015440	25902	−0.02	11.42	−0.10	0.93
A_32_P213091	*SHISA9*	NM_001145205	729993	−0.03	4.56	−0.26	0.82
A_23_P375147	*RC3H2*	ENST00000373670	54542	−0.05	11.16	−0.18	0.87
A_24_P380132	*G3BP2*	NM_203505	9908	−0.06	14.44	−0.28	0.80
A_23_P70168	*TARS*	NM_152295	6897	−0.11	14.69	−0.58	0.61
A_23_P79962	*MKKS*	NM_170784	8195	−0.11	12.65	−0.85	0.47
A_23_P84070	*LARP7*	NM_016648	51574	−0.11	12.56	−0.98	0.42
A_33_P3297245	*RRAS2*	NM_012250	22800	−0.12	14.04	−1.06	0.38
A_24_P332230	*LARP7*	NM_016648	51574	−0.12	13.09	−0.72	0.54
A_24_P943922	*CACHD1*	NM_020925	57685	−0.14	4.65	−0.18	0.87
A_33_P3307775	*DENR*	NM_003677	8562	−0.14	7.10	−0.44	0.69
A_33_P3862375	*USP45*	NM_001080481	85015	−0.14	9.20	−0.32	0.78
A_33_P3234317	*RRAS2*	NM_012250	22800	−0.15	13.91	−1.31	0.30
A_33_P3378644	*PHC1*	NM_004426	1911	−0.19	7.01	−1.11	0.37
A_23_P47058	*CUZD1*	NM_022034	50624	−0.22	8.19	−1.04	0.39
A_23_P215484	*CCL26*	NM_006072	10344	−0.26	4.06	−1.35	0.29
A_23_P427217	*JMJD1C*	NM_032776	221037	−0.46	9.97	−3.89	0.05
A_23_P346265	*GNPTAB*	NM_024312	79158	−0.47	9.02	−1.41	0.28
A_24_P940125	*CNOT6*	NM_015455	57472	−0.50	11.75	−4.61	0.03
A_33_P3295523	*RAC3*	NM_005052	5881	−0.50	12.37	−3.43	0.06
A_23_P25587	*LECT1*	NM_007015	11061	−0.51	4.69	−1.91	0.18
A_24_P347624	*SNURF*	NM_022804	8926	−0.52	13.32	−1.90	0.18
A_23_P204246	*PHC1*	NM_004426	1911	−0.55	4.48	−0.98	0.42
A_23_P259127	*ESRP1*	NM_017697	54845	−0.60	11.38	−1.91	0.18
A_23_P366376	*TDGF1*	NM_003212	6997	−0.65	7.90	−2.86	0.08
A_24_P144601	*POU5F1*	NM_002701	5460	−0.66	7.98	−2.07	0.15
A_23_P156809	*METTL21A*	NM_001127395	151194	−0.66	11.71	−5.71	0.02
A_24_P104538	*BPTF*	ENST00000342579	2186	−0.67	9.15	−2.77	0.09
A_21_P0000084	*SLC25A21*	NM_030631	89874	−0.68	3.26	−1.42	0.27
A_23_P72770	*USP44*	NM_032147	84101	−0.79	7.71	−7.30	0.01
A_33_P3309206	*GABRB3*	ENST00000556166	2562	−0.87	4.56	−7.79	0.01
A_23_P59138	*POU5F1*	NM_002701	5460	−0.99	12.96	−6.27	0.02
A_33_P3227506	*BPTF*	NM_182641	2186	−1.01	9.64	−6.30	0.02
A_33_P3277075	*GABRB3*	NM_000814	2562	−1.04	8.28	−9.63	0.01
A_24_P52921	*BCAT1*	NM_005504	586	−1.06	3.48	−4.10	0.04
A_24_P314477	*TUBB2B*	NM_178012	347733	−1.14	7.23	−10.24	0.01
A_23_P323094	*PHC1*	NM_004426	1911	−1.24	6.06	−4.36	0.04
A_33_P3242014	*PHC1*	NM_004426	1911	−1.26	10.65	−9.28	0.01
A_23_P204640	*NANOG*	NM_024865	79923	−1.66	7.94	−7.10	0.01
A_24_P935986	*BCAT1*	NM_005504	586	−1.77	9.14	−9.18	0.01
A_23_P160336	*LEFTY1*	NM_020997	10637	−2.93	4.40	−21.49	0.00

a NA, not applicable.

We next tested whether the activation of the Wnt/β-catenin pathway is essential for maintenance of GB epithelial stem cells since they are phenotypically similar to adult cholangiocytes of the intrahepatic duct, which require activation of the LGR5 receptor by the Wnt agonist R-spondin for long-term culture (30). The fraction of cells able to give rise to new organoids remained at 3 to 4% for at least 10 passages for human cells only if R-spondin was added, irrespective of the presence of Wnt3A in the culture medium (Fig. 1D), and at 7 to 9% for 19 passages for murine organoids (see Fig. S1C) (30). Since R-spondins usually act synergistically with Wnt ligands, we next tested whether the epithelial cells themselves produce such ligands. Blocking Wnt ligand secretion through addition of the porcupine inhibitor IWP2 inhibited organoid formation from single cells (Fig. 1E). Organoid formation was partially rescued by the addition of exogenous Wnt3a, suggesting that GB epithelial cells or a subset of them might secrete Wnt agonists. Such a mechanism has been shown in mouse small intestinal organoids, where Paneth cells produce Wnt ligands, supporting organoid growth in the absence of exogenous Wnt agonists (43). Whether a similar subpopulation of cells is responsible for Wnt ligand production in the gallbladder is currently not known.

10.1128/mBio.01911-20.1 FIG S1 Cultivation of murine gallbladder organoids. (A) Murine gallbladder epithelial cells grown as organoids at 1, 2, and 4 days after seeding, and at passages 1, 5, 10, 16, and 19. Scale bar, 1mm. (B) Organoids on day 7 after seeding, fluorescently labeled with antibodies against the proliferation marker PCNA (green) and β-catenin (red); nuclei were stained with DRAQ5 (blue). Scale bar, 50 μm. (C) Organoids at passage 0 were split to single cells, seeded, and the number of resulting organoids was counted 5 to 7 days later (i.e., at passage 1). The organoids were kept in culture, and the procedure was repeated after 18 passages (i.e., at passage 19). Bars represent means ± the SD. (D) Lineage tracing of organoids derived from Lgr5-EGFP-IRES-CreERT2, ROSA-mTmG^floxed^ reporter mice after HT induction. Organoids derived from Lgr5^+^ cells express mGFP, while those derived from Lgr5^−^ cells express mTomato. Scale bar, 200 μm. Download FIG S1, TIF file, 2.1 MB. Copyright © 2020 Sepe et al. 2020 Sepe et al. This content is distributed under the terms of the Creative Commons Attribution 4.0 International license.

We next found that *WNT3*, *-4*, *-7A*, *-7B*, and *-11* were expressed in GB organoids, but only *WNT7A* and *WNT7B* were significantly overexpressed in the stem cell-enriched early organoids, whereas late organoids were enriched in *WNT4* (Fig. 1F). This indicates that different types of cells are secreting specific Wnt proteins and that WNT7A and B might play a specific role in stem cell maintenance, since they are abundantly expressed in early organoids (Fig. 1F).

Since the activation of the Wnt/β-catenin pathway is essential for stem cell maintenance we expected to find higher levels of target gene transcription in stem cells. We compared a published list of β-catenin target genes (44) with the results of our microarray (Table 3) and observed a dramatic enrichment of such genes in early organoids compared to older, more differentiated organoids (Fig. 1G). The most relevant differentially regulated genes were the secreted Wnt inhibitors Dickkopf-1 (*DKK1*) and *DKK4*, the transcription factor binding to nuclear β-catenin *LEF1*, and *LGR5*. In differentiated organoids, we observed upregulated expression of the intracellular Wnt inhibitor *AXIN2*, which may play a role in inhibiting the pathway in more differentiated cells (Table 3).

**TABLE 3:** List of differentially regulated β-catenin target genes in early organoids versus late organoids

Probe	Gene symbol	RefSeq	Entrez ID	logFC	Avg expression	*t* score	*P*
A_23_P118815	*BIRC5*	NM_001012271	332	4.68	13.18	42.25	0.00
A_23_P94275	*DKK4*	NM_014420	27121	3.40	6.21	16.98	0.00
A_23_P24129	*DKK1*	NM_012242	22943	3.17	14.38	19.03	0.00
A_24_P20630	*LEF1*	NM_016269	51176	2.02	5.96	14.91	0.00
A_33_P3329187	*DNMT1*	NM_001130823	1786	1.69	12.38	10.55	0.00
A_23_P159191	*GAST*	NM_000805	2520	1.58	7.69	5.45	0.02
A_23_P98974	*LGR5*	NM_003667	8549	1.54	5.41	8.18	0.01
A_33_P3258392	*EDN1*	NM_001955	1906	1.49	12.07	3.21	0.07
A_23_P214821	*EDN1*	NM_001955	1906	1.48	14.90	7.19	0.01
A_23_P202837	*CCND1*	NM_053056	595	1.44	11.40	3.97	0.04
A_33_P3232828	*SRSF3*	NM_003017	6428	1.44	13.02	6.81	0.01
A_23_P215956	*MYC*	NM_002467	4609	1.37	14.06	6.25	0.02
A_23_P24104	*PLAU*	NM_002658	5328	1.16	14.30	6.33	0.02
A_33_P3306146	*PLAU*	NM_001145031	5328	1.10	9.48	2.68	0.10
A_23_P160968	*LAMC2*	NM_018891	3918	1.08	10.17	3.70	0.05
A_23_P413761	*SRSF3*	NM_003017	6428	1.03	15.83	8.66	0.01
A_33_P3411075	*FSCN1*	NM_003088	6624	0.99	15.10	7.56	0.01
A_23_P19673	*SGK1*	NM_005627	6446	0.94	12.66	4.51	0.03
A_23_P135381	*SP5*	NM_001003845	389058	0.87	13.23	7.77	0.01
A_33_P3381751	*TIAM1*	NM_003253	7074	0.86	11.85	6.00	0.02
A_33_P3301514	*NRCAM*	NM_001193582	4897	0.85	6.87	3.77	0.05
A_23_P201636	*LAMC2*	NM_005562	3918	0.83	15.34	6.35	0.02
A_23_P94800	*S100A4*	NM_002961	6275	0.81	12.16	7.43	0.01
A_32_P69368	*ID2*	NM_002166	3398	0.72	12.89	2.62	0.10
A_23_P54144	*BMP4*	NM_001202	652	0.71	11.68	2.37	0.12
A_23_P201711	*S100A6*	NM_014624	6277	0.71	17.59	4.76	0.03
A_23_P143143	*ID2*	NM_002166	3398	0.64	12.84	5.39	0.02
A_23_P16469	*PLAUR*	NM_001005377	5329	0.64	11.83	2.75	0.09
A_33_P3294509	*CD44*	NM_000610	960	0.61	15.41	5.07	0.03
A_23_P359245	*MET*	NM_000245	4233	0.60	15.75	4.42	0.03
A_23_P58788	*CDX1*	NM_001804	1044	0.58	3.62	4.50	0.03
A_33_P3332414	*ABCB1*	NM_000927	5243	0.53	9.18	4.36	0.04
A_23_P57784	*CLDN1*	NM_021101	9076	0.52	13.42	4.78	0.03
A_24_P252364	*NRCAM*	NM_001037132	4897	0.49	10.83	1.64	0.22
A_24_P303989	*BMI1*	NM_005180	648	0.41	8.43	3.63	0.05
A_23_P201655	*MYCBP*	NM_012333	26292	0.39	13.57	2.96	0.08
A_23_P412389	*FGF18*	NM_003862	8817	0.35	10.37	2.58	0.10
A_23_P210763	*JAG1*	NM_000214	182	0.35	11.81	3.07	0.07
A_23_P344555	*NEDD9*	NM_006403	4739	0.34	8.83	1.21	0.33
A_23_P314115	*BMI1*	NM_005180	648	0.31	10.15	1.20	0.34
A_23_P214681	*PPARD*	NM_006238	5467	0.31	5.70	1.13	0.36
A_33_P3374443	*L1CAM*	NM_024003	3897	0.31	4.29	1.05	0.39
A_23_P100883	*SUZ12*	NM_015355	23512	0.30	13.86	1.12	0.36
A_33_P3323298	*JUN*	NM_002228	3725	0.28	12.78	2.51	0.11
A_23_P138631	*SMC3*	NM_005445	9126	0.27	12.56	1.97	0.17
A_23_P82523	*ABCB1*	NM_000927	5243	0.27	12.31	2.01	0.16
A_24_P207995	*L1CAM*	NM_000425	3897	0.26	3.50	0.58	0.61
A_32_P171061	*ASCL2*	NM_005170	430	0.23	9.18	1.38	0.28
A_21_P0000152	*CD44*	NM_001202557	960	0.21	6.02	0.65	0.57
A_33_P3243857	*ADAM10*	NM_001110	102	0.19	11.61	1.42	0.27
A_23_P31073	*MYB*	NM_005375	4602	0.19	12.36	1.03	0.40
A_23_P26847	*SOX9*	NM_000346	6662	0.16	10.69	1.17	0.35
A_24_P69095	*ENC1*	NM_003633	8507	0.13	13.73	0.20	0.86
A_33_P3289848	*CDX1*	NM_001804	1044	0.11	8.23	0.72	0.54
A_23_P402751	*COX2*	ENST00000361739	4513	0.08	15.40	0.28	0.80
A_33_P3880302	*EPHB2*	NM_004442	2048	0.06	7.27	0.28	0.80
A_24_P252130	*PPARD*	NM_006238	5467	0.06	11.98	0.50	0.66
A_33_P3245163	*MYC*	M13930	4609	0.05	3.06	0.42	0.71
A_33_P3311795	*MYB*	ENST00000531845	4602	0.02	2.96	0.19	0.86
A_24_P365807	*EFNB1*	NM_004429	1947	−0.03	15.21	−0.29	0.80
A_24_P82106	*MMP14*	NM_004995	4323	−0.05	10.03	−0.36	0.75
A_23_P48886	*ADAM10*	NM_001110	102	−0.06	10.76	−0.55	0.63
A_33_P3370787	*EPHB2*	NM_004442	2048	−0.18	8.02	−1.69	0.21
A_23_P6596	*HES1*	NM_005524	3280	−0.19	7.93	−1.74	0.20
A_23_P95060	*EPHB3*	NM_004443	2049	−0.19	11.52	−0.99	0.41
A_33_P3331376	*EPHB2*	NM_004442	2048	−0.22	5.58	−1.61	0.23
A_33_P3411628	*CDKN2A*	NM_000077	1029	−0.33	10.69	−3.01	0.08
A_23_P52207	*BAMBI*	NM_012342	25805	−0.40	14.60	−3.50	0.06
A_21_P0014167	*NEDD9*	ENST00000379433	4739	−0.41	4.24	−2.81	0.09
A_23_P27332	*TCF4*	NM_003199	6925	−0.50	7.67	−3.83	0.05
A_33_P3258824	*NOTCH2*	NM_001200001	4853	−0.54	12.30	−2.72	0.09
A_24_P298027	*AXIN2*	NM_004655	8313	−0.56	7.02	−2.16	0.14
A_23_P43490	*CDKN2A*	NM_058197	1029	−0.60	12.54	−4.48	0.03
A_33_P3368358	*NEDD9*	NM_182966	4739	−0.64	8.82	−4.65	0.03
A_23_P418373	*BCL2L2*	NM_004050	599	−0.68	12.42	−6.26	0.02
A_23_P148015	*AXIN2*	NM_004655	8313	−0.71	10.85	−4.42	0.03
A_23_P200792	*NOTCH2*	NM_024408	4853	−1.06	13.84	−8.94	0.01
A_23_P52761	*MMP7*	NM_002423	4316	−1.10	16.35	−5.16	0.02
A_23_P502464	*NOS2*	NM_000625	4843	−2.40	4.30	−9.83	0.01

Finally, to verify that expansion of GB organoids is driven by Lgr5^+^ cells, we took advantage of a Lgr5^–^EGFP-IRES-creERT2:ROSA-mTmG-floxed reporter mouse. In the gallbladder cells of this mouse, Cre-ERT2 is under the control of the *Lgr5* promoter. After induction with 4-hydroxytamoxifen (4HT), Lgr5^+^ cells switch from red-Tomato to green-GFP expression. Induction with 4HT during culture of organoids derived from GBs of the reporter mice resulted in the generation of two distinct organoid populations. The majority derived from Lgr5^−^ cells expressing mTomato, while 8.6% originated from Lgr5^+^ cells expressing mGFP (Fig. 1H; see also Fig. S1D in the supplemental material). The proportion of organoids derived from Lgr5^+^ cells steadily increased after the first passage, making up >90% by passage 4, confirming the crucial role of Wnt/β-catenin signaling through the Lgr5 receptor in the long-term maintenance of GB cells *in vitro*.

### Gallbladder organoids are stable and resemble the cell structure and function of the organ *in situ*.

**FIG 2:**
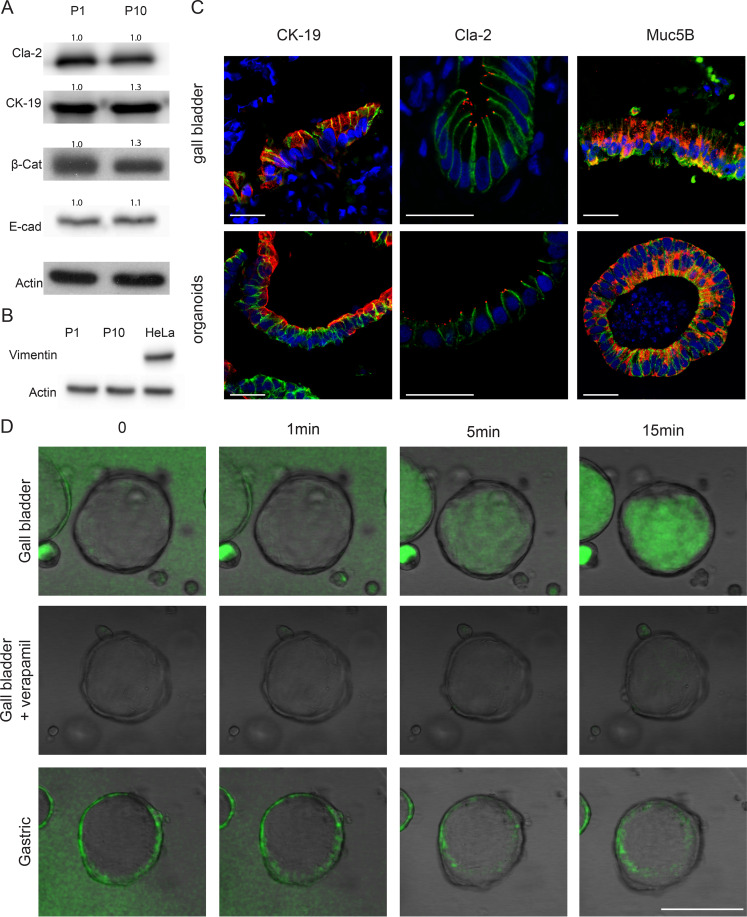
Characterization of human organoids. (A) Western blot analysis of epithelial and gallbladder markers at early (P1) and late (P10) passages. Relative densitometry values, normalized to P1 (=1), are shown above the bands. (B) Western blot analysis as in panel A of the fibroblast marker Vimentin compared to HeLa cells. (C) Immunofluorescence analysis of human gallbladder tissue and organoids 7 days after seeding for the gallbladder markers cytokeratin-19, claudin-2, or mucin5B (red); the epithelial marker E-cadherin (green); and DRAQ5 (blue). Scale bar, 25 μm. (D) Transport assay of rhodamine-123 (green) in gallbladder organoids treated with the multidrug transporter inhibitor verapamil (middle row), and gastric organoids. Scale bar, 100 μm.

To confirm that GB organoids maintain their epithelial identity, we examined expression of the epithelial marker E-cadherin by Western blot (Fig. 2A). The levels of the GB markers claudin-2 and cytokeratin-19 did not change between early (passage 1) and late (passage 10 for human, 19 for mouse) passages (see Fig. 2A for humans and see Fig. S2A for mice). Previous attempts to cultivate epithelial primary cells were frustrated by fibroblast outgrowth (45, 46). In our system, we observed that fibroblasts do not grow in Matrigel, and at the end of passage 1 we could not detect the mesenchymal marker Vimentin (Fig. 2B and Fig. S2B). In order to assess the GB identity of organoids, we used fluorescence immunohistochemistry to examine a GB-specific combination of markers and compared their expression to that of GB tissue. The luminal mucosa of the GB consists of a simple columnar epithelium expressing cytokeratin-19 (47). Similarly, the GB organoids consist of an E-cadherin-positive cell monolayer, with apical cytokeratin-19 expression (Fig. 2C for humans and Fig. S2C for mice, left panel) and eccentric nuclei (Fig. 2C for humans and Fig. S2C for mice). These organoids also show luminal junctional expression of claudin-2 (Fig. 2C for humans and Fig. S2C for mice), a tight-junction protein expressed at higher levels in the gallbladder compared to other organs including the cholangiocytes of the bile duct (48). GB epithelial cells also produce mucins, with MUC5B being one of the most abundant (49, 50). As expected, we detected MUC5B expression in both the tissue sample and the organoids (Fig. 2C for humans and Fig. S2C for mice).

10.1128/mBio.01911-20.2 FIG S2 Characterization of murine gallbladder organoids. (A) Western blot analysis of murine epithelial and gallbladder markers at early (P1) and late (P19) passages. (B) Western blot analysis as in panel A of the fibroblast marker vimentin compared to HeLa cells. (C) Immunofluorescence analysis of murine gallbladder tissue and organoids at 7 days after seeding for the gallbladder markers cytokeratin-19, claudin-2, or mucin5B (red); the epithelial marker E-cadherin (green); and DRAQ5 (blue). Scale bar, 10 μm. Download FIG S2, TIF file, 1.3 MB. Copyright © 2020 Sepe et al. 2020 Sepe et al. This content is distributed under the terms of the Creative Commons Attribution 4.0 International license.

One of the functions of the GB is to concentrate bile in the lumen (37, 38). The gallbladder expresses the ATP-dependent multidrug transporter MDR1, which transports organic cations back into the lumen (51–53), protecting the organ from high concentrations of potentially toxic organic ions. To test whether gallbladder organoids functionally recapitulate this physiological feature, we added rhodamine-123, a chemical dye substrate of MDR1 often used to monitor organoid function, to the medium (54). Gallbladder organoids actively transported the dye into the lumen, resulting in increased concentration of luminal fluorescence relative to the medium on the outside (Fig. 2D, top panel). Pretreating organoids with the MDR1 inhibitor verapamil prevented luminal dye accumulation (Fig. 2D, middle panel), confirming dependence on MDR1. In contrast, gastric organoids did not accumulate the dye (Fig. 2D, bottom panel).

### *Salmonella enterica* serovar Paratyphi A induces paracrine CdtB-dependent DNA damage in GB organoids.

Since the gallbladder organoids accurately recapitulate the main molecular features of the epithelium of origin, we used them to model infection with S. enterica using the human restricted pathogenic serovar Paratyphi A, which has been epidemiologically linked to gallbladder cancer (7, 55). Previous observations of the genotoxic effects of *S.* Typhi/Paratyphi A were based on experiments in cell lines, using mostly ectopic expression of recombinant typhoid toxin (19, 20).

**FIG 3:**
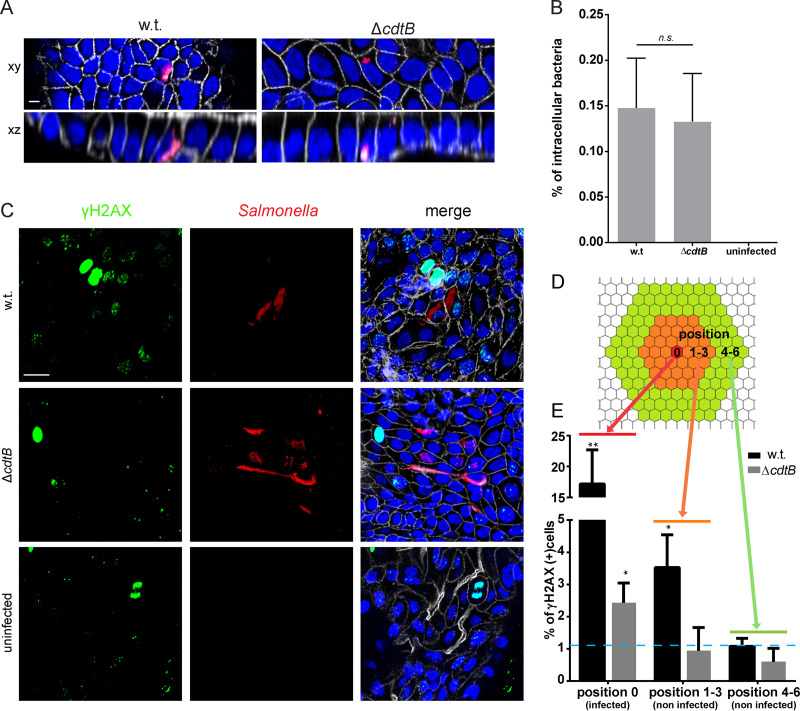
Infection and paracrine genotoxic effect of CdtB. (A) Reconstruction of whole-mount immunofluorescence labeling of organoids infected with *Salmonella* Paratyphi A carrying the mCherry-expressing plasmid pLS002 (red) at 3 days post infection, with phalloidin to detect F-actin (white) and Hoechst for DNA (blue). Scale bar, 10 μm. (B) Proportion of cells invaded after infection of organoids with wild-type *Salmonella* or a *cdtB* deletion mutant. (C) Whole-mount immunofluorescence labeling of organoids 3 days after infection with *Salmonella* Paratyphi A w.t. or Δ*cdtB* carrying the mCherry-expressing plasmid pLS002 using antibodies against γH2AX (green), phalloidin (white), and Hoechst (blue). Scale bar, 20 μm. (D) Model for categorization of uninfected cells according to the distance from the infected cell at position 0 (red). Orange represents the first three rings of non-infected cells (positions 1 to 3), and green represents the next three rings (positions 4 to 6). (E) Percentage of cells positive for the DNA damage marker γH2AX depending on their distance from the infected cell. The dashed blue line represents the average percentage of γH2AX-positive cells in uninfected organoids (SD = 0.97). *, *P* < 0.05; **, *P* < 0.01 (compared to uninfected cells). Infected cells are defined as cells with >5 bacteria, and γH2AX-positive cells are cells with >3 foci.

Since the genotoxicity of the bacterium resides in the CdtB subunit of the typhoid toxin, we generated a *cdtB* knockout. Organoids were mechanically sheared to expose the luminal side and cocultured with Salmonella enterica serovar Paratyphi A or with its isogenic *cdtB* knockout strain, before reseeding in Matrigel, with gentamicin-supplemented medium to eliminate extracellular bacteria. At 3 days post infection, organoids showed foci of infection with intracellular *Salmonella* (Fig. 3A). After verifying that the Δ*cdtB* mutant is capable of invading epithelial cells at a rate similar to the wild-type (w.t.) bacteria (Fig. 3B), we examined the induction of DNA damage.

To this end, we tested organoids for phosphorylation of H2AX at serine 139 (γH2AX), a histone variant involved in detection of DSBs and recruitment of repair factors (56), and we quantified and mapped the number of γH2AX-positive cells after infection with the wild type and the Δ*cdtB* strain. The number of cells experiencing DNA damage was generally higher in the organoids infected with the wild-type strain compared to the Δ*cdtB* mutant (Fig. 3C). Quantification of the number of γH2AX-positive cells that are infected (defined in the map of Fig. 3D as position 0) revealed that both cells infected with the w.t. or Δ*cdtB* strain experience DNA damage (Fig. 3E, position 0). However, there is a significantly reduced number of γH2AX-positive cells among the ones infected with the mutant strain (Fig. 3E, position 0, Δ*cdtB*).

In addition, we noticed that in organoids infected with the wild-type strain, a number of uninfected neighboring cells also contained γH2AX foci (Fig. 3C to E). To quantify this paracrine genotoxic effect, uninfected cells were divided into two groups depending on the distance from the infected cell (Fig. 3D): Positions 1 to 3 include the first three rings of uninfected cells surrounding the infected focus, whereas positions 4 to 6 represent the rings 4 to 6 of the uninfected cells. The proportion of γH2AX-positive cells was higher in positions 1 to 3 than in positions 4 to 6 (Fig. 3E), but only for the organoids infected with wild-type bacteria. This confirms that the typhoid toxin is secreted from infected cells also in the primary polarized cells of the organoids (17) and that its genotoxic effects extend to the neighboring cells in a paracrine manner. In our system, this paracrine effect was limited to the first three rings of cells surrounding the infected one. Since γH2AX is also highly expressed during mitosis, cells that displayed chromosome condensation were excluded from the analysis. Our experiments suggest that infection with *Salmonella* Paratyphi A causes DNA damage and that a functional typhoid toxin increases the extent of damage in the infected cells and extends it to the neighboring uninfected cells.

### Infection with *Salmonella* Paratyphi A activates transcription programs associated with cell cycle arrest.

**FIG 4:**
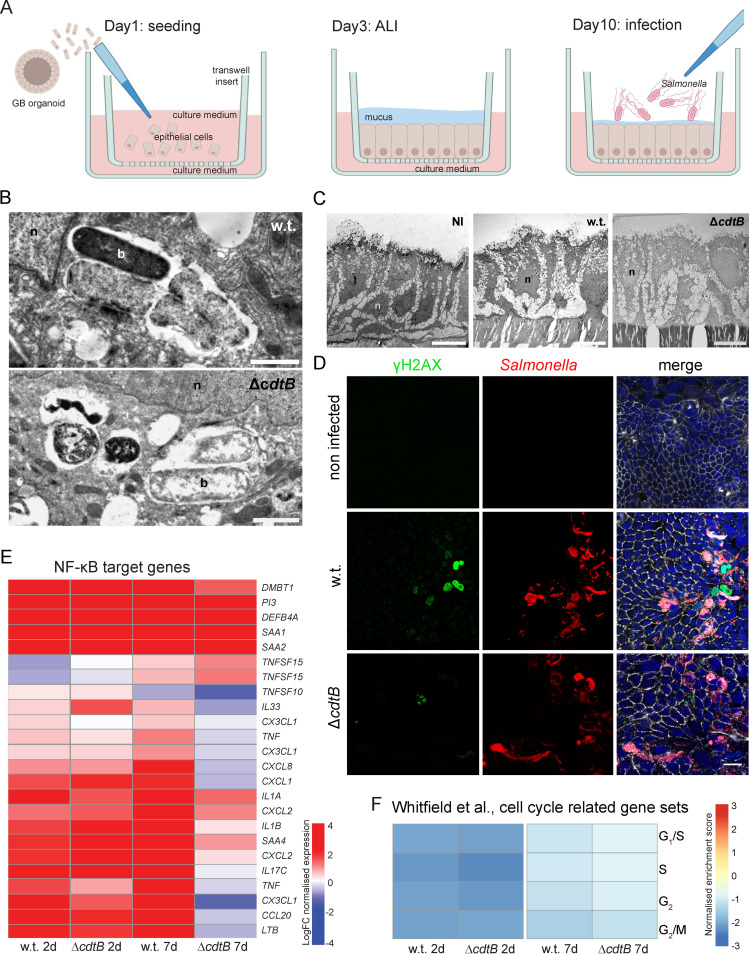
Generation of gallbladder mucosoids and long-term infection experiments. (A) Schematic of gallbladder mucosoid cultivation and infection procedure. (From left to right) After seeding, a polarized cell layer of gallbladder cells begins to form on the collagen-coated polycarbonate filter in the transwell insert. Primary cell medium is provided around the cell culture insert and on top of the cells. At day 3, the upper medium is withdrawn, and cells start to produce mucus. From day 10 onward, the culture is stable, and infection experiments can be performed by administering *Salmonella* on the cell layer. (B) Detailed view of long-term infection of human gallbladder mucosoids with S. enterica Paratyphi A and transmission electron microscopy. Stable long-term infection can be reached with both the wild type and the *cdtB* deletion mutant by applying gentamicin for 24 h and then withdrawing it again from the medium. Internalization and perinuclear localization of the bacteria within lysosomal structures is visible. Two zoomed-in images of intracellular bacteria are shown. b, bacterium; n, nucleus. Scale bar, 1 μm. (C) Establishment of mucosoids. The development of a polarized monolayer of gallbladder cells in an air-liquid cultivation (“mucosoids”) and transmission electron microscopy images of non-infected control (NI) and infected with *S.* Paratyphi A w.t. and isogenic Δ*cdtB* KO strains for 2 days are shown. Scale bar, 10 μm. (D) Top view of infected and non-infected gallbladder mucosoids. Staining was performed for γH2AX (green), *Salmonella* (red), phalloidin (white), and nuclei (blue). Cultures infected for 6 days show DNA damage, whereas there is no damage visible in the non-infected control. Scale bar, 20 μm. (E) Heat map of manually selected NF-κB target genes. A comparison of w.t. and Δ*cdtB* infections at 2 and 7 days post infection is shown. The heatmap was plotted using the normalized expression values (log-normalized intensity) relative to the non-infected control at each time point (logFC). (F) Heatmap of normalized enrichment scores from GSEA for genes preferentially expressed in distinct cell cycle phases (58) for comparisons of mucosoid cultures with w.t. or Δ*cdtB* strain infections at 2 and 7 days post infection relative to non-infected controls.

The risk of developing gallbladder cancer is higher in patients who are chronic carriers of typhoid *Salmonella* serovars. Therefore, to understand the fate of the infected cells, we sought to extend the duration of the infection using a more physiological model that mimics chronic infection *in vitro*. For the infection of the organoids, the cells must be disaggregated, and after 3 days we usually observed an overgrowth of bacteria or of cells, which impaired longer-term analysis. To understand the effect of the infection on a homeostatic gallbladder epithelial barrier and to allow longer term infection, we adapted the gallbladder organoids into mucosoid cultures, as previously done for the human stomach (34). Single cells derived from organoids were seeded on a collagen-coated polycarbonate filter in a standing cell culture insert (Fig. 4A). The cultivation cocktail was identical to that used for organoids and applied both below and above the filter. After 3 days, the apical medium was removed to start air-liquid interface cultivation (Fig. 4A). Primary gallbladder cells can be expanded on a monthly basis by deriving single cells from mucosoid cultures and restarting from the seeding procedure. Gallbladder mucosoids can be infected by applying a suspension of bacteria on top of the filter after removing excess mucus (Fig. 4A). The progress of the infection can be monitored using fluorescent transgenic S*almonella.* Presence of intracellular *Salmonella* was detectable equally for both wild-type and *ΔcdtB* strains (Fig. 4B), and electron microscopy analysis of non-infected and infected mucosoid cultures revealed that the monolayer and the cell gross morphology remain intact during infection (Fig. 4C).

Similar to what we observed with organoids, in the infected mucosoid cultures, we found that established colonies of w.t. *Salmonella* induce more DNA damage than the isogenic Δ*cdtB* strain, as measured using gH2AX staining (Fig. 4D). We performed a microarray analysis to compare the short versus the long-term effect of the infection on the gallbladder epithelial cells. We used gene set enrichment analysis (GSEA) to investigate any statistically significant consistent differences between gene set expression in the culture after infection with the w.t. strain versus infection with the Δ*cdtB* isogenic mutant. Infection with both strains induced similar expression of NF-κB target genes at 2 days post infection, indicating the expected initiation of an inflammatory response (Fig. 4E). Interestingly, in the cultures infected with the w.t. strain, NF-κB-controlled cytokines and chemokine genes continued to be highly expressed at 7 days, suggesting a role of the typhoid toxin in maintaining inflammation. It has previously been observed that the typhoid toxin reduces inflammation in mice infected with a transgenic *Salmonella* Typhimurium strain expressing the typhoid toxin (57). Inflammation is the result of a complex interaction between immune cells and the epithelium in the mucosa, and we observed here that typhoid toxin directly or indirectly maintains high transcription of NF-κB target genes in epithelial cells.

Analysis of the cell-cycle related gene sets (58) during infection (Table 4) revealed a strong underrepresentation of transcriptional programs related to each cell cycle phase (G_1_/S, S, G_2_, and G_2_/M) (Fig. 4F). As those genes are usually accumulated only in a specific phase of the cell cycle, the downregulation of all the G_1_/S, S, G_2_, and G_2_/M transcription programs implies that a proportion of cells in the infected mucosoids are not replicating (58, 59). This effect of the infection in stopping cell replication is particularly strong at 2 days after infection, but is attenuated after 1 week, indicating that an increasing number of cells are cycling again (Fig. 4F). The effect of the infection on the cell cycle was either independent from a functional typhoid toxin or any effect of the typhoid toxin on the infected culture was masked by other bacterial effectors.

**TABLE 4:** List of differentially regulated genes^*a*^

Probe	Gene symbol	RefSeq	Entrez ID	logFC at:				Cell cycle phase^*b*^
				2 days		7 days		
				w.t. vs NI	dCdtB vs NI	w.t. vs NI	dCdtB vs NI	
A_23_P39481	*ABCA7*	NM_019112	10347	−0.22	−0.02	0.20	−0.66	G1_S
A_23_P26375	*ACD*	NM_022914	65057	−0.11	−0.01	0.10	−0.48	G1_S
A_23_P163143	*ACYP1*	NM_203488	97	0.05	−0.13	−0.18	0.71	G1_S
A_23_P211039	*ADAMTS1*	NM_006988	9510	0.46	0.38	−0.08	0.45	G1_S
A_23_P342275	*ADAMTS1*	NM_006988	9510	0.37	0.16	−0.22	0.48	G1_S
A_24_P283395	*ADCK2*	NM_052853	90956	−0.25	−0.29	−0.03	−0.55	G1_S
A_24_P291978	*ADCK2*	NM_052853	90956	0.23	−0.02	−0.26	−0.10	G1_S
A_23_P501547	*ADCY6*	NM_015270	112	−0.21	0.10	0.31	−0.73	G1_S
A_24_P298077	*ANKRD10*	NM_017664	55608	0.75	−0.03	−0.78	0.94	G1_S
A_23_P205046	*ANKRD10*	NM_017664	55608	0.03	0.56	0.53	−0.36	G1_S
A_23_P170331	*AP3M2*	NM_006803	10947	0.34	0.04	−0.30	0.60	G1_S
A_24_P64039	*AP3M2*	NM_006803	10947	−0.02	−0.32	−0.30	0.47	G1_S
A_23_P160729	*AP4B1*	NM_006594	10717	0.31	0.28	−0.03	0.46	G1_S
A_23_P256682	*APEX2*	NM_014481	27301	−0.22	0.20	0.42	−0.34	G1_S
A_23_P162782	*ARGLU1*	NM_018011	55082	0.17	−0.09	−0.25	0.74	G1_S
A_24_P159648	*BAIAP2*	NM_006340	10458	−0.33	−0.03	0.30	−0.93	G1_S
A_23_P315836	*BAIAP2*	NM_017451	10458	0.16	0.11	−0.06	−0.12	G1_S
A_23_P61810	*BAIAP2*	NM_017450	10458	−0.07	0.34	0.41	−1.21	G1_S
A_23_P67771	*BARD1*	NM_000465	580	−1.44	−1.07	0.37	−0.47	G1_S
A_32_P18824	*BRD7*	NM_013263	29117	0.04	−0.13	−0.17	0.07	G1_S
A_23_P381378	*CAPN7*	NM_014296	23473	0.00	−0.06	−0.06	0.20	G1_S
A_23_P58898	*CASP8AP2*	NM_012115	9994	−0.42	−0.45	−0.03	0.12	G1_S
A_32_P180315	*CCDC180*	NM_020893	1E+08	−0.65	−0.98	−0.33	−0.36	G1_S
A_24_P280706	*CCDC180*	NM_020893	1E+08	0.27	0.28	0.01	0.50	G1_S
A_23_P209200	*CCNE1*	NM_001238	898	−0.40	−0.33	0.08	0.17	G1_S
A_23_P215976	*CCNE2*	NM_057749	9134	−1.03	−0.94	0.10	−0.63	G1_S
A_24_P397107	*CDC25A*	NM_001789	993	−1.34	−1.13	0.21	−0.75	G1_S
A_23_P121423	*CDC25A*	NM_001789	993	−0.57	−0.96	−0.38	0.06	G1_S
A_23_P49972	*CDC6*	NM_001254	990	−1.14	−1.40	−0.26	0.39	G1_S
A_23_P251421	*CDCA7*	NM_031942	83879	−1.06	−0.35	0.72	−0.88	G1_S
A_24_P171549	*CDCA7*	NM_031942	83879	−1.00	−0.16	0.84	−1.13	G1_S
A_24_P274795	*CDCA7L*	NM_018719	55536	−1.39	−0.68	0.71	−0.92	G1_S
A_23_P20752	*CDK20*	NM_001039803	23552	0.20	0.17	−0.02	−0.03	G1_S
A_24_P53519	*CHAF1A*	NM_005483	10036	−0.37	−0.42	−0.05	−0.71	G1_S
A_23_P57306	*CHAF1B*	NM_005441	8208	−0.81	−0.45	0.37	−0.54	G1_S
A_23_P126212	*CLSPN*	NM_022111	63967	−0.82	−1.06	−0.24	−0.08	G1_S
A_23_P52556	*CTSD*	NM_001909	1509	−0.32	−0.47	−0.15	−0.61	G1_S
A_23_P139312	*DHFR2*	NM_176815	200895	−0.54	−0.71	−0.17	0.09	G1_S
A_24_P186065	*DHFR2*	NM_176815	200895	−0.05	0.11	0.16	−0.33	G1_S
A_24_P219024	*DIS3*	NM_014953	22894	0.08	0.00	−0.08	0.31	G1_S
A_23_P48416	*DIS3*	NM_014953	22894	0.05	0.17	0.12	−0.27	G1_S
A_24_P395317	*DIS3*	NM_014953	22894	0.01	−0.06	−0.07	−0.29	G1_S
A_23_P36962	*DNAJC3*	NM_006260	5611	0.10	0.13	0.03	0.34	G1_S
A_23_P10385	*DTL*	NM_016448	51514	−2.06	−1.93	0.13	−1.18	G1_S
A_23_P80032	*E2F1*	NM_005225	1869	−1.89	−1.24	0.65	−1.39	G1_S
A_23_P408955	*E2F2*	NM_004091	1870	−2.73	−1.92	0.80	−2.19	G1_S
A_23_P125990	*E2F2*	NM_004091	1870	−0.11	−0.52	−0.40	0.71	G1_S
A_23_P44932	*EIF2A*	NM_032025	83939	0.33	0.14	−0.19	0.46	G1_S
A_32_P197524	*EIF2A*	NM_032025	83939	0.17	−0.18	−0.35	0.32	G1_S
A_23_P87964	*ESD*	NM_001984	2098	−0.33	−0.23	0.11	0.29	G1_S
A_24_P841662	*ESD*	AK093643	2098	−0.38	−0.21	0.17	−0.17	G1_S
A_24_P332314	*FAM111B*	NM_198947	374393	−2.21	−2.52	−0.31	−0.18	G1_S
A_23_P409516	*FAM122A*	NM_138333	116224	0.03	−0.28	−0.31	0.34	G1_S
A_23_P71644	*FANCG*	NM_004629	2189	−0.62	−0.62	−0.01	−0.03	G1_S
A_23_P141146	*FBXL20*	NM_032875	84961	0.56	0.06	−0.49	0.23	G1_S
A_32_P318086	*FLAD1*	NM_025207	80308	−0.63	−0.30	0.33	−0.11	G1_S
A_32_P6917	*FLAD1*	NM_025207	80308	−0.68	−0.18	0.50	0.02	G1_S
A_23_P34527	*FLAD1*	NM_025207	80308	−0.67	−0.05	0.62	−0.18	G1_S
A_23_P118246	*GINS2*	NM_016095	51659	−3.12	−2.76	0.36	−1.06	G1_S
A_23_P152136	*GINS3*	NM_022770	64785	−0.67	−0.65	0.02	0.09	G1_S
A_24_P159323	*GINS3*	NM_022770	64785	0.12	0.26	0.14	−0.05	G1_S
A_23_P19712	*GMNN*	NM_015895	51053	−0.77	−0.85	−0.08	0.45	G1_S
A_23_P99579	*GON7*	NM_032490	84520	−0.62	−0.44	0.18	0.05	G1_S
A_24_P567952	*HCG18*	NR_024052	414777	−0.54	−0.06	0.48	−0.46	G1_S
A_24_P934162	*HCG18*	A_24_P934162	NA	0.28	−0.15	−0.43	0.48	G1_S
A_32_P181722	*HCG18*	NR_024052	414777	−0.15	0.07	0.22	−0.19	G1_S
A_24_P567944	*HCG18*	NR_024052	414777	−0.07	0.23	0.30	−0.14	G1_S
A_32_P199884	*HORMAD1*	NM_032132	84072	0.28	0.03	−0.25	0.24	G1_S
A_24_P416370	*HOXB4*	NM_024015	3214	−0.84	−0.78	0.06	−0.48	G1_S
A_24_P305067	*HOXB4*	NM_024015	3214	−0.09	0.18	0.27	−0.62	G1_S
A_23_P98183	*HRAS*	NM_005343	3265	0.32	0.49	0.17	−0.12	G1_S
A_32_P9963	*HSF2*	NM_004506	3298	0.28	−0.09	−0.37	0.80	G1_S
A_23_P111360	*HSF2*	NM_004506	3298	0.21	−0.23	−0.44	0.62	G1_S
A_23_P43079	*INTS8*	NM_017864	55656	0.04	0.25	0.21	0.64	G1_S
A_23_P391506	*IVNS1ABP*	NM_006469	10625	−0.60	−0.45	0.15	−0.49	G1_S
A_23_P137514	*IVNS1ABP*	NM_006469	10625	−0.36	−0.18	0.18	0.63	G1_S
A_24_P324787	*KANK2*	NM_015493	25959	−0.33	0.05	0.38	−0.90	G1_S
A_23_P50426	*KANK2*	NM_015493	25959	−0.35	0.16	0.51	−1.00	G1_S
A_23_P55897	*KANK2*	NM_015493	25959	−0.05	0.24	0.29	−0.50	G1_S
A_23_P12079	*KCNC4*	NM_153763	3749	−0.37	−0.04	0.33	−0.43	G1_S
A_23_P404821	*KIAA1147*	NM_001080392	57189	−0.25	0.09	0.34	−0.52	G1_S
A_24_P101047	*KIAA1586*	NM_020931	57691	−0.24	0.03	0.27	−0.25	G1_S
A_24_P230965	*KIAA1586*	NM_020931	57691	−0.20	−0.22	−0.02	0.24	G1_S
A_24_P237559	*LNPEP*	AK096804	4012	0.41	0.10	−0.31	0.64	G1_S
A_23_P144677	*LNPEP*	ENST00000231368	4012	−0.29	−0.02	0.27	−0.56	G1_S
A_24_P132019	*LNPEP*	ENST00000231368	4012	0.21	0.36	0.15	−0.16	G1_S
A_23_P156061	*LNPEP*	NM_005575	4012	−0.09	−0.07	0.02	0.24	G1_S
A_32_P69475	*LNPEP*	ENST00000231368	4012	0.03	0.05	0.02	−0.30	G1_S
A_23_P207445	*MAP2K6*	NM_002758	5608	−2.10	−0.61	1.49	−1.44	G1_S
A_23_P408996	*MBOAT1*	NM_001080480	154141	−0.45	−0.03	0.42	−0.57	G1_S
A_32_P103633	*MCM2*	NM_004526	4171	−1.61	−1.56	0.06	−1.32	G1_S
A_23_P132277	*MCM5*	NM_006739	4174	−1.85	−1.65	0.20	−1.36	G1_S
A_23_P90612	*MCM6*	NM_005915	4175	−1.27	−1.79	−0.52	0.06	G1_S
A_23_P204782	*MDM1*	NM_020128	56890	−0.85	−0.55	0.29	−0.15	G1_S
A_23_P413180	*MDM1*	NM_017440	56890	−0.66	−0.11	0.55	−0.69	G1_S
A_23_P105730	*MDM1*	NM_020128	56890	−0.14	−0.02	0.12	0.24	G1_S
A_24_P313678	*MED31*	NM_016060	51003	0.18	0.08	−0.11	0.78	G1_S
A_23_P341443	*MNT*	NM_020310	4335	0.68	0.66	−0.02	−0.28	G1_S
A_24_P350969	*MNT*	AF318360	4335	0.08	−0.20	−0.28	0.56	G1_S
A_32_P6015	*MNX1*	NM_005515	3110	−0.33	−0.42	−0.10	−0.36	G1_S
A_23_P253331	*MNX1*	NM_005515	3110	−0.07	−0.33	−0.26	0.05	G1_S
A_24_P279797	*MRI1*	NM_001031727	84245	0.01	0.11	0.10	0.38	G1_S
A_23_P102471	*MSH2*	NM_000251	4436	−0.38	−0.39	−0.02	−0.28	G1_S
A_23_P34800	*NASP*	NM_172164	4678	−0.92	−0.57	0.34	−0.26	G1_S
A_32_P28365	*NASP*	NM_172164	4678	−0.70	−0.46	0.24	−0.15	G1_S
A_24_P926760	*NKTR*	NM_005385	4820	0.53	0.36	−0.17	0.78	G1_S
A_23_P212002	*NKTR*	NM_005385	4820	0.38	0.32	−0.06	0.47	G1_S
A_24_P171601	*NKTR*	NM_005385	4820	0.37	0.20	−0.16	0.83	G1_S
A_23_P203013	*NPAT*	NM_002519	4863	−0.59	−0.36	0.23	−0.18	G1_S
A_24_P273823	*NPAT*	NM_002519	4863	−0.22	−0.31	−0.09	0.27	G1_S
A_24_P29641	*NSUN5P2*	NM_148936	260294	0.05	0.12	0.07	0.46	G1_S
A_23_P161324	*NUDT13*	NM_015901	25961	−0.39	−0.32	0.07	−0.01	G1_S
A_32_P41471	*NUDT13*	NM_015901	25961	−0.19	−0.31	−0.11	0.29	G1_S
A_24_P200761	*NUP43*	NM_198887	348995	−0.37	−0.10	0.27	−0.24	G1_S
A_23_P31055	*NUP43*	NM_198887	348995	−0.21	0.02	0.22	0.16	G1_S
A_23_P45799	*ORC1*	NM_004153	4998	−0.91	−0.95	−0.04	−0.48	G1_S
A_24_P371053	*ORMDL1*	NM_016467	94101	−0.59	−0.20	0.39	−0.10	G1_S
A_23_P120194	*ORMDL1*	NM_016467	94101	−0.01	0.07	0.08	0.25	G1_S
A_32_P220762	*OSBPL6*	ENST00000190611	114880	0.10	−0.19	−0.29	0.11	G1_S
A_23_P108823	*OSBPL6*	NM_032523	114880	0.00	−0.27	−0.28	0.17	G1_S
A_24_P414446	*OTULIN*	NM_138348	90268	−0.16	−0.55	−0.39	0.09	G1_S
A_23_P353106	*OTULIN*	NM_138348	90268	0.13	−0.25	−0.38	0.12	G1_S
A_24_P142885	*PANK2*	ENST00000497424	80025	−0.56	−0.16	0.41	−0.45	G1_S
A_23_P79942	*PANK2*	NM_153638	80025	−0.12	0.06	0.17	−0.25	G1_S
A_24_P299911	*PASK*	NM_015148	23178	−0.45	−0.28	0.18	−0.39	G1_S
A_23_P28886	*PCNA*	NM_002592	5111	−1.10	−0.88	0.22	−0.11	G1_S
A_24_P280029	*PDXP*	NM_020315	57026	0.09	−0.20	−0.28	0.81	G1_S
A_23_P61180	*PLCXD1*	NM_018390	55344	0.64	0.69	0.06	0.14	G1_S
A_23_P99582	*PNN*	NM_002687	5411	0.46	0.30	−0.16	1.13	G1_S
A_32_P182439	*POLD3*	NM_006591	10714	−0.43	−0.01	0.42	−0.26	G1_S
A_24_P75056	*POLD3*	NM_006591	10714	−0.26	0.19	0.45	−0.45	G1_S
A_23_P70794	*RAB23*	NM_016277	51715	−0.48	−0.19	0.29	−0.27	G1_S
A_23_P71558	*RECQL4*	NM_004260	9401	−1.56	−0.91	0.65	−0.90	G1_S
A_23_P353717	*RMI2*	NM_152308	116028	−1.49	−1.51	−0.02	−1.39	G1_S
A_23_P258071	*RNF113A*	NM_006978	7737	0.21	−0.05	−0.26	0.49	G1_S
A_23_P254970	*RNPC3*	AK057799	55599	−0.24	−0.35	−0.11	0.31	G1_S
A_24_P341504	*RNPC3*	NM_017619	55599	0.23	0.13	−0.10	0.71	G1_S
A_23_P34396	*RSRP1*	NM_020317	57035	0.66	0.09	−0.57	0.97	G1_S
A_24_P34155	*RUNX1*	NM_001122607	861	1.03	0.33	−0.71	0.67	G1_S
A_24_P96403	*RUNX1*	NM_001001890	861	−0.68	0.18	0.87	−1.35	G1_S
A_23_P16944	*SDC1*	NM_001006946	6382	−0.69	−0.50	0.19	−1.07	G1_S
A_24_P97129	*SDC1*	NM_001006946	6382	−0.35	−0.44	−0.08	0.50	G1_S
A_23_P357856	*SEC62*	NM_003262	7095	−0.56	−0.28	0.28	−0.71	G1_S
A_23_P144224	*SEC62*	NM_003262	7095	0.54	0.07	−0.47	0.48	G1_S
A_24_P285880	*SEC62*	NM_003262	7095	−0.35	−0.26	0.10	0.05	G1_S
A_23_P357860	*SEC62*	NM_003262	7095	−0.52	0.04	0.56	−1.67	G1_S
A_24_P251704	*SEC62*	NM_003262	7095	−0.05	0.04	0.09	−0.96	G1_S
A_23_P55632	*SERPINB3*	NM_006919	6317	1.00	1.78	0.78	1.52	G1_S
A_23_P156310	*SKP2*	NM_032637	6502	−0.38	−0.75	−0.37	0.37	G1_S
A_23_P7101	*SLBP*	NM_006527	7884	0.21	0.00	−0.21	0.23	G1_S
A_23_P40896	*SLC25A36*	NM_018155	55186	0.27	−0.15	−0.42	0.91	G1_S
A_23_P408455	*SLC25A36*	NM_001104647	55186	0.24	−0.37	−0.61	0.85	G1_S
A_24_P136725	*SPIN3*	NR_027139	169981	−0.07	0.34	0.40	0.01	G1_S
A_24_P494454	*SPIN3*	NM_001010862	169981	0.01	0.06	0.05	1.15	G1_S
A_32_P222961	*SPIN4*	NM_001012968	139886	0.33	0.38	0.06	0.30	G1_S
A_24_P467371	*SPIN4*	NM_001012968	139886	0.29	0.10	−0.19	−0.42	G1_S
A_24_P222911	*SRSF7*	NM_001031684	6432	−0.81	−0.73	0.08	−0.35	G1_S
A_23_P39704	*SRSF7*	NM_001031684	6432	−0.58	−0.60	−0.02	−0.01	G1_S
A_23_P155229	*SSR3*	NM_007107	6747	0.53	−0.06	−0.58	0.94	G1_S
A_24_P319942	*SSR3*	NM_007107	6747	−0.06	−0.23	−0.18	−0.01	G1_S
A_24_P928068	*TAF15*	DB509819	NA	0.46	0.45	0.00	0.02	G1_S
A_23_P159305	*TAF15*	NM_139215	8148	0.11	0.26	0.15	−0.66	G1_S
A_32_P56525	*TCAF1*	NM_014719	9747	0.28	0.46	0.18	−1.13	G1_S
A_24_P380628	*TCAF1*	NM_014719	9747	−0.08	0.10	0.18	−0.69	G1_S
A_24_P368023	*TCAF1*	ENST00000479870	9747	−0.03	0.26	0.29	−1.42	G1_S
A_23_P99930	*TIPIN*	NM_017858	54962	0.17	−0.46	−0.63	0.86	G1_S
A_23_P157283	*TMEM243*	NM_024315	79161	0.17	0.30	0.14	−0.37	G1_S
A_23_P159390	*TOPBP1*	NM_007027	11073	−0.46	−0.26	0.21	−0.01	G1_S
A_23_P31389	*TRA2A*	NM_013293	29896	−0.67	−0.32	0.34	−0.04	G1_S
A_23_P218879	*TREX1*	NM_016381	11277	−0.44	−0.10	0.34	−0.38	G1_S
A_24_P339858	*TSPEAR-AS2*	NR_026547	114043	0.36	0.72	0.36	−0.13	G1_S
A_24_P910854	*TTC14*	NM_001042601	151613	0.51	0.21	−0.30	−0.10	G1_S
A_23_P212511	*TTC14*	NM_001042601	151613	−0.10	−0.25	−0.14	0.52	G1_S
A_24_P159094	*UBR7*	NM_175748	55148	−0.97	−0.65	0.33	−0.45	G1_S
A_23_P205393	*UBR7*	NM_175748	55148	−0.43	−0.63	−0.21	0.41	G1_S
A_23_P208880	*UHRF1*	NM_013282	29128	−2.52	−1.75	0.77	−1.77	G1_S
A_32_P101235	*UHRF1*	NM_013282	29128	−0.47	−0.43	0.04	−0.15	G1_S
A_24_P398585	*UNG*	NM_003362	7374	−0.28	0.01	0.29	−0.27	G1_S
A_24_P137522	*USP53*	NM_019050	54532	0.46	0.26	−0.20	0.60	G1_S
A_32_P128701	*USP53*	NM_019050	54532	0.40	0.15	−0.25	0.38	G1_S
A_23_P115215	*VPS72*	NM_005997	6944	−0.35	−0.10	0.25	0.01	G1_S
A_23_P129075	*WDR76*	NM_024908	79968	−0.19	−0.48	−0.29	−0.09	G1_S
A_24_P158385	*ZMYND19*	NM_138462	116225	−0.29	−0.28	0.01	−0.38	G1_S
A_32_P183218	*ZNF367*	NM_153695	195828	−1.12	−0.76	0.35	−0.53	G1_S
A_23_P410625	*ZNF367*	NM_153695	195828	−0.41	−0.50	−0.09	−0.08	G1_S
A_23_P340922	*ZNF414*	NM_032370	84330	−0.35	0.32	0.68	−0.93	G1_S
A_32_P85978	*ZNF414*	NM_001146175	84330	0.21	0.26	0.05	−0.48	G1_S
A_23_P85521	*ZRANB2*	NM_203350	9406	0.36	0.09	−0.27	0.81	G1_S
A_24_P242299	*ZRANB2*	NM_005455	9406	0.30	0.10	−0.20	0.57	G1_S
A_23_P120784	*TRMT2A*	NM_022727	27037	−0.61	−0.27	0.34	−0.58	G1_S,G2
A_24_P305662	*TRMT2A*	NM_022727	27037	−0.33	−0.12	0.21	−0.20	G1_S,G2
A_23_P370989	*MCM4*	NM_005914	4173	−1.44	−1.07	0.36	−0.90	G1_S,G2_M
A_24_P59596	*ATAD2*	NM_014109	29028	−1.00	−1.55	−0.55	0.22	G1_S,S
A_23_P216068	*ATAD2*	NM_014109	29028	−0.82	−0.97	−0.15	−0.21	G1_S,S
A_23_P387943	*CASP2*	NM_032982	835	−0.26	0.15	0.41	−0.75	G1_S,S
A_24_P269398	*CASP2*	NM_032982	835	−0.17	0.04	0.21	−0.43	G1_S,S
A_23_P215701	*CASP2*	NM_032982	835	−0.13	0.18	0.31	−0.40	G1_S,S
A_23_P203645	*CREBZF*	NM_001039618	58487	0.69	0.06	−0.63	0.90	G1_S,S
A_23_P252740	*DSCC1*	NM_024094	79075	−1.22	−1.25	−0.04	−0.64	G1_S,S
A_23_P162579	*HSPB8*	NM_014365	26353	1.38	0.02	−1.35	1.06	G1_S,S
A_23_P170110	*NEAT1*	AW806882	NA	0.73	0.39	−0.34	1.00	G1_S,S
A_24_P290999	*NEAT1*	NR_028272	283131	0.77	−0.15	−0.92	1.04	G1_S,S
A_24_P566916	*NEAT1*	NR_028272	283131	0.38	0.03	−0.34	0.03	G1_S,S
A_23_P160518	*TRIM45*	NM_025188	80263	−0.40	−0.18	0.22	−0.16	G1_S,S
A_23_P160523	*TRIM45*	NM_025188	80263	−0.22	−0.20	0.02	−0.47	G1_S,S
A_23_P14543	*ALKBH1*	NM_006020	8846	0.21	0.05	−0.16	0.40	G2
A_23_P108135	*AP3D1*	NM_003938	8943	0.58	0.63	0.05	−0.50	G2
A_23_P119526	*AP3D1*	NM_003938	8943	0.46	0.51	0.05	−0.18	G2
A_24_P30034	*ARHGEF39*	NM_032818	84904	−0.48	−0.49	−0.01	0.19	G2
A_23_P216517	*ARHGEF39*	NM_032818	84904	−0.48	−0.91	−0.43	0.45	G2
A_32_P234827	*ARMC1*	NM_018120	55156	0.00	−0.04	−0.05	0.23	G2
A_23_P415015	*ATL2*	NM_022374	64225	0.40	0.35	−0.05	0.17	G2
A_23_P209619	*ATL2*	NM_022374	64225	0.07	0.40	0.33	−0.17	G2
A_23_P130182	*AURKB*	NM_004217	9212	−1.55	−1.32	0.24	−0.60	G2
A_24_P89512	*BCLAF1*	NM_014739	9774	−0.94	−0.52	0.43	−0.39	G2
A_24_P80915	*BCLAF1*	NM_014739	9774	−0.85	−0.89	−0.04	−0.03	G2
A_23_P111343	*BCLAF1*	NM_014739	9774	−0.56	−0.34	0.22	0.35	G2
A_23_P25626	*BORA*	NM_024808	79866	−1.02	−0.89	0.12	0.28	G2
A_23_P145016	*BRD8*	NM_006696	10902	−0.13	0.02	0.15	−0.28	G2
A_23_P81280	*BTNL9*	NM_152547	153579	0.42	0.29	−0.14	0.18	G2
A_32_P187951	*BTNL9*	NM_152547	153579	−0.29	−0.38	−0.09	−0.48	G2
A_23_P46924	*BUB3*	NM_001007793	9184	−0.66	−0.53	0.13	−0.08	G2
A_23_P202316	*BUB3*	NM_001007793	9184	−0.63	−0.34	0.29	−0.25	G2
A_23_P320658	*BUB3*	NM_004725	9184	0.20	−0.10	−0.29	0.52	G2
A_24_P413941	*C2orf69*	NM_153689	205327	−0.24	−0.07	0.17	−0.37	G2
A_23_P142918	*C2orf69*	NM_153689	205327	0.10	0.06	−0.04	−0.04	G2
A_23_P92410	*CASP3*	NM_004346	836	−0.51	−0.13	0.38	−0.08	G2
A_24_P664995	*CBX5*	NM_001127322	23468	−0.34	0.24	0.57	−1.23	G2
A_24_P620621	*CBX5*	NM_001127322	23468	−0.15	−0.02	0.13	−0.14	G2
A_23_P2355	*CBX5*	NM_012117	23468	−0.17	−0.29	−0.12	0.49	G2
A_24_P193592	*CCNF*	NM_001761	899	−0.89	−0.41	0.48	−0.46	G2
A_23_P37954	*CCNF*	NM_001761	899	−0.74	−0.22	0.52	−0.94	G2
A_23_P88083	*CDC16*	NM_003903	8881	−0.36	−0.13	0.23	0.02	G2
A_23_P70249	*CDC25C*	NM_001790	995	−2.28	−1.87	0.41	−0.78	G2
A_23_P385861	*CDCA2*	NM_152562	157313	−1.95	−1.77	0.18	−0.24	G2
A_24_P323434	*CDCA2*	NM_152562	157313	−1.36	−1.32	0.04	−0.07	G2
A_23_P375	*CDCA8*	NM_018101	55143	−2.04	−1.76	0.29	−0.84	G2
A_23_P138507	*CDK1*	NM_001786	983	−2.95	−2.22	0.73	−0.84	G2
A_24_P282343	*CDKL5*	NM_003159	6792	0.14	−0.06	−0.20	−0.28	G2
A_24_P81841	*CDKN1B*	NM_004064	1027	−0.70	−0.55	0.15	−0.73	G2
A_23_P204696	*CDKN1B*	NM_004064	1027	−0.41	−0.54	−0.13	0.24	G2
A_23_P85460	*CDKN2C*	NM_078626	1031	−0.61	−1.44	−0.82	−0.04	G2
A_23_P126120	*CENPL*	NM_033319	91687	−0.75	−0.56	0.19	−0.24	G2
A_24_P930100	*CENPL*	AK056348	91687	0.01	−0.68	−0.69	0.61	G2
A_23_P201816	*CEP350*	NM_014810	9857	−0.25	−0.13	0.12	−0.12	G2
A_23_P119562	*CFD*	NM_001928	1675	−0.36	−0.22	0.13	−0.26	G2
A_23_P109452	*CHEK2*	NM_001005735	11200	−0.96	−0.67	0.28	0.01	G2
A_23_P250313	*CIP2A*	NM_020890	57650	−0.85	−0.77	0.08	0.01	G2
A_24_P351466	*CIP2A*	NM_020890	57650	0.02	−0.40	−0.42	0.14	G2
A_23_P388812	*CKAP2L*	NM_152515	150468	−2.34	−2.03	0.31	−0.35	G2
A_23_P213745	*CXCL14*	NM_004887	9547	−2.26	−1.16	1.10	−2.35	G2
A_23_P2181	*CYB5R2*	NM_016229	51700	0.31	−0.19	−0.49	1.01	G2
A_23_P119377	*CYTH2*	NM_004228	9266	−0.42	−0.11	0.31	−0.64	G2
A_23_P422268	*DCAF7*	NM_005828	10238	0.32	0.42	0.10	−0.21	G2
A_24_P916141	*DCAF7*	NM_005828	10238	−0.20	0.23	0.44	−1.47	G2
A_24_P91222	*DCAF7*	NM_005828	10238	0.13	0.10	−0.02	−0.15	G2
A_23_P26836	*DCAF7*	NM_005828	10238	0.10	0.41	0.31	−1.19	G2
A_32_P430743	*DET1*	AK125793	NA	1.07	0.74	−0.33	0.27	G2
A_23_P26184	*DET1*	NM_017996	55070	0.04	0.07	0.03	0.09	G2
A_23_P124224	*DHX8*	NM_004941	1659	0.03	0.04	0.02	0.16	G2
A_23_P119478	*EBI3*	NM_005755	10148	4.87	4.24	−0.63	4.69	G2
A_24_P370201	*EBI3*	NM_005755	10148	1.35	0.70	−0.65	1.40	G2
A_23_P117580	*ENTPD5*	NM_001249	957	0.12	0.09	−0.03	0.16	G2
A_23_P32707	*ESPL1*	NM_012291	9700	−0.40	−1.11	−0.70	0.46	G2
A_32_P119007	*ESPL1*	NM_012291	9700	0.48	1.80	1.33	−0.18	G2
A_24_P278637	*FADD*	NM_003824	8772	−0.41	−0.08	0.33	−0.18	G2
A_23_P86917	*FADD*	NM_003824	8772	−0.33	0.05	0.37	−0.32	G2
A_23_P386241	*FAM110A*	NM_001042353	83541	0.01	0.38	0.37	−0.66	G2
A_23_P323751	*FAM83D*	NM_030919	81610	−1.23	−1.77	−0.54	0.09	G2
A_23_P377888	*FAN1*	NM_014967	22909	0.24	0.15	−0.09	0.33	G2
A_23_P345678	*FANCD2*	NM_033084	2177	−0.61	−1.11	−0.50	−0.17	G2
A_32_P24165	*FANCD2*	NM_001018115	2177	−0.59	−0.68	−0.09	−0.10	G2
A_23_P143994	*FANCD2*	NM_001018115	2177	−0.59	−0.56	0.03	−0.90	G2
A_23_P142333	*FZR1*	NM_016263	51343	0.40	0.18	−0.22	0.12	G2
A_23_P142325	*FZR1*	NM_001136198	51343	0.41	0.05	−0.35	0.11	G2
A_24_P944291	*FZR1*	ENST00000395095	51343	0.04	0.25	0.21	−0.49	G2
A_24_P318836	*FZR1*	NM_016263	51343	−0.04	0.02	0.06	−0.53	G2
A_23_P106280	*GABPB1*	NR_026891	55056	−0.85	−0.42	0.43	−0.95	G2
A_23_P205789	*GABPB1*	NM_002041	2553	1.27	0.43	−0.84	1.47	G2
A_24_P176255	*GABPB1*	NM_005254	2553	−0.14	−0.02	0.12	0.16	G2
A_23_P83134	*GAS1*	NM_002048	2619	0.50	−0.71	−1.21	0.26	G2
A_24_P38895	*H2AFX*	NM_002105	3014	−1.09	−0.78	0.31	−0.88	G2
A_23_P141965	*HAUS8*	NM_033417	93323	−0.41	−0.45	−0.04	0.18	G2
A_32_P85500	*HCP5*	NM_006674	10866	0.72	0.39	−0.32	0.76	G2
A_23_P111126	*HCP5*	L06175	10866	0.55	0.24	−0.31	0.80	G2
A_24_P17870	*HCP5*	NM_006674	10866	0.52	0.14	−0.37	0.70	G2
A_24_P238609	*HCP5*	NM_006674	10866	0.28	0.09	−0.19	0.40	G2
A_23_P145574	*HINT3*	NM_138571	135114	0.38	0.18	−0.20	0.80	G2
A_24_P681011	*HIPK2*	NM_022740	28996	0.20	0.72	0.52	−2.31	G2
A_23_P169756	*HIPK2*	NM_022740	28996	0.20	0.39	0.19	−1.41	G2
A_24_P500621	*HIPK2*	NM_022740	28996	0.10	0.39	0.29	−1.71	G2
A_23_P169766	*HIPK2*	NM_022740	28996	−0.02	0.20	0.22	−1.50	G2
A_23_P149301	*HIST3H2A*	NM_033445	92815	0.15	0.07	−0.08	0.79	G2
A_24_P257099	*HJURP*	NM_018410	55355	−2.18	−1.32	0.86	−1.01	G2
A_23_P155765	*HMGB2*	NM_002129	3148	−0.77	−2.05	−1.29	0.90	G2
A_23_P88303	*HSPA2*	NM_021979	3306	1.00	0.29	−0.71	0.23	G2
A_23_P17633	*IFNAR1*	NM_000629	3454	0.54	0.11	−0.43	0.40	G2
A_23_P113803	*KATNA1*	NM_007044	11104	0.17	−0.33	−0.50	0.62	G2
A_23_P77286	*KATNBL1*	NM_024713	79768	−0.55	−0.37	0.18	−0.19	G2
A_32_P58163	*KATNBL1*	NM_024713	79768	−0.34	−0.15	0.20	−0.11	G2
A_24_P12539	*KBTBD2*	NM_015483	25948	0.20	−0.15	−0.35	0.37	G2
A_23_P70951	*KBTBD2*	NM_015483	25948	0.06	0.00	−0.07	0.06	G2
A_23_P74446	*KDM4A*	NM_014663	9682	−0.20	0.07	0.27	−0.48	G2
A_24_P227091	*KIF11*	NM_004523	3832	−2.63	−1.71	0.93	−1.86	G2
A_23_P52278	*KIF11*	NM_004523	3832	−1.24	−0.93	0.31	−0.76	G2
A_23_P54622	*KIF22*	NM_007317	3835	−1.46	−1.13	0.33	−0.79	G2
A_23_P133956	*KIFC1*	NM_002263	3833	−2.20	−1.79	0.41	−1.11	G2
A_24_P252739	*KLF6*	NM_001300	1316	−1.45	−1.02	0.43	−0.96	G2
A_24_P932981	*KLF6*	NM_001300	1316	0.59	−0.18	−0.77	−0.30	G2
A_24_P69654	*KLF6*	NM_001300	1316	0.81	−0.29	−1.10	0.00	G2
A_23_P63798	*KLF6*	NM_001300	1316	0.57	−0.29	−0.86	−0.26	G2
A_23_P125265	*KPNA2*	NM_002266	3838	−1.25	−0.63	0.62	−0.25	G2
A_23_P342744	*LIX1L*	NM_153713	128077	0.56	0.04	−0.52	0.40	G2
A_24_P687594	*LIX1L*	ENST00000369308	128077	0.30	−0.02	−0.31	−0.45	G2
A_23_P258493	*LMNB1*	NM_005573	4001	−1.90	−1.59	0.31	−1.26	G2
A_24_P264790	*LTBP3*	NM_021070	4054	−0.85	−0.57	0.28	−0.36	G2
A_24_P298360	*LTBP3*	NM_021070	4054	0.36	−0.16	−0.51	−0.64	G2
A_23_P92441	*MAD2L1*	NM_002358	4085	−1.86	−1.58	0.28	−0.36	G2
A_24_P873659	*MALAT1*	NR_002819	378938	0.29	−0.40	−0.69	1.76	G2
A_23_P21143	*MALAT1*	NR_002819	378938	−0.19	−0.44	−0.25	0.80	G2
A_24_P829261	*MALAT1*	NR_002819	378938	0.11	0.03	−0.08	0.27	G2
A_24_P497244	*MALAT1*	NR_002819	378938	0.07	−0.32	−0.40	1.05	G2
A_23_P94422	*MELK*	NM_014791	9833	−3.11	−2.07	1.04	−1.29	G2
A_23_P42626	*MEPCE*	NM_019606	56257	−0.18	0.15	0.32	−0.74	G2
A_24_P312189	*MEPCE*	NM_019606	56257	−0.16	0.24	0.40	−0.71	G2
A_23_P145844	*MET*	NM_000245	4233	0.51	0.41	−0.10	0.86	G2
A_23_P145846	*MET*	NM_000245	4233	0.27	0.27	0.00	0.71	G2
A_23_P359245	*MET*	NM_000245	4233	0.22	0.30	0.08	0.11	G2
A_23_P65558	*MGAT2*	NM_002408	4247	−0.19	−0.39	−0.20	1.27	G2
A_32_P128656	*MID1*	NM_000381	4281	−0.31	0.14	0.46	−0.59	G2
A_23_P170037	*MID1*	NM_033290	4281	−0.01	0.50	0.50	−0.87	G2
A_23_P133123	*MND1*	NM_032117	84057	−2.67	−2.06	0.61	−1.08	G2
A_23_P360605	*MTCL1*	NM_015210	23255	−1.12	−0.38	0.74	−1.41	G2
A_23_P137856	*MUC1*	NM_002456	4582	−0.29	0.17	0.46	−1.61	G2
A_32_P71447	*NCAPD3*	NM_015261	23310	−0.92	−0.59	0.32	−0.53	G2
A_23_P415443	*NCAPH*	NM_015341	23397	−2.82	−2.15	0.67	−1.02	G2
A_23_P50108	*NDC80*	NM_006101	10403	−3.12	−2.49	0.64	−0.69	G2
A_24_P14156	*NDC80*	NM_006101	10403	−1.90	−1.59	0.31	−0.41	G2
A_23_P155711	*NEIL3*	NM_018248	55247	−0.52	−0.51	0.02	0.19	G2
A_24_P356830	*NFIC*	AK129956	4782	0.20	0.15	−0.06	−0.83	G2
A_23_P131115	*NFIC*	NM_005597	4782	−0.18	0.31	0.49	−0.92	G2
A_24_P180383	*NIPBL*	NM_015384	25836	−0.12	0.22	0.33	−0.46	G2
A_23_P213883	*NIPBL*	NM_133433	25836	0.09	−0.04	−0.13	−0.15	G2
A_24_P357688	*NIPBL*	NM_015384	25836	0.10	−0.05	−0.16	0.70	G2
A_24_P213161	*NLRP2*	NM_017852	55655	−0.78	−0.17	0.61	−1.33	G2
A_23_P88522	*NMB*	NM_021077	4828	1.35	0.91	−0.44	2.12	G2
A_23_P127584	*NNMT*	NM_006169	4837	−0.01	0.25	0.27	−0.30	G2
A_24_P787914	*NR3C1*	U25029	2908	1.13	0.58	−0.55	1.02	G2
A_23_P214059	*NR3C1*	NM_001018077	2908	0.83	0.19	−0.64	0.56	G2
A_24_P214754	*NR3C1*	NM_001018077	2908	0.80	0.04	−0.76	1.23	G2
A_24_P216968	*NUCKS1*	NM_022731	64710	−0.40	−0.39	0.01	−0.04	G2
A_24_P145122	*NUCKS1*	NM_022731	64710	−0.35	−0.36	−0.01	−0.07	G2
A_24_P216964	*NUCKS1*	NM_022731	64710	−0.23	−0.48	−0.25	−0.15	G2
A_24_P374652	*NUCKS1*	NM_022731	64710	0.09	−0.04	−0.14	0.21	G2
A_23_P149724	*NUCKS1*	NM_022731	64710	−0.02	−0.24	−0.22	0.07	G2
A_23_P162120	*NUMA1*	NM_006185	4926	0.11	0.39	0.28	−0.38	G2
A_23_P17471	*PCED1A*	NM_022760	64773	−0.13	−0.27	−0.14	0.08	G2
A_23_P416468	*PIF1*	NM_025049	80119	−2.16	−1.63	0.53	−0.08	G2
A_23_P323749	*PIF1*	NM_025049	80119	−0.37	0.19	0.56	−0.14	G2
A_23_P323743	*PIF1*	NM_025049	80119	−0.19	−0.27	−0.08	0.04	G2
A_24_P196534	*PKNOX1*	NM_004571	5316	0.32	0.19	−0.14	0.10	G2
A_23_P211299	*PKNOX1*	NM_004571	5316	0.20	0.25	0.05	0.49	G2
A_24_P378907	*PKNOX1*	NM_004571	5316	−0.23	0.25	0.48	−0.15	G2
A_23_P333998	*POLQ*	AF090919	10721	−1.90	−1.42	0.48	−1.15	G2
A_23_P218827	*POLQ*	NM_199420	10721	−2.19	−1.69	0.49	−1.00	G2
A_24_P63109	*PPP1R2*	NM_006241	5504	0.30	−0.18	−0.48	0.89	G2
A_32_P17133	*PPP1R2*	NM_006241	5504	0.11	−0.21	−0.31	0.63	G2
A_24_P174367	*PPP1R2*	NM_006241	5504	−0.06	−0.17	−0.11	0.34	G2
A_23_P46539	*PSRC1*	NM_032636	84722	−0.92	−0.66	0.26	−0.48	G2
A_23_P106439	*RCCD1*	NM_033544	91433	−0.52	−0.10	0.41	−0.66	G2
A_23_P106433	*RCCD1*	NM_033544	91433	−0.23	−0.05	0.17	−0.62	G2
A_23_P25684	*RDH11*	NM_016026	51109	−0.39	−0.19	0.20	−0.56	G2
A_24_P377775	*RGS3*	NM_017790	5998	0.85	0.37	−0.48	0.24	G2
A_23_P219197	*RGS3*	NM_134427	5998	−0.03	−0.04	−0.01	−0.43	G2
A_23_P134714	*RIDA*	NM_005836	10247	−0.54	−0.12	0.42	−0.41	G2
A_23_P121602	*SAP30*	NM_003864	8819	−0.39	−0.22	0.16	0.11	G2
A_23_P147647	*SGCD*	NM_000337	6444	0.23	−0.19	−0.41	0.51	G2
A_23_P136254	*SGCD*	NM_172244	6444	0.17	0.37	0.20	−0.63	G2
A_32_P4595	*SGCD*	NM_000337	6444	0.05	−0.38	−0.43	0.33	G2
A_23_P340909	*SKA3*	BC013418	221150	−3.76	−2.71	1.05	−1.64	G2
A_23_P327643	*SMC4*	A_23_P327643	NA	0.61	0.07	−0.53	0.40	G2
A_23_P91900	*SMC4*	NM_005496	10051	−0.46	−0.37	0.09	−0.62	G2
A_23_P87049	*SORL1*	NM_003105	6653	0.07	0.31	0.24	−0.86	G2
A_23_P56630	*STAT1*	NM_007315	6772	0.35	0.58	0.23	−0.36	G2
A_24_P274270	*STAT1*	NM_139266	6772	0.16	0.43	0.28	−0.09	G2
A_24_P214231	*STIL*	NM_001048166	6491	−0.73	0.04	0.78	−0.96	G2
A_23_P154367	*STK17B*	NM_004226	9262	−0.33	−0.57	−0.24	0.27	G2
A_24_P636882	*STK17B*	NM_004226	9262	−0.30	0.07	0.37	−0.78	G2
A_23_P100022	*SV2B*	NM_014848	9899	−0.25	0.02	0.27	0.33	G2
A_23_P431381	*TEDC1*	NM_001134875	283643	−0.52	−0.16	0.37	−0.24	G2
A_32_P14187	*TFAP2A*	NM_001032280	7020	0.71	0.16	−0.56	0.26	G2
A_23_P62115	*TIMP1*	NM_003254	7076	0.09	0.06	−0.03	0.27	G2
A_23_P24716	*TMEM132A*	NM_017870	54972	0.30	0.29	0.00	−0.74	G2
A_24_P210244	*TMPO*	NM_001032283	7112	−0.85	−0.65	0.20	0.42	G2
A_23_P325040	*TMPO*	NM_003276	7112	−0.33	−0.59	−0.26	−0.17	G2
A_24_P44891	*TNPO2*	NM_013433	30000	0.63	0.13	−0.50	1.02	G2
A_23_P354953	*TNPO2*	NM_013433	30000	−0.43	0.05	0.48	−0.11	G2
A_23_P170491	*TRAIP*	NM_005879	10293	−0.48	−0.61	−0.12	0.31	G2
A_23_P407718	*TRIM59*	NM_173084	286827	−0.89	−0.76	0.13	−0.13	G2
A_32_P72341	*TRIM59*	NM_173084	286827	−0.44	−0.58	−0.14	0.05	G2
A_23_P48826	*TRIM69*	NM_182985	140691	0.42	0.05	−0.38	0.44	G2
A_24_P50543	*TRIM69*	A_24_P50543	NA	−0.04	−0.01	0.03	0.21	G2
A_23_P254193	*TTC38*	NM_017931	55020	−0.45	0.19	0.64	−0.76	G2
A_24_P156388	*TTC38*	NM_017931	55020	−0.35	0.01	0.36	−0.53	G2
A_32_P168388	*TTF2*	AK123765	8458	−0.21	−0.18	0.03	−0.40	G2
A_23_P97161	*TTF2*	NM_003594	8458	0.23	−0.05	−0.28	0.25	G2
A_23_P139547	*TUBA1A*	NM_006009	7846	−0.34	−0.17	0.17	0.19	G2
A_23_P128598	*TUBA3C*	NM_006001	7278	0.22	0.66	0.44	−0.22	G2
A_23_P154065	*TUBA4A*	NM_006000	7277	0.66	0.48	−0.18	0.76	G2
A_23_P102109	*TUBA4A*	NM_006000	7277	0.56	0.48	−0.09	0.81	G2
A_23_P154070	*TUBA4A*	NM_006000	7277	−0.45	−0.15	0.30	0.06	G2
A_23_P84448	*TUBA4A*	NM_006000	7277	0.22	0.14	−0.08	0.52	G2
A_23_P387057	*TUBB*	NM_178014	203068	−0.46	−0.41	0.05	0.07	G2
A_23_P81912	*TUBB*	NM_178014	203068	−0.37	−0.37	0.00	0.25	G2
A_32_P78528	*TUBB*	NM_178014	203068	−0.28	−0.16	0.12	0.07	G2
A_23_P19291	*TUBB2A*	NM_001069	7280	1.18	−0.07	−1.25	0.79	G2
A_23_P501276	*TUBB2A*	NM_001069	7280	0.42	−0.32	−0.74	0.72	G2
A_23_P26895	*TUBD1*	NM_016261	51174	−0.02	0.13	0.15	0.14	G2
A_23_P423480	*TYSND1*	NM_173555	219743	−0.21	−0.16	0.05	0.11	G2
A_24_P290585	*UACA*	NM_001008224	55075	0.28	0.01	−0.27	1.38	G2
A_23_P360340	*UACA*	NM_001008224	55075	0.10	−0.23	−0.33	0.55	G2
A_24_P90774	*UACA*	NM_001008224	55075	0.03	0.11	0.07	−0.41	G2
A_24_P297539	*UBE2C*	NM_181803	11065	−2.93	−2.50	0.43	−0.57	G2
A_23_P11936	*UBXN11*	NM_183008	91544	−0.32	−0.37	−0.06	−0.35	G2
A_24_P239811	*UBXN11*	NM_183008	91544	−0.03	0.00	0.03	0.00	G2
A_23_P428298	*UNC5CL*	NM_173561	222643	0.48	0.40	−0.08	−0.09	G2
A_23_P66599	*VPS25*	NM_032353	84313	−0.33	−0.20	0.13	0.02	G2
A_24_P294982	*VTA1*	NM_016485	51534	−0.42	−0.42	0.00	0.18	G2
A_23_P42368	*VTA1*	NM_016485	51534	−0.36	−0.20	0.16	−0.24	G2
A_23_P393766	*WDR62*	NM_173636	284403	0.15	−0.21	−0.36	0.47	G2
A_23_P339705	*WDR62*	NM_173636	284403	−0.02	−0.17	−0.15	−0.03	G2
A_23_P101281	*ZNF587*	NM_032828	84914	−0.17	−0.06	0.11	0.19	G2
A_24_P313804	*ZNF587*	NM_032828	84914	−0.19	−0.14	0.05	0.74	G2
A_24_P373726	*ZNF587*	NM_032828	84914	−0.01	−0.30	−0.28	0.42	G2
A_23_P329286	*ZNHIT2*	NM_014205	741	−0.25	−0.03	0.22	−0.41	G2
A_23_P356684	*ANLN*	NM_018685	54443	−2.41	−2.24	0.17	−0.42	G2,G2_M
A_23_P334845	*ARHGAP19*	NM_032900	84986	0.51	0.47	−0.04	0.34	G2,G2_M
A_23_P1387	*ARHGAP19*	NM_032900	84986	0.00	−0.49	−0.49	0.70	G2,G2_M
A_24_P19337	*ASXL1*	NM_015338	171023	0.61	0.34	−0.26	0.39	G2,G2_M
A_32_P41021	*ASXL1*	NM_015338	171023	−0.32	0.00	0.32	−0.65	G2,G2_M
A_23_P58321	*CCNA2*	NM_001237	890	−2.44	−1.76	0.69	−0.71	G2,G2_M
A_24_P218979	*CDCA3*	NM_031299	83461	−2.19	−2.02	0.17	−0.56	G2,G2_M
A_23_P162476	*CDCA3*	NM_031299	83461	−1.69	−1.43	0.27	−0.36	G2,G2_M
A_23_P209394	*CFLAR*	NM_001127184	8837	1.55	0.95	−0.60	2.06	G2,G2_M
A_24_P120115	*CFLAR*	NM_003879	8837	0.65	0.80	0.15	0.12	G2,G2_M
A_23_P151405	*CKAP2*	NM_018204	26586	−1.71	−0.85	0.87	−0.82	G2,G2_M
A_24_P99090	*CKAP2*	NM_018204	26586	−0.17	−0.05	0.12	0.04	G2,G2_M
A_23_P90062	*DNAJB1*	NM_006145	3337	1.18	0.26	−0.92	0.32	G2,G2_M
A_24_P941759	*G2E3*	NM_017769	55632	−0.44	−0.47	−0.04	−0.04	G2,G2_M
A_23_P99604	*G2E3*	NM_017769	55632	0.22	−0.14	−0.36	0.64	G2,G2_M
A_32_P189204	*GAS2L3*	NM_174942	283431	−0.76	−0.63	0.13	0.22	G2,G2_M
A_32_P37143	*GAS2L3*	BX649059	283431	−0.46	−0.60	−0.14	0.25	G2,G2_M
A_24_P944616	*HP1BP3*	NM_016287	50809	−0.33	−0.08	0.25	−0.95	G2,G2_M
A_23_P137630	*HP1BP3*	NM_016287	50809	−0.28	−0.26	0.02	0.52	G2,G2_M
A_24_P247660	*JPT1*	NM_001002033	51155	−0.50	−0.45	0.05	−0.64	G2,G2_M
A_23_P100632	*JPT1*	NM_001002033	51155	−0.11	0.22	0.33	−0.42	G2,G2_M
A_23_P75071	*KIF20B*	NM_016195	9585	−1.72	−1.70	0.02	−0.31	G2,G2_M
A_23_P86403	*KIF5B*	NM_004521	3799	0.08	−0.01	−0.09	−0.13	G2,G2_M
A_23_P200493	*LBR*	NM_002296	3930	−0.26	−0.23	0.03	0.20	G2,G2_M
A_23_P106162	*MIS18BP1*	NM_018353	55320	−0.89	−0.34	0.55	−0.14	G2,G2_M
A_23_P364107	*MIS18BP1*	NM_018353	55320	−1.10	−0.59	0.52	−0.69	G2,G2_M
A_23_P315843	*NCOA5*	NM_020967	57727	−0.63	0.19	0.82	−1.05	G2,G2_M
A_23_P210515	*NCOA5*	NM_020967	57727	−0.66	0.45	1.12	−1.46	G2,G2_M
A_24_P416079	*NUSAP1*	NM_016359	51203	−2.70	−2.01	0.69	−0.98	G2,G2_M
A_23_P333420	*RANGAP1*	NM_002883	5905	0.07	−0.20	−0.27	0.29	G2,G2_M
A_23_P139066	*RNF141*	NM_016422	50862	0.39	−0.13	−0.53	0.95	G2,G2_M
A_24_P372625	*RNF141*	NM_016422	50862	0.06	−0.02	−0.08	0.14	G2,G2_M
A_23_P100788	*STAT5B*	NM_012448	6777	0.68	0.33	−0.34	0.33	G2,G2_M
A_24_P342178	*STAT5B*	BC020868	6777	0.33	0.08	−0.25	0.46	G2,G2_M
A_32_P187327	*TUBB4B*	NM_006088	10383	−0.26	0.35	0.60	−0.76	G2,G2_M
A_23_P312863	*TUBB4B*	NM_006088	10383	−0.21	0.32	0.53	−0.67	G2,G2_M
A_23_P4353	*WSB1*	NM_015626	26118	1.02	−0.01	−1.03	1.36	G2,G2_M
A_23_P214756	*ADGRG6*	NM_198569	57211	0.15	0.08	−0.07	0.85	G2,S
A_24_P942945	*ADGRG6*	NM_020455	57211	−0.14	−0.28	−0.14	−0.14	G2,S
A_24_P411749	*ADGRG6*	NM_020455	57211	−0.01	−0.21	−0.21	0.28	G2,S
A_23_P118834	*TOP2A*	NM_001067	7153	−3.63	−2.61	1.02	−1.33	G2,S
A_23_P502314	*ADGRE5*	NM_078481	976	−0.13	0.18	0.32	−0.67	G2_M
A_23_P502312	*ADGRE5*	NM_078481	976	0.02	0.39	0.37	−0.76	G2_M
A_23_P30098	*ADH4*	NM_000670	127	−0.24	0.51	0.75	0.10	G2_M
A_23_P27688	*ADM5*	NM_001101340	199800	−0.26	−0.26	0.00	0.09	G2_M
A_23_P436353	*AFDN*	NM_001040001	4301	0.39	0.35	−0.04	−0.39	G2_M
A_23_P256603	*AFDN*	NM_005936	4301	0.11	−0.83	−0.93	0.75	G2_M
A_24_P943484	*AHI1*	NM_017651	54806	0.27	0.29	0.01	0.72	G2_M
A_24_P213710	*AHI1*	NM_017651	54806	−0.24	0.10	0.34	0.06	G2_M
A_24_P38143	*AHI1*	NM_017651	54806	−0.19	0.11	0.30	0.14	G2_M
A_23_P70746	*AHI1*	NM_017651	54806	0.07	0.27	0.19	0.16	G2_M
A_23_P428819	*AKIRIN2*	NM_018064	55122	0.39	0.20	−0.19	−0.07	G2_M
A_23_P428827	*AKIRIN2*	NM_018064	55122	0.11	−0.07	−0.18	−0.37	G2_M
A_23_P20615	*ANP32B*	NM_006401	10541	0.43	0.07	−0.36	0.24	G2_M
A_24_P225468	*ANP32E*	NM_030920	81611	−0.32	−0.68	−0.37	0.30	G2_M
A_23_P160934	*ANP32E*	NM_030920	81611	0.01	−0.39	−0.41	0.90	G2_M
A_23_P151075	*ARHGDIB*	NM_001175	397	−0.28	0.41	0.69	0.07	G2_M
A_24_P356218	*ARL6IP1*	NM_015161	23204	−0.14	−0.27	−0.13	0.08	G2_M
A_23_P118150	*ARL6IP1*	NM_015161	23204	−0.29	−0.32	−0.03	1.16	G2_M
A_23_P91640	*ASPHD2*	NM_020437	57168	−0.55	0.19	0.74	−1.23	G2_M
A_24_P245815	*ASPHD2*	NM_020437	57168	−0.29	0.33	0.62	−1.00	G2_M
A_23_P112950	*ATF7IP*	NM_018179	55729	0.17	0.54	0.37	−0.51	G2_M
A_24_P923757	*ATF7IP*	NM_018179	55729	0.26	0.26	0.00	0.25	G2_M
A_23_P48278	*ATF7IP*	AK001001	55729	−0.01	0.81	0.82	−0.13	G2_M
A_23_P163814	*ATXN1L*	NM_001137675	342371	0.09	−0.31	−0.40	0.20	G2_M
A_23_P131866	*AURKA*	NM_198433	6790	−1.56	−1.01	0.56	0.03	G2_M
A_24_P103803	*B4GALT1*	NM_001497	2683	0.90	0.68	−0.22	−0.45	G2_M
A_23_P135271	*B4GALT1*	NM_001497	2683	0.49	0.22	−0.27	−0.06	G2_M
A_24_P115774	*BIRC2*	NM_001166	329	−0.12	−0.28	−0.16	0.32	G2_M
A_23_P118815	*BIRC5*	NM_001012271	332	−3.41	−2.33	1.08	−1.52	G2_M
A_23_P143331	*BMP2*	NM_001200	650	1.44	0.57	−0.87	0.93	G2_M
A_23_P124417	*BUB1*	NM_004336	699	−3.26	−2.47	0.79	−0.75	G2_M
A_23_P163481	*BUB1B*	NM_001211	701	−3.08	−2.32	0.76	−1.02	G2_M
A_23_P92928	*C6*	NM_000065	729	−0.19	−0.33	−0.14	−0.46	G2_M
A_23_P28664	*CCDC88A*	NM_018084	55704	0.71	0.22	−0.49	0.70	G2_M
A_24_P20120	*CCDC88A*	NM_018084	55704	0.52	0.40	−0.12	0.49	G2_M
A_23_P17269	*CCDC88A*	NM_018084	55704	0.36	−0.01	−0.37	0.82	G2_M
A_24_P28739	*CCDC88A*	NM_018084	55704	0.32	−0.17	−0.49	0.41	G2_M
A_23_P122197	*CCNB1*	NM_031966	891	−2.14	−1.49	0.65	−0.27	G2_M
A_23_P65757	*CCNB2*	NM_004701	9133	−3.05	−2.42	0.64	−0.70	G2_M
A_32_P72822	*CCNB2*	AK023404	9133	0.52	0.74	0.22	0.59	G2_M
A_23_P202837	*CCND1*	NM_053056	595	−0.15	−0.20	−0.05	−0.10	G2_M
A_24_P124550	*CCND1*	NM_053056	595	−0.03	−0.15	−0.12	−0.75	G2_M
A_24_P193011	*CCND1*	NM_053056	595	−0.02	0.09	0.11	−1.15	G2_M
A_23_P366908	*CCSAP*	NM_145257	126731	0.38	−0.19	−0.57	0.18	G2_M
A_24_P930764	*CCSAP*	BC039241	126731	0.40	−0.35	−0.75	0.71	G2_M
A_32_P83776	*CCSAP*	NM_145257	126731	0.42	0.13	−0.29	−1.01	G2_M
A_23_P149200	*CDC20*	NM_001255	991	−1.36	−1.65	−0.28	0.10	G2_M
A_23_P210726	*CDC25B*	NM_021873	994	−0.75	−0.03	0.72	−0.74	G2_M
A_23_P66777	*CDC27*	NM_001256	996	0.05	0.25	0.20	0.28	G2_M
A_23_P166453	*CDC42EP1*	NM_152243	11135	−0.06	0.25	0.31	−0.98	G2_M
A_24_P143032	*CDC42EP1*	NM_152243	11135	0.06	0.25	0.19	−0.63	G2_M
A_23_P89941	*CDKN2D*	NM_001800	1032	0.50	−0.51	−1.01	0.79	G2_M
A_24_P413884	*CENPA*	NM_001809	1058	−2.99	−2.34	0.65	−1.30	G2_M
A_23_P253524	*CENPE*	NM_001813	1062	−2.90	−1.84	1.06	−1.03	G2_M
A_23_P401	*CENPF*	NM_016343	1063	−3.83	−2.46	1.37	−1.17	G2_M
A_24_P96780	*CENPF*	NM_016343	1063	−1.58	−0.86	0.72	−0.66	G2_M
A_23_P115872	*CEP55*	NM_018131	55165	−2.96	−2.26	0.71	−0.74	G2_M
A_23_P420551	*CIT*	NM_007174	11113	−2.31	−1.43	0.88	−1.92	G2_M
A_23_P135977	*CKAP5*	NM_001008938	9793	−0.65	−0.56	0.09	−0.09	G2_M
A_24_P300841	*CKAP5*	NM_001008938	9793	−0.27	−0.13	0.14	−0.11	G2_M
A_23_P45917	*CKS1B*	NM_001826	1163	−0.90	−1.00	−0.10	0.48	G2_M
A_32_P206698	*CKS1B*	NM_001826	1163	−0.79	−0.88	−0.08	0.51	G2_M
A_32_P192430	*CKS1B*	NM_001826	1163	−0.52	−0.67	−0.15	0.44	G2_M
A_23_P71727	*CKS2*	NM_001827	1164	−0.85	−1.06	−0.21	0.78	G2_M
A_23_P16673	*CNN2*	NM_004368	1265	0.34	0.36	0.01	−0.98	G2_M
A_24_P142743	*CNN2*	NM_004368	1265	0.15	0.29	0.14	0.07	G2_M
A_23_P9768	*CNTROB*	NM_053051	116840	−0.07	−0.01	0.06	0.00	G2_M
A_23_P9761	*CNTROB*	NM_001037144	116840	−0.02	0.10	0.12	−0.45	G2_M
A_23_P404606	*CREBRF*	NM_153607	153222	0.59	−0.30	−0.88	1.08	G2_M
A_23_P134835	*CSGALNACT1*	NM_018371	55790	1.04	0.75	−0.29	1.04	G2_M
A_24_P406525	*CSGALNACT1*	NM_018371	55790	0.10	0.31	0.21	0.25	G2_M
A_23_P58647	*CTNNA1*	NM_001903	1495	0.06	0.36	0.30	−0.44	G2_M
A_24_P80633	*CTNNA1*	NM_001903	1495	0.03	0.05	0.02	−0.27	G2_M
A_24_P881527	*CTNND1*	NM_001085458	1500	−0.14	0.09	0.23	−0.46	G2_M
A_23_P95080	*CTNND1*	NM_001085461	1500	0.13	−0.15	−0.28	0.76	G2_M
A_23_P251316	*CTNND1*	NM_001331	1500	−0.12	−0.21	−0.09	−0.15	G2_M
A_24_P38930	*CTNND1*	NM_001331	1500	0.07	−0.03	−0.10	−0.22	G2_M
A_23_P200310	*DEPDC1*	NM_017779	55635	−2.48	−2.08	0.40	−0.16	G2_M
A_24_P25872	*DEPDC1*	NM_017779	55635	−1.13	−1.41	−0.28	−0.32	G2_M
A_23_P361419	*DEPDC1B*	NM_018369	55789	−1.89	−1.25	0.63	−0.44	G2_M
A_23_P88331	*DLGAP5*	NM_014750	9787	−3.42	−2.36	1.06	−1.16	G2_M
A_24_P9671	*DNAJA1*	NM_001539	3301	0.95	0.69	−0.26	1.02	G2_M
A_24_P192586	*DNAJA1*	ENST00000330899	3301	−0.49	−0.02	0.47	0.14	G2_M
A_23_P60479	*DNAJA1*	NM_001539	3301	0.31	0.26	−0.05	0.90	G2_M
A_23_P63205	*DR1*	NM_001938	1810	0.30	−0.06	−0.36	0.37	G2_M
A_23_P391725	*DR1*	NM_001938	1810	0.13	0.16	0.03	0.01	G2_M
A_23_P134935	*DUSP4*	NM_001394	1846	−0.31	−0.20	0.12	−0.96	G2_M
A_23_P144165	*DZIP3*	NM_014648	9666	0.64	0.09	−0.55	0.75	G2_M
A_24_P102504	*DZIP3*	NM_014648	9666	−0.14	−0.30	−0.16	0.24	G2_M
A_23_P435051	*DZIP3*	ENST00000463306	9666	0.06	−0.22	−0.28	0.25	G2_M
A_23_P31721	*E2F5*	NM_001951	1875	−0.48	−0.02	0.46	−0.36	G2_M
A_23_P9574	*ECT2*	NM_018098	1894	−0.96	−0.37	0.59	0.16	G2_M
A_23_P44684	*ECT2*	NM_018098	1894	−1.09	−0.50	0.59	−0.44	G2_M
A_24_P366033	*ECT2*	NM_018098	1894	−0.29	−0.31	−0.02	0.38	G2_M
A_24_P282251	*FGA*	NM_021871	2243	0.26	−0.16	−0.42	−0.04	G2_M
A_23_P44274	*FGA*	NM_000508	2243	−0.08	0.01	0.09	0.24	G2_M
A_23_P375372	*FGA*	NM_021871	2243	0.07	0.62	0.55	−0.54	G2_M
A_23_P151150	*FOXM1*	NM_202002	2305	−1.27	−1.19	0.07	−1.05	G2_M
A_23_P363778	*FRZB*	NM_001463	2487	1.24	0.54	−0.70	1.02	G2_M
A_23_P10902	*FRZB*	NM_001463	2487	0.95	0.41	−0.54	0.75	G2_M
A_23_P502142	*FYN*	NM_002037	2534	−0.54	−0.35	0.20	−1.32	G2_M
A_23_P23221	*GADD45A*	NM_001924	1647	0.64	0.07	−0.58	−0.01	G2_M
A_23_P146922	*GAS6*	NM_000820	2621	0.18	0.16	−0.02	−0.78	G2_M
A_23_P105251	*GLI1*	NM_005269	2735	0.06	0.17	0.12	−0.31	G2_M
A_23_P63825	*GOT1*	NM_002079	2805	0.29	0.53	0.24	0.44	G2_M
A_24_P81473	*GOT1*	NM_002079	2805	0.20	0.18	−0.01	0.64	G2_M
A_23_P63402	*GPSM2*	NM_013296	29899	−1.24	−0.96	0.28	−0.28	G2_M
A_24_P273132	*GPSM2*	NM_013296	29899	−0.47	−0.69	−0.23	−0.02	G2_M
A_23_P257256	*GRK6*	NM_002082	2870	−0.18	0.03	0.21	−0.53	G2_M
A_23_P152420	*GSE1*	NM_014615	23199	0.19	0.03	−0.16	−0.38	G2_M
A_24_P943062	*GSE1*	NM_014615	23199	−0.11	0.18	0.29	−1.06	G2_M
A_23_P57588	*GTSE1*	NM_016426	51512	−2.01	−1.57	0.43	−0.89	G2_M
A_23_P125771	*HCFC1*	NM_005334	3054	−0.26	0.18	0.45	−1.13	G2_M
A_23_P168490	*HERPUD2*	NM_022373	64224	0.03	0.00	−0.03	−0.02	G2_M
A_23_P119543	*HMG20B*	NM_006339	10362	−0.82	−0.34	0.48	−0.54	G2_M
A_23_P217236	*HMGB3*	NM_005342	3149	−0.75	−0.79	−0.04	−0.06	G2_M
A_23_P70007	*HMMR*	NM_012484	3161	−3.37	−2.64	0.73	−0.46	G2_M
A_23_P109442	*HPS4*	NM_022081	89781	0.72	0.36	−0.36	−0.05	G2_M
A_23_P109446	*HPS4*	NM_022081	89781	0.58	0.44	−0.14	−0.34	G2_M
A_23_P17606	*HSPA13*	NM_006948	6782	1.40	0.56	−0.85	2.40	G2_M
A_24_P134392	*HSPA13*	NM_006948	6782	0.30	−0.05	−0.35	0.52	G2_M
A_23_P70547	*HSPA1L*	NM_005527	3305	1.11	0.14	−0.96	0.90	G2_M
A_32_P13728	*HSPA8*	NM_006597	3312	0.36	0.40	0.03	0.34	G2_M
A_24_P295745	*HSPA8*	NM_153201	3312	0.13	0.12	−0.01	0.78	G2_M
A_24_P287129	*HSPA8*	NM_006597	3312	0.05	0.06	0.02	0.10	G2_M
A_23_P24594	*HSPA8*	NM_006597	3312	0.03	0.12	0.09	0.17	G2_M
A_23_P410600	*IDI2*	NM_033261	91734	0.14	−0.01	−0.16	−0.21	G2_M
A_23_P112026	*IDO1*	NM_002164	3620	0.69	0.85	0.16	0.41	G2_M
A_23_P55076	*INPP5K*	NM_130766	51763	0.47	0.28	−0.19	−0.09	G2_M
A_24_P279328	*INPP5K*	NM_130766	51763	−0.09	−0.12	−0.02	−0.42	G2_M
A_23_P109184	*INSM1*	NM_002196	3642	1.19	0.87	−0.32	−0.25	G2_M
A_24_P31676	*INSM1*	NM_002196	3642	−0.05	0.13	0.17	−0.08	G2_M
A_23_P92042	*ITPR1*	NM_002222	3708	0.17	0.24	0.07	−0.72	G2_M
A_23_P156198	*JADE2*	NM_015288	23338	1.15	0.18	−0.97	0.77	G2_M
A_24_P226278	*JADE2*	NM_015288	23338	0.53	−0.07	−0.60	0.28	G2_M
A_23_P416434	*JADE2*	NM_015288	23338	−0.17	0.47	0.64	−0.91	G2_M
A_24_P927883	*JADE2*	A_24_P927883	NA	−0.02	0.29	0.31	−0.10	G2_M
A_24_P324011	*KCTD2*	NM_015353	23510	−0.20	−0.03	0.17	−0.55	G2_M
A_32_P160693	*KCTD2*	NM_015353	23510	−0.02	−0.13	−0.11	−0.13	G2_M
A_23_P149668	*KIF14*	NM_014875	9928	−0.68	−0.91	−0.23	−0.16	G2_M
A_23_P34788	*KIF2C*	NM_006845	11004	−3.33	−2.53	0.80	−0.82	G2_M
A_23_P415401	*KLF9*	NM_001206	687	0.04	−0.15	−0.20	−0.41	G2_M
A_23_P86100	*KLHDC9*	NM_001007255	126823	−0.01	−0.26	−0.25	0.04	G2_M
A_32_P82807	*KMT5A*	NM_020382	387893	0.38	0.39	0.02	0.29	G2_M
A_24_P238855	*KMT5A*	NM_020382	387893	−0.22	0.33	0.54	−0.29	G2_M
A_32_P191527	*KMT5A*	NM_020382	387893	−0.19	0.07	0.26	−0.57	G2_M
A_32_P191859	*KMT5A*	NM_020382	387893	−0.10	0.11	0.21	−0.39	G2_M
A_23_P217968	*KMT5B*	NM_016028	51111	1.11	0.43	−0.68	0.99	G2_M
A_23_P326739	*KMT5B*	NM_017635	51111	−0.60	−0.03	0.57	−0.90	G2_M
A_23_P96688	*KMT5B*	NM_016028	51111	−0.02	−0.15	−0.13	0.10	G2_M
A_23_P140705	*KNSTRN*	NM_001142761	90417	−1.55	−1.01	0.54	0.14	G2_M
A_24_P162718	*LMNA*	NM_005572	4000	−0.08	−0.12	−0.04	−0.35	G2_M
A_23_P34835	*LMNA*	NM_005572	4000	−0.01	−0.01	0.01	−0.44	G2_M
A_23_P251118	*LPP*	NM_005578	4026	0.44	0.47	0.03	0.31	G2_M
A_24_P916515	*LPP*	A_24_P916515	NA	0.33	0.35	0.01	−0.02	G2_M
A_32_P70519	*LPP*	ENST00000312675	4026	0.16	0.16	0.00	−0.46	G2_M
A_24_P114551	*LPP*	NM_005578	4026	−0.14	0.07	0.21	−0.89	G2_M
A_32_P39049	*LPP*	ENST00000312675	4026	−0.13	0.10	0.24	−0.09	G2_M
A_32_P193218	*LPP*	ENST00000312675	4026	−0.10	0.18	0.28	−0.83	G2_M
A_24_P778741	*LPP*	ENST00000312675	4026	0.10	0.37	0.27	−1.09	G2_M
A_32_P56874	*LPP*	ENST00000312675	4026	0.02	0.06	0.03	−0.35	G2_M
A_23_P200222	*LRP8*	NM_033300	7804	0.86	0.93	0.08	0.31	G2_M
A_23_P34325	*LRP8*	NM_033300	7804	0.14	0.53	0.38	−0.26	G2_M
A_23_P253958	*LRRC17*	NM_005824	10234	0.43	1.01	0.58	−0.15	G2_M
A_23_P145376	*MAPK13*	NM_002754	5603	0.22	−0.13	−0.35	0.82	G2_M
A_24_P406132	*MAPK13*	NM_002754	5603	0.08	−0.03	−0.12	0.20	G2_M
A_23_P71328	*MATN2*	NM_030583	4147	−0.50	−0.30	0.20	−1.34	G2_M
A_24_P179225	*MATN2*	NM_030583	4147	0.24	0.06	−0.17	−0.24	G2_M
A_23_P19455	*MDC1*	NM_014641	9656	−0.14	−0.15	−0.01	−0.40	G2_M
A_23_P105227	*ME3*	NM_001014811	10873	−0.40	0.15	0.55	−0.26	G2_M
A_23_P116614	*ME3*	NM_001014811	10873	0.04	0.39	0.35	0.08	G2_M
A_24_P346855	*MKI67*	NM_002417	4288	−1.66	−1.70	−0.04	−0.93	G2_M
A_32_P9382	*MZT1*	NM_001071775	440145	−0.33	−0.50	−0.18	0.57	G2_M
A_24_P532589	*MZT1*	NM_001071775	440145	−0.12	−0.28	−0.16	0.18	G2_M
A_24_P210675	*NDE1*	NM_017668	54820	−0.60	0.01	0.61	−1.05	G2_M
A_23_P206901	*NDE1*	NM_017668	54820	0.07	0.63	0.56	−0.46	G2_M
A_23_P35219	*NEK2*	NM_002497	4751	−1.67	−1.54	0.13	−0.25	G2_M
A_24_P319613	*NEK2*	NM_002497	4751	−0.86	−0.50	0.36	−0.09	G2_M
A_23_P74349	*NUF2*	NM_145697	83540	−3.08	−2.39	0.69	−1.08	G2_M
A_23_P102320	*NUP35*	NM_138285	129401	−0.43	−0.18	0.25	0.55	G2_M
A_23_P203586	*NUP98*	NM_016320	4928	0.32	0.34	0.02	−0.05	G2_M
A_24_P141522	*NUP98*	NM_016320	4928	0.29	0.24	−0.06	0.12	G2_M
A_23_P308032	*NUP98*	NM_005387	4928	−0.12	−0.28	−0.16	0.14	G2_M
A_23_P60488	*ODF2*	NM_002540	4957	−0.72	−0.22	0.50	−0.90	G2_M
A_23_P71889	*ODF2*	NM_153437	4957	−0.21	0.30	0.51	−0.15	G2_M
A_23_P63777	*OIT3*	NM_152635	170392	−0.28	0.06	0.34	0.06	G2_M
A_24_P124624	*OLR1*	NM_002543	4973	0.54	−0.05	−0.59	0.51	G2_M
A_24_P274072	*PATJ*	NM_176877	10207	0.45	0.06	−0.39	0.74	G2_M
A_23_P126100	*PATJ*	NM_176877	10207	0.39	0.15	−0.24	0.60	G2_M
A_32_P88958	*PATJ*	NM_176877	10207	0.20	−0.13	−0.33	0.41	G2_M
A_23_P321034	*PATJ*	NM_176877	10207	0.17	0.33	0.17	−0.25	G2_M
A_24_P942454	*PATJ*	NM_176877	10207	−0.05	0.06	0.11	0.50	G2_M
A_32_P62997	*PBK*	NM_018492	55872	−4.16	−3.00	1.15	−1.67	G2_M
A_23_P116578	*PCF11*	NM_015885	51585	−0.41	−0.40	0.01	0.36	G2_M
A_24_P835763	*PCF11*	A_24_P835763	NA	0.38	−0.66	−1.05	1.16	G2_M
A_23_P33303	*PIK3CD*	NM_005026	5293	0.56	0.53	−0.03	−0.27	G2_M
A_24_P71244	*PIK3CD*	NM_005026	5293	0.15	−0.05	−0.20	−0.21	G2_M
A_23_P259833	*PIK3CD*	NM_005026	5293	0.02	−0.13	−0.15	0.13	G2_M
A_23_P49878	*PIMREG*	NM_019013	54478	−2.67	−2.40	0.27	−0.54	G2_M
A_23_P411723	*PLAG1*	NM_002655	5324	0.44	−0.18	−0.62	1.02	G2_M
A_24_P313504	*PLK1*	NM_005030	5347	−1.32	−1.37	−0.05	−0.17	G2_M
A_23_P118174	*PLK1*	NM_005030	5347	−0.82	−0.68	0.14	−0.43	G2_M
A_24_P354300	*POC1A*	NM_015426	25886	−1.21	−0.81	0.40	−0.63	G2_M
A_23_P212284	*POC1A*	NM_015426	25886	−1.26	−0.82	0.44	−0.77	G2_M
A_24_P349002	*POM121*	NM_172020	9883	0.32	0.50	0.19	−0.52	G2_M
A_32_P131940	*POM121*	NM_172020	9883	0.27	0.40	0.13	−0.95	G2_M
A_24_P417784	*POM121*	NM_172020	9883	0.35	0.57	0.22	−0.86	G2_M
A_24_P266273	*POM121*	NM_172020	9883	0.03	−0.19	−0.22	0.06	G2_M
A_23_P156667	*PPP1R10*	NM_002714	5514	−0.93	0.07	1.00	−1.19	G2_M
A_23_P15305	*PRPSAP1*	NM_002766	5635	−0.51	−0.31	0.20	−0.68	G2_M
A_23_P80382	*PRR5*	NM_015366	55615	−0.21	0.00	0.21	−0.48	G2_M
A_24_P10890	*PRR5*	NM_015366	55615	0.20	0.06	−0.14	−0.15	G2_M
A_24_P21044	*PSMG3*	NM_032302	84262	−0.03	0.12	0.15	−0.11	G2_M
A_23_P103328	*PTGER3*	NM_198714	5733	1.30	1.41	0.11	0.82	G2_M
A_24_P945365	*PTGER3*	NM_198715	5733	0.53	0.61	0.08	−0.09	G2_M
A_23_P81770	*PTP4A1*	NM_003463	7803	0.67	0.28	−0.39	0.32	G2_M
A_24_P294832	*PTP4A1*	NM_003463	7803	−0.37	−0.01	0.36	−0.57	G2_M
A_24_P252043	*PTP4A1*	NM_003463	7803	0.50	−0.24	−0.74	0.60	G2_M
A_23_P124486	*PTPN9*	NM_002833	5780	−0.51	−0.22	0.29	−1.07	G2_M
A_23_P13632	*PYM1*	NM_032345	84305	−0.42	−0.12	0.30	−0.64	G2_M
A_23_P45106	*QRICH1*	NM_017730	54870	0.22	0.14	−0.08	−0.53	G2_M
A_23_P45108	*QRICH1*	NM_017730	54870	0.17	−0.10	−0.27	0.53	G2_M
A_23_P207014	*RAD51C*	NM_002876	5889	−0.96	−0.75	0.21	0.02	G2_M
A_23_P391344	*RASGEF1A*	BC022548	221002	0.73	0.39	−0.34	0.62	G2_M
A_32_P223140	*RASGEF1A*	NM_145313	221002	0.75	0.14	−0.62	1.04	G2_M
A_24_P79955	*RBM8A*	NM_005105	9939	−0.54	−0.12	0.41	−0.54	G2_M
A_23_P305335	*RBM8A*	BC017770	9939	1.29	0.20	−1.09	1.74	G2_M
A_32_P62571	*RBM8A*	NM_005105	9939	−0.41	0.02	0.44	−0.81	G2_M
A_23_P104116	*RBM8A*	NM_005105	9939	0.09	0.16	0.07	0.49	G2_M
A_23_P166248	*RCAN1*	NM_004414	1827	−0.18	−0.13	0.05	−0.24	G2_M
A_23_P14105	*RCBTB2*	NM_001268	1102	−1.53	−0.06	1.47	−1.74	G2_M
A_24_P342591	*RERE*	NM_012102	473	0.16	0.39	0.23	−1.12	G2_M
A_23_P85414	*RERE*	NM_012102	473	0.04	0.07	0.04	−0.65	G2_M
A_24_P725630	*RNPS1*	NM_006711	10921	0.85	0.54	−0.31	0.39	G2_M
A_24_P766577	*RNPS1*	NM_006711	10921	0.12	0.02	−0.10	−0.21	G2_M
A_23_P152272	*RNPS1*	NM_006711	10921	0.02	0.08	0.06	−0.38	G2_M
A_23_P80129	*RRP1*	NM_003683	8568	0.63	0.52	−0.11	0.04	G2_M
A_23_P80136	*RRP1*	NM_003683	8568	−0.09	0.26	0.34	−0.03	G2_M
A_24_P100517	*SAPCD2*	NM_178448	89958	−0.91	−0.60	0.31	−0.42	G2_M
A_23_P370625	*SELENON*	NM_020451	57190	−0.80	−0.26	0.54	−1.75	G2_M
A_24_P231250	*SELENON*	NM_020451	57190	−0.37	−0.07	0.30	−1.06	G2_M
A_24_P105283	*SFPQ*	NM_005066	6421	−0.18	−0.09	0.09	−0.28	G2_M
A_23_P411335	*SGO2*	NM_152524	151246	−1.95	−1.54	0.41	−0.59	G2_M
A_32_P96719	*SHCBP1*	NM_024745	79801	−1.68	−1.83	−0.14	−0.60	G2_M
A_23_P59051	*SLC17A2*	NM_005835	10246	0.13	0.43	0.29	−0.36	G2_M
A_24_P10657	*SLC44A2*	NM_020428	57153	0.24	0.31	0.07	−0.65	G2_M
A_23_P208340	*SLC44A2*	NM_020428	57153	−0.08	0.15	0.23	−0.96	G2_M
A_23_P68824	*SMARCB1*	NM_003073	6598	−0.33	0.02	0.35	−0.82	G2_M
A_24_P232696	*SMARCD1*	NM_139071	6602	−0.23	−0.01	0.22	−0.68	G2_M
A_23_P204745	*SMARCD1*	NM_139071	6602	0.16	0.65	0.49	−0.24	G2_M
A_23_P211428	*SMTN*	NM_134269	6525	−0.47	−0.31	0.17	−1.05	G2_M
A_23_P5934	*SOGA1*	AB020696	140710	−0.43	−0.12	0.31	−0.40	G2_M
A_23_P5936	*SOGA1*	AB020696	140710	−0.26	−0.44	−0.18	−0.42	G2_M
A_23_P5938	*SOGA1*	AB020696	140710	0.18	0.27	0.08	−0.36	G2_M
A_23_P89509	*SPAG5*	NM_006461	10615	−2.17	−1.39	0.78	−0.41	G2_M
A_23_P41948	*SPDL1*	NM_017785	54908	−1.58	−0.78	0.80	−0.55	G2_M
A_23_P102523	*SPTBN1*	NM_003128	6711	−0.45	−0.37	0.08	−0.81	G2_M
A_23_P339095	*SPTBN1*	NM_178313	6711	−0.19	−0.25	−0.07	−0.15	G2_M
A_23_P30223	SRD5A1	NM_001047	6715	−0.15	−0.47	−0.32	−0.08	G2_M
A_23_P413761	*SRSF3*	NM_003017	6428	−0.31	0.03	0.33	−0.18	G2_M
A_23_P19702	*TAB2*	NM_015093	23118	0.32	0.20	−0.12	0.03	G2_M
A_24_P245778	*TFF3*	ENST00000291525	7033	−0.63	−0.48	0.15	−0.45	G2_M
A_23_P393099	*TFF3*	NM_003226	7033	−0.11	−0.09	0.02	−0.05	G2_M
A_24_P289208	*TFF3*	NM_003226	7033	0.14	−0.41	−0.54	0.60	G2_M
A_23_P257296	*TFF3*	NM_003226	7033	0.03	0.03	0.00	0.06	G2_M
A_23_P153197	*TGIF1*	NM_170695	7050	−0.28	−0.22	0.06	−1.29	G2_M
A_24_P926367	*THRAP3*	NM_005119	9967	−0.61	−0.46	0.15	−0.71	G2_M
A_24_P256863	*THRAP3*	NM_005119	9967	−0.19	−0.38	−0.18	0.33	G2_M
A_23_P160367	*THRAP3*	NM_005119	9967	−0.18	−0.42	−0.24	−0.01	G2_M
A_23_P158277	*TMCO4*	NM_181719	255104	−0.15	0.32	0.46	−0.61	G2_M
A_23_P24723	*TMEM138*	NM_016464	51524	−0.43	−0.25	0.17	0.17	G2_M
A_23_P428875	*TNFAIP8L1*	NM_152362	126282	−0.70	−0.84	−0.14	−0.55	G2_M
A_23_P156101	*TNPO1*	NM_002270	3842	−0.17	−0.20	−0.04	0.49	G2_M
A_32_P99097	*TNPO1*	NM_002270	3842	−0.03	0.19	0.22	0.29	G2_M
A_24_P260440	*TNPO1*	NM_002270	3842	0.00	0.10	0.10	0.32	G2_M
A_24_P199097	*TOMM34*	NM_006809	10953	0.31	0.09	−0.22	1.37	G2_M
A_23_P57033	*TOMM34*	NM_006809	10953	0.08	−0.19	−0.27	0.78	G2_M
A_23_P68610	*TPX2*	NM_012112	22974	−2.37	−1.66	0.71	−1.18	G2_M
A_24_P277576	*TRIP13*	NM_004237	9319	−0.28	−0.08	0.21	−0.37	G2_M
A_23_P75839	*TSG101*	NM_006292	7251	0.10	0.06	−0.04	1.78	G2_M
A_23_P162142	*TSKU*	NM_015516	25987	0.51	0.27	−0.24	−0.24	G2_M
A_23_P28105	*TSN*	NM_004622	7247	−0.28	−0.10	0.18	−0.10	G2_M
A_24_P242820	*TSN*	NM_004622	7247	−0.19	−0.19	0.00	−0.29	G2_M
A_23_P259586	*TTK*	NM_003318	7272	−2.35	−2.45	−0.10	−0.31	G2_M
A_24_P263524	*TXNDC9*	NM_005783	10190	−0.65	−0.41	0.24	0.04	G2_M
A_23_P154330	*TXNDC9*	NM_005783	10190	−0.51	−0.20	0.32	0.23	G2_M
A_24_P362646	*TXNDC9*	NM_005783	10190	−0.32	−0.08	0.24	0.18	G2_M
A_23_P204581	*TXNRD1*	NM_003330	7296	0.59	0.55	−0.04	0.70	G2_M
A_23_P40989	*USP13*	NM_003940	8975	−0.40	−0.38	0.03	−0.37	G2_M
A_23_P257911	*USP16*	NM_001032410	10600	−0.34	−0.07	0.27	0.58	G2_M
A_24_P199655	*VANGL1*	NM_138959	81839	−0.63	0.04	0.66	−0.93	G2_M
A_23_P103795	*VANGL1*	NM_138959	81839	−0.30	0.26	0.57	−0.97	G2_M
A_23_P369316	*VANGL1*	NM_138959	81839	−0.17	0.00	0.18	−0.46	G2_M
A_23_P103070	*YWHAH*	NM_003405	7533	−0.19	−0.35	−0.16	−0.35	G2_M
A_23_P215088	*ZC3HC1*	NM_016478	51530	0.02	0.09	0.07	0.32	G2_M
A_24_P290527	*ZFX*	NM_003410	7543	−0.28	−0.08	0.21	0.18	G2_M
A_23_P125639	*ZFX*	NM_003410	7543	0.36	0.08	−0.28	0.75	G2_M
A_24_P940524	*ZFX*	NM_003410	7543	−0.16	0.13	0.29	−0.31	G2_M
A_23_P161091	*ZMYM1*	NM_024772	79830	−0.12	−0.30	−0.18	0.27	G2_M
A_24_P53985	*ZMYM1*	NM_024772	79830	−0.10	−0.25	−0.15	0.38	G2_M
A_23_P159027	*ZNF521*	NM_015461	25925	−0.33	0.08	0.41	−0.63	G2_M
A_23_P78018	*ABCA5*	NM_018672	23461	−0.15	−0.02	0.13	−0.05	S
A_24_P67096	*ABCA5*	NM_018672	23461	−0.15	−0.01	0.14	−0.10	S
A_23_P158976	*ABCC2*	NM_000392	1244	0.45	0.07	−0.38	0.36	S
A_23_P44569	*ABCC2*	NM_000392	1244	0.12	0.14	0.02	0.26	S
A_23_P212665	*ABCC5*	NM_005688	10057	0.29	−0.21	−0.50	−0.14	S
A_23_P258221	*ABCC5*	NM_005688	10057	−0.08	0.07	0.15	−0.03	S
A_24_P268662	*ABHD10*	NM_018394	55347	−0.37	0.38	0.75	−0.08	S
A_23_P92213	*ABHD10*	NM_018394	55347	−0.18	−0.37	−0.19	0.56	S
A_24_P308590	*ABHD10*	NM_018394	55347	0.11	−0.07	−0.19	0.04	S
A_23_P23630	*ACAP3*	NM_030649	116983	−0.69	−0.34	0.35	−0.14	S
A_23_P23625	*ACAP3*	NM_030649	116983	−0.61	−0.11	0.50	−0.52	S
A_23_P12231	*ACAP3*	NM_030649	116983	0.26	−0.45	−0.71	−0.20	S
A_24_P281497	*ACAP3*	NM_030649	116983	−0.15	0.41	0.55	−0.17	S
A_24_P355006	*ADAM22*	ENST00000398204	53616	−0.54	−0.45	0.09	−0.53	S
A_23_P215625	*ADAM22*	NM_021723	53616	−0.37	−0.61	−0.24	−0.10	S
A_24_P243741	*ADAM22*	NM_021721	53616	0.22	0.27	0.06	−0.17	S
A_24_P203630	*ANKRD36*	NM_001164315	375248	0.93	0.78	−0.15	0.57	S
A_24_P6725	*ANKRD36*	NM_001164315	375248	0.78	0.66	−0.12	0.32	S
A_24_P686992	*ANKRD36*	NM_001164315	375248	0.67	0.50	−0.17	0.58	S
A_24_P336931	*ANKRD36*	NM_001164315	375248	−0.02	−0.39	−0.36	0.53	S
A_23_P119254	*ASF1B*	NM_018154	55723	−2.77	−2.37	0.40	−1.54	S
A_23_P120629	*ASIP*	NM_001672	434	0.04	−0.21	−0.25	0.38	S
A_23_P106835	*BBS2*	NM_031885	583	−0.11	−0.25	−0.14	0.10	S
A_23_P105833	*BIVM*	NM_017693	54841	−0.28	0.14	0.43	−0.75	S
A_23_P88630	*BLM*	NM_000057	641	−2.28	−1.89	0.39	−0.83	S
A_24_P303989	*BMI1*	NM_005180	648	−0.26	−0.03	0.23	0.05	S
A_23_P314115	*BMI1*	NM_005180	648	−0.12	−0.01	0.10	0.02	S
A_23_P207400	*BRCA1*	NM_007300	672	−1.00	−0.91	0.08	−0.65	S
A_23_P15844	*BRIP1*	NM_032043	83990	−1.30	−1.16	0.14	0.07	S
A_24_P255524	*CALD1*	AF247820	800	0.54	0.69	0.15	0.28	S
A_24_P921366	*CALD1*	NM_033138	800	−0.51	−0.17	0.34	−0.79	S
A_23_P42575	*CALD1*	NM_033138	800	−0.33	0.02	0.35	−0.35	S
A_24_P313993	*CAPS*	NM_004058	828	−0.42	0.14	0.56	−0.19	S
A_23_P78958	*CAPS*	NM_004058	828	−0.22	0.44	0.66	−0.43	S
A_23_P384056	*CCDC14*	NM_022757	64770	−0.59	−0.46	0.13	0.41	S
A_23_P39574	*CCDC150*	NM_001080539	284992	−1.38	−1.34	0.04	0.15	S
A_24_P157156	*CCDC150*	NM_001080539	284992	−0.47	−0.52	−0.05	−0.24	S
A_23_P320190	*CCDC150*	A_23_P320190	NA	−0.34	−0.24	0.10	−0.53	S
A_24_P636332	*CCDC84*	NM_198489	338657	0.17	0.21	0.04	0.76	S
A_24_P693946	*CCDC84*	A_24_P693946	NA	0.05	−0.01	−0.06	0.62	S
A_23_P57379	*CDC45*	NM_003504	8318	−3.57	−2.37	1.20	−1.90	S
A_23_P148807	*CDC7*	NM_003503	8317	−0.70	−0.50	0.20	−0.07	S
A_23_P104651	*CDCA5*	NM_080668	113130	−2.58	−2.15	0.42	−1.16	S
A_23_P405267	*CDH24*	AK057922	64403	1.03	−0.05	−1.08	1.04	S
A_23_P25790	*CDH24*	NM_022478	64403	0.26	−0.37	−0.62	0.19	S
A_23_P258002	*CDKN2AIP*	NM_017632	55602	0.18	−0.17	−0.35	0.51	S
A_24_P399888	*CENPM*	NM_001002876	79019	−2.65	−2.27	0.38	−0.97	S
A_23_P70328	*CENPQ*	NM_018132	55166	−0.18	−0.36	−0.18	0.41	S
A_23_P254733	*CENPU*	NM_024629	79682	−1.79	−1.85	−0.06	−0.23	S
A_24_P289366	*CERS6*	NM_203463	253782	0.10	0.18	0.08	−0.46	S
A_32_P5480	*CERS6*	NM_203463	253782	−0.08	0.35	0.43	−0.45	S
A_23_P144071	*COL7A1*	NM_000094	1294	−0.17	0.28	0.46	−0.03	S
A_24_P932308	*COQ9*	AK075438	57017	0.90	0.78	−0.12	1.05	S
A_23_P14928	*COQ9*	NM_020312	57017	−0.08	0.22	0.31	−0.15	S
A_23_P87556	*CPNE8*	NM_153634	144402	−0.23	0.33	0.56	−0.20	S
A_24_P56240	*CPNE8*	NM_153634	144402	0.05	0.58	0.53	0.06	S
A_23_P144438	*DCAF16*	NM_017741	54876	0.31	0.11	−0.20	0.31	S
A_32_P104000	*DCUN1D3*	NM_173475	123879	−0.18	0.11	0.30	0.02	S
A_23_P429491	*DDIAS*	NM_145018	220042	−1.24	−1.00	0.24	−0.25	S
A_24_P926543	*DDIAS*	AK058145	220042	0.41	0.29	−0.12	−0.14	S
A_23_P385126	*DEPDC7*	NM_139160	91614	0.99	0.06	−0.93	1.16	S
A_24_P320284	*DHFR*	NM_000791	1719	−1.51	−1.20	0.32	−0.38	S
A_24_P942328	*DHFR*	NM_000791	1719	−1.91	−1.52	0.39	−1.10	S
A_32_P211045	*DHFR*	NM_000791	1719	−2.16	−1.40	0.76	−1.27	S
A_23_P167553	*DHFR*	NM_000791	1719	−1.58	−1.13	0.45	−0.59	S
A_24_P343095	*DHFR*	NM_000791	1719	−1.26	−0.87	0.39	−0.36	S
A_23_P327361	*DMXL2*	NM_015263	23312	0.07	−0.14	−0.21	−0.02	S
A_24_P366107	*DNA2*	NM_001080449	1763	−0.86	−0.69	0.17	−0.56	S
A_23_P51339	*DNAJB4*	NM_007034	11080	1.16	−0.02	−1.18	0.98	S
A_24_P393958	*DNAJB4*	NM_007034	11080	1.11	−0.10	−1.21	1.23	S
A_23_P95359	*DNAJC6*	NM_014787	9829	−0.44	−0.40	0.03	−0.41	S
A_23_P147479	*DNAJC6*	NM_014787	9829	0.35	−0.02	−0.38	0.58	S
A_23_P500390	*DONSON*	NM_017613	29980	0.52	−0.11	−0.64	1.07	S
A_23_P425502	*DONSON*	NM_017613	29980	0.30	−0.34	−0.64	0.96	S
A_23_P35871	*E2F8*	NM_024680	79733	−0.76	−0.35	0.41	−0.23	S
A_23_P214291	*EFHC1*	NM_018100	114327	−0.37	−0.45	−0.08	0.72	S
A_32_P86245	*EFHC1*	NM_018100	114327	−0.31	−0.07	0.24	−0.10	S
A_24_P913374	*EIF4EBP2*	NM_004096	1979	−0.66	−0.29	0.36	−1.22	S
A_24_P4387	*EIF4EBP2*	NM_004096	1979	−0.42	−0.08	0.34	−0.58	S
A_24_P115621	*EIF4EBP2*	NM_004096	1979	−0.25	−0.04	0.21	−0.90	S
A_23_P115922	*EIF4EBP2*	NM_004096	1979	−0.22	0.01	0.24	−0.47	S
A_24_P323598	*ESCO2*	NM_001017420	157570	−2.00	−1.95	0.05	−0.79	S
A_23_P23303	*EXO1*	NM_003686	9156	−2.11	−1.68	0.42	−1.23	S
A_23_P259641	*EZH2*	NM_004456	2146	−0.74	−0.91	−0.16	0.13	S
A_23_P99853	*FAM214A*	NM_019600	56204	0.25	0.05	−0.20	−0.03	S
A_24_P357576	*FAM214A*	NM_019600	56204	0.17	0.09	−0.08	−0.27	S
A_23_P206441	*FANCA*	NM_000135	2175	−0.49	−0.42	0.07	−0.58	S
A_24_P73158	*FEN1*	NM_004111	2237	−1.16	−1.11	0.04	−0.36	S
A_24_P84898	*FEN1*	NM_004111	2237	−1.27	−1.29	−0.02	−0.33	S
A_23_P103996	*GCLM*	NM_002061	2730	0.55	1.42	0.87	−0.32	S
A_32_P177953	*GCLM*	ENST00000370238	2730	0.05	1.10	1.05	−0.67	S
A_32_P2392	*GOLGA8A*	NM_181077	23015	0.91	0.31	−0.60	0.66	S
A_23_P37623	*GOLGA8A*	NM_181077	23015	0.54	0.52	−0.02	−0.12	S
A_24_P910580	*GOLGA8A*	NR_027409	23015	0.50	0.47	−0.03	0.73	S
A_23_P29257	*H1F0*	NM_005318	3005	0.40	0.02	−0.39	−0.58	S
A_23_P349771	*HAUS5*	NM_015302	23354	0.41	0.89	0.48	−0.01	S
A_23_P12816	*HELLS*	NM_018063	3070	−0.95	−1.12	−0.17	−0.02	S
A_23_P372860	*HIST1H2AC*	NM_003512	8334	−0.38	−0.57	−0.19	0.76	S
A_23_P167983	*HIST1H2AC*	ENST00000314088	8334	−0.22	−0.24	−0.02	−0.03	S
A_32_P221799	*HIST1H2AM*	NM_003514	8336	−1.05	−1.41	−0.36	0.21	S
A_24_P86389	*HIST1H2AM*	NM_003514	8336	0.10	−0.07	−0.17	0.83	S
A_23_P93180	*HIST1H2BC*	NM_003526	8347	−0.04	−0.26	−0.22	0.90	S
A_24_P166407	*HIST1H4B*	NM_003544	8366	−0.91	−0.89	0.02	−0.16	S
A_23_P214487	*HIST1H4C*	NM_003542	8364	−1.27	−1.00	0.27	−0.66	S
A_23_P323685	*HIST1H4H*	NM_003543	8365	−0.31	−0.82	−0.51	0.73	S
A_23_P52266	*IFIT1*	NM_001548	3434	1.72	0.64	−1.08	1.80	S
A_23_P102454	*INSIG2*	NM_016133	51141	0.53	0.07	−0.46	1.13	S
A_24_P944458	*INSIG2*	NM_016133	51141	−0.30	−0.67	−0.37	0.31	S
A_23_P52082	*INTS7*	NM_015434	25896	−0.49	0.16	0.64	−0.44	S
A_32_P159651	*KAT2B*	NM_003884	8850	−0.66	−0.62	0.04	−0.51	S
A_23_P41128	*KAT2B*	NM_003884	8850	−0.38	−0.33	0.05	−0.36	S
A_23_P358542	*KIFC2*	NM_145754	90990	−0.21	−0.01	0.20	0.33	S
A_23_P165414	*KLHL23*	NM_144711	151230	−0.63	−0.12	0.51	−1.29	S
A_24_P923102	*KLHL23*	ENST00000392647	151230	−0.62	−0.02	0.60	−0.81	S
A_23_P165408	*KLHL23*	NM_144711	151230	−0.70	−0.36	0.34	−1.03	S
A_23_P74252	*LINC00339*	NR_023918	29092	−0.41	−0.62	−0.20	0.04	S
A_23_P84219	*LIPH*	NM_139248	200879	−0.15	−0.11	0.04	0.38	S
A_24_P799858	*LIPH*	ENST00000296252	200879	−0.03	0.14	0.17	−0.11	S
A_23_P380181	*LMO4*	NM_006769	8543	−0.36	−0.07	0.30	−0.34	S
A_32_P18159	*LYRM7*	NM_181705	90624	0.41	0.17	−0.23	−0.18	S
A_32_P211141	*LYRM7*	NM_181705	90624	0.26	0.44	0.18	0.07	S
A_24_P256603	*LYRM7*	NM_181705	90624	0.20	0.32	0.13	0.16	S
A_23_P337464	*LYRM7*	NM_181705	90624	−0.23	0.02	0.25	−0.66	S
A_24_P926195	*MAN1A2*	NM_006699	10905	−0.52	−0.01	0.51	−0.55	S
A_23_P103571	*MAN1A2*	NM_006699	10905	−0.48	0.14	0.62	−0.21	S
A_32_P88603	*MAN1A2*	ENST00000356554	10905	−0.49	0.09	0.59	−0.78	S
A_32_P88598	*MAN1A2*	ENST00000356554	10905	−0.42	0.25	0.66	−0.72	S
A_24_P213548	*MAN1A2*	NM_006699	10905	−0.11	0.41	0.52	0.02	S
A_23_P313640	*MAP3K2*	NM_006609	10746	0.60	0.45	−0.15	0.16	S
A_32_P98887	*MAP3K2*	ENST00000409947	10746	0.42	−0.11	−0.53	0.32	S
A_23_P313645	*MAP3K2*	NM_006609	10746	0.23	0.24	0.01	0.40	S
A_23_P201988	*MASTL*	NM_032844	84930	−0.45	−0.86	−0.41	0.36	S
A_24_P258051	*MASTL*	NM_032844	84930	−0.16	−0.38	−0.22	0.06	S
A_23_P92154	*MBD4*	NM_003925	8930	−0.37	−0.14	0.24	0.14	S
A_23_P68547	*MCM8*	NM_182802	84515	−0.81	−0.49	0.32	−0.43	S
A_24_P305556	*MCM8*	NM_182802	84515	−0.50	−0.48	0.03	−0.09	S
A_32_P129894	*MEGF9*	NM_001080497	1955	−0.12	0.13	0.25	−1.13	S
A_23_P426663	*MITF*	NM_198159	4286	−0.17	−0.15	0.02	−0.04	S
A_23_P73345	*MITF*	NM_198159	4286	0.26	0.19	−0.07	1.01	S
A_24_P910310	*MITF*	NM_198177	4286	0.14	−0.39	−0.53	0.72	S
A_23_P61945	*MITF*	NM_198159	4286	−0.03	0.11	0.13	−0.07	S
A_24_P323815	*MYCBP2*	NM_015057	23077	−0.48	−0.28	0.20	−0.08	S
A_23_P151459	*MYCBP2*	NM_015057	23077	0.04	0.56	0.52	−0.46	S
A_23_P209805	*NAB1*	NM_005966	4664	0.67	0.61	−0.06	1.30	S
A_24_P191417	*NAB1*	NM_005966	4664	0.01	0.29	0.28	0.47	S
A_23_P5761	*NFE2L2*	NM_006164	4780	0.04	0.26	0.22	−0.14	S
A_23_P23006	*NRDC*	NM_002525	4898	−0.42	−0.16	0.27	−0.07	S
A_32_P213822	*NSUN3*	ENST00000314622	63899	−0.66	−0.31	0.36	−0.19	S
A_23_P21785	*NSUN3*	NM_022072	63899	−0.08	−0.10	−0.01	0.55	S
A_23_P382043	*NT5DC1*	NM_152729	221294	−0.40	−0.15	0.25	−0.31	S
A_23_P219004	*NT5DC1*	NM_152729	221294	0.14	0.15	0.01	0.37	S
A_24_P922606	*NUP160*	NM_015231	23279	0.83	0.11	−0.72	0.83	S
A_23_P43726	*NUP160*	NM_015231	23279	−0.06	0.06	0.12	0.15	S
A_23_P381979	*OGT*	NM_181672	8473	−0.25	−0.44	−0.19	0.06	S
A_23_P381976	*OGT*	NM_181672	8473	−0.17	−0.08	0.09	0.12	S
A_23_P42045	*ORC3*	NM_181837	23595	−0.10	−0.60	−0.50	0.66	S
A_23_P79818	*OSER1*	NM_016470	51526	0.27	−0.36	−0.63	0.95	S
A_24_P261083	*OSGIN2*	NM_004337	734	0.89	0.41	−0.47	0.84	S
A_23_P82859	*OSGIN2*	NM_004337	734	−0.01	−0.06	−0.05	0.18	S
A_23_P117852	*PCLAF*	NM_014736	9768	−3.04	−2.31	0.73	−0.87	S
A_32_P61339	*PHIP*	BC036479	55023	−0.57	−1.00	−0.43	−0.08	S
A_24_P196400	*PHIP*	NM_017934	55023	−0.36	−0.34	0.01	0.15	S
A_23_P145437	*PHIP*	NM_017934	55023	−0.18	−0.09	0.09	0.20	S
A_24_P630039	*PHIP*	NM_017934	55023	−0.22	−0.03	0.20	−0.13	S
A_24_P931503	*PHIP*	NM_017934	55023	−0.21	−0.12	0.09	−0.61	S
A_24_P175176	*PHTF2*	NM_020432	57157	−0.76	−0.05	0.70	−0.58	S
A_32_P409919	*PHTF2*	NM_020432	57157	0.57	0.05	−0.52	0.50	S
A_24_P323944	*PHTF2*	NM_020432	57157	0.08	−0.05	−0.13	0.21	S
A_24_P403244	*PILRB*	NM_013440	29990	−0.31	0.08	0.38	0.15	S
A_23_P19829	*PILRB*	NM_013440	29990	−0.32	0.06	0.38	0.08	S
A_24_P105102	*PKMYT1*	NM_182687	9088	−1.77	−1.36	0.41	−1.22	S
A_23_P398515	*PKMYT1*	NM_004203	9088	0.12	0.19	0.07	−0.19	S
A_23_P25019	*PRIM1*	NM_000946	5557	−1.04	−1.21	−0.17	−0.32	S
A_23_P44139	*PRIM2*	NM_000947	5558	−0.53	−0.21	0.32	−0.16	S
A_24_P282237	*PRIM2*	NM_000947	5558	−0.33	−0.25	0.08	−0.17	S
A_24_P75158	*PTAR1*	NM_001099666	375743	−0.36	−0.37	0.00	0.26	S
A_23_P121222	*RAD18*	NM_020165	56852	−0.58	−0.47	0.11	−0.13	S
A_23_P88731	*RAD51*	NM_002875	5888	−1.73	−1.58	0.15	−1.02	S
A_23_P99292	*RAD51AP1*	NM_006479	10635	−2.35	−2.26	0.08	−1.01	S
A_23_P74115	*RAD54L*	NM_003579	8438	−2.14	−1.79	0.34	−0.94	S
A_23_P252371	*RBBP8*	NM_002894	5932	−0.40	−0.41	−0.01	0.30	S
A_23_P96285	*REEP1*	NM_022912	65055	−2.14	−1.68	0.45	−1.95	S
A_23_P93823	*RFC2*	NM_181471	5982	−0.45	−0.33	0.12	−0.47	S
A_23_P18196	*RFC4*	NM_002916	5984	−0.84	−0.88	−0.04	−0.04	S
A_23_P92710	*RHOBTB3*	NM_014899	22836	0.42	0.87	0.46	−0.96	S
A_23_P315386	*RHPN1*	NM_052924	114822	−0.19	−0.28	−0.09	0.50	S
A_23_P87432	*RHPN1*	NM_052924	114822	0.22	−0.57	−0.79	0.73	S
A_23_P87435	*RHPN1*	NM_052924	114822	−0.14	−0.25	−0.11	−0.38	S
A_23_P86133	*RPA2*	NM_002946	6118	−0.52	−0.77	−0.25	0.22	S
A_23_P87351	*RRM1*	NM_001033	6240	−0.73	−0.81	−0.08	0.31	S
A_24_P234196	*RRM2*	NM_001034	6241	−3.49	−2.56	0.93	−1.85	S
A_24_P225616	*RRM2*	NM_001034	6241	−1.98	−1.54	0.44	−1.50	S
A_24_P350160	*RSRC2*	NM_198261	65117	0.43	0.09	−0.34	1.05	S
A_23_P53267	*RSRC2*	NM_198261	65117	−0.16	−0.52	−0.36	0.70	S
A_24_P304987	*SAP30BP*	NM_013260	29115	−0.32	−0.05	0.27	−0.13	S
A_23_P54953	*SAP30BP*	NM_013260	29115	−0.23	−0.44	−0.21	0.26	S
A_23_P169351	*SH3GL2*	NM_003026	6456	0.07	−0.50	−0.57	−0.13	S
A_23_P200443	*SHC1*	NM_003029	6464	0.29	0.36	0.07	−0.42	S
A_24_P68585	*SHC1*	NM_183001	6464	0.06	0.14	0.08	−0.06	S
A_23_P202587	*SHTN1*	NM_018330	57698	−0.27	0.15	0.42	−0.46	S
A_32_P309404	*SLC22A3*	NM_021977	6581	−0.45	0.30	0.75	−0.81	S
A_23_P19733	*SLC22A3*	NM_021977	6581	0.20	0.72	0.52	0.12	S
A_24_P246841	*SLC25A27*	NM_004277	9481	0.40	0.38	−0.03	0.84	S
A_23_P81721	*SLC25A27*	NM_004277	9481	0.26	0.25	−0.01	0.57	S
A_23_P218079	*SLC38A2*	NM_018976	54407	0.24	0.33	0.09	1.24	S
A_24_P295963	*SLC38A2*	NM_018976	54407	−0.09	0.13	0.22	0.23	S
A_23_P104282	*SLF2*	NM_018121	55719	−0.37	−0.42	−0.05	−0.26	S
A_32_P143516	*SLF2*	NM_018121	55719	−0.14	−0.16	−0.02	0.17	S
A_23_P356139	*SLF2*	NM_018121	55719	−0.12	0.13	0.25	−0.62	S
A_23_P366312	*SP1*	NM_138473	6667	0.39	0.48	0.09	−0.14	S
A_23_P53397	*SP1*	NM_138473	6667	0.23	0.09	−0.15	−0.01	S
A_32_P45493	*SRSF10*	NM_006625	10772	−0.79	−0.49	0.30	0.03	S
A_23_P45737	*SRSF10*	NM_006625	10772	−0.43	−0.30	0.13	0.21	S
A_23_P352291	*SRSF10*	NM_054016	10772	−0.20	−0.18	0.01	−0.41	S
A_24_P4795	*SRSF10*	NM_054016	10772	−0.19	−0.09	0.10	0.57	S
A_23_P377819	*SRSF5*	NM_001039465	6430	−0.29	−0.21	0.08	−0.40	S
A_32_P45894	*STAG3L1*	NM_018991	54441	0.17	0.06	−0.12	0.53	S
A_24_P374962	*STAG3L1*	NM_018991	54441	0.05	0.20	0.15	0.21	S
A_24_P111242	*SVIP*	NM_148893	258010	−0.53	−0.55	−0.03	0.11	S
A_32_P41070	*TMCC1*	NM_015008	23023	−0.10	0.09	0.20	−0.29	S
A_32_P41065	*TMCC1*	NM_001017395	23023	−0.08	−0.15	−0.08	−0.17	S
A_24_P922288	*TMCC1*	NM_001017395	23023	0.08	−0.29	−0.37	0.60	S
A_23_P170986	*TMCC1*	NM_001017395	23023	−0.01	−0.12	−0.11	0.40	S
A_23_P39813	*TTC31*	NM_022492	64427	−0.34	0.04	0.38	−0.46	S
A_32_P204169	*TTLL7*	ENST00000260505	79739	0.29	0.86	0.57	−0.42	S
A_23_P97481	*TTLL7*	NM_024686	79739	−0.18	−0.38	−0.21	0.08	S
A_24_P165450	*TTLL7*	NM_024686	79739	0.02	−0.02	−0.03	0.15	S
A_23_P50096	*TYMS*	NM_001071	7298	−1.55	−1.70	−0.15	−0.34	S
A_23_P115482	*UBE2T*	NM_014176	29089	−1.18	−0.89	0.29	0.14	S
A_24_P330234	*UBL3*	NM_007106	5412	−0.30	−0.27	0.03	0.04	S
A_23_P140029	*UBL3*	NM_007106	5412	0.23	0.04	−0.19	0.04	S
A_23_P11652	*USP1*	NM_003368	7398	−0.44	−0.67	−0.23	0.22	S
A_23_P98483	*ZBED5*	NM_021211	58486	−0.41	−0.22	0.19	0.20	S
A_23_P210608	*ZNF217*	NM_006526	7764	0.02	−0.28	−0.30	0.05	S
A_23_P63789	*ZWINT*	NM_032997	11130	−2.88	−2.07	0.82	−1.15	S

a From Whitfield et al. (58).

b Phases: G1, G_1_; G2, G_2_; G2_M, G_2_/M; G2,G2_M, G_2_ or G_2_/M, etc.

### Intoxication with *Salmonella* supernatant containing CdtB induces DNA damage not coupled with cell cycle arrest.

**FIG 5:**
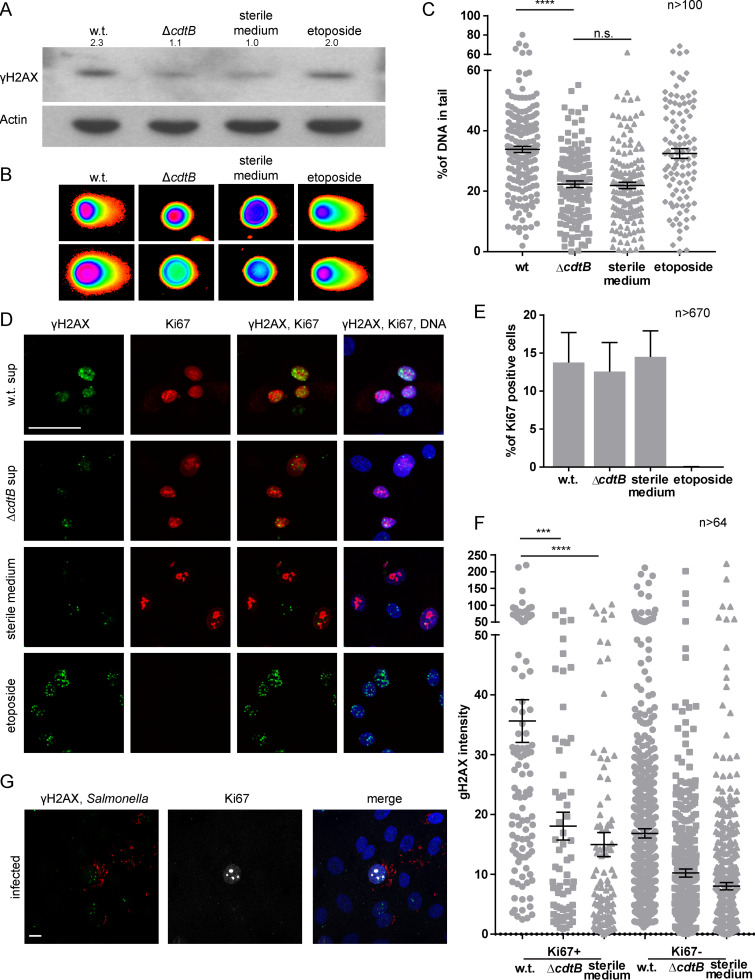
Intoxication of primary cell 2D monolayers with typhoid toxin-containing *Salmonella* supernatant. (A) Western blot analysis of γH2AX levels in primary cells after exposure to *Salmonella* supernatant or etoposide for 24 h. Relative densitometry values, normalized to the sterile medium condition (=1), are shown above the bands. (B) Comet assay showing that DNA damage, seen as a tail of DNA after electrophoresis, is higher after exposure to supernatant from w.t. *Salmonella* than from the *cdtB* deletion mutant. Etoposide served as a positive control. Pictures of two representative nuclei per condition are shown. (C) Quantification of the comet assay, shown as means ± the SEM. ****, *P* < 0.0001. (D) Immunofluorescence analysis of cells intoxicated or treated with etoposide for 24 h with antibodies against γH2AX (green) and Ki67 (red); nuclei were stained with Hoechst (blue). Scale bar, 25 μm. (E) Quantification of Ki67^+^ cells in intoxicated cells. Unlike cells treated with etoposide, cells intoxicated with w.t. *Salmonella* supernatant do not stop proliferation despite the presence of DNA DSBs. (F) Quantification of DNA damage in proliferating and non-proliferating cells. The intensity of the γH2AX signal was quantified for each Ki67 positive and negative nucleus, using ImageJ. Data shown as means ± the SEM. ***, *P* < 0.001; ****, *P* < 0.0001. (G) Primary cell monolayers infected for 3 days with *Salmonella* Paratyphi A transformed with the mCherry expressing vector pLS002 (red) and fluorescently labeled with antibodies against γH2AX (green) and Ki67 (white); nuclei were labeled with Hoechst (blue). Scale bar, 10 μm.

The majority of chronically infected carriers of typhoid *Salmonella* are usually diagnosed with gallstones and it has been found that *Salmonella* is able to grow on them forming biofilms. *Salmonella* covered gallstones might represent a reservoir for the bacteria but also a potential source of typhoid toxin (60). To understand the effect of the typhoid toxin on primary gallbladder cells, we sought to achieve a homogeneous typhoid toxin intoxication. To this aim, we seeded organoid-derived cells as 2D monolayers on collagen-coated plastic wells and supplemented them for 24 h with supernatant from *Salmonella* grown in MM5.8 medium, which is known to stimulate the production of typhoid toxin (19). Western blot analysis confirmed that only treatment with wild-type supernatant and etoposide, a chemical inducer of DSBs (61), resulted in an increased phosphorylation of H2AX, which is indicative of the presence of DSBs (Fig. 5A). The amount of DSBs was quantified using a neutral comet assay, which showed a significant increase in DNA in the tail of the comet analyzed from cells treated with supernatant from wild-type bacteria compared to supernatant from the Δ*cdtB Salmonella* or sterile medium (Fig. 5B, quantified in Fig. 5C). Cells treated with a genotoxic agent normally respond by arresting the cell cycle, and this was also reported for cells treated with recombinant CDT, a bacterial toxin that shares the CdtB subunit with the typhoid toxin (62). To examine whether cell cycle arrest was also induced in our intoxication model, we fluorescently labeled cells with antibodies against γH2AX and Ki67, a marker of proliferating cells. There was no difference in the percentage of Ki67^+^ cells in cultures treated with sterile, deletion mutant or wild-type supernatants (Fig. 5D, quantified in Fig. 5E). In stark contrast, cultures treated with etoposide contained little to no Ki67^+^ cells, although etoposide caused an amount of damage similar to that caused by the supernatant conditioned with wild-type typhoid toxin (Fig. 5A to C). Analysis of the γH2AX signal intensity in Ki67^+^ versus Ki67^–^ cells showed that supernatant conditioned with typhoid toxin induced more DNA damage, especially in proliferating cells (Fig. 5D and F). Cells intoxicated with *ΔcdtB* supernatant showed non-significant differences in the distribution of γH2AX intensities compared to sterile medium, both in proliferating and non-proliferating cells. The presence of cells positive for both γH2AX and Ki67 was detected only after intoxication with the wild-type typhoid toxin, and this observation occurred up to 48 h after intoxication started (see Fig. S3A and B). Finally, not only intoxicated cells but also rare infected cells were positive for γH2AX but still in an active state of proliferation, as indicated by double labeling with a Ki67 antibody (Fig. 5G). Our data show that human primary gallbladder cells are subjected to a low but persistent level of DNA damage caused by the CdtB subunit of the *S.* Paratyphi A-encoded typhoid toxin. The DNA damage caused by the genotoxin does not induce cell cycle arrest but particularly affects proliferating cells.

10.1128/mBio.01911-20.3 FIG S3 Long-term intoxication, 24 and 48 h. Human GB organoids were seeded in 2D and intoxicated for 24 or 48 h. For intoxication for 48 h, the bacterial supernatant was produced twice, and fresh supernatant was diluted in medium was added after 24 h. The cells seeded were less confluent than in normal 24-h intoxication experiments to avoid premature confluence of the culture. The figure shows double-positive cells for Ki67 and γH2AX at 24 h (A) and 48 h (B). *N*, the minimum number of counted cells per individual experiment. Download FIG S3, TIF file, 1.2 MB. Copyright © 2020 Sepe et al. 2020 Sepe et al. This content is distributed under the terms of the Creative Commons Attribution 4.0 International license.

## DISCUSSION

Here, we present a long-lived organoid model for human and murine GB. We found that long-term maintenance of GB organoid cultures depends on the presence of R-spondin, which mediates the activation of the Wnt/β-catenin signaling pathway and the regeneration of the gallbladder epithelium from Lgr5^+^ cells. These results confirm a recent report from a murine organoid model, which showed that the addition of R-spondin and Noggin, but not of Wnt ligands, was necessary for the expansion of GB stem cells *in vitro* (31). Since Wnt ligands are crucial for the activation of the Wnt/β-catenin pathway, we have found that the epithelium is itself the source of secreted WNT7A/7B. Our data suggest, in addition, that the organoids are able to transport organic ions, emulating the concentration of bile typical of this organ, and that GB epithelial features are stable over time in culture.

Resembling the architecture of the organ *in situ*, this organoid model provides an advanced platform for investigating GB pathology in primary, non-transformed cells. We therefore used GB organoids to develop a novel infection model for the human-specific, cancer-associated bacterium *S*. Paratyphi A, focusing on the genotoxic effect of the typhoid toxin. Previous data have suggested that bacterial internalization is essential for the secretion of the typhoid toxin by the host cell (17, 19–21). Here, we confirm that by infecting healthy human GB cells with a wild-type strain that produces the typhoid toxin, the non-infected bystander cells experienced genomic instability. This paracrine DNA damage effect depended on the presence of the CdtB subunit, moving attention to non-infected but intoxicated cells as potential targets of cellular transformation.

It has been shown that *Salmonella* is able to promote cell division through activation of the AKT and mitogen-activated protein kinase (MAPK) pathways. *S.* Typhimurium, which lacks the typhoid toxin, is able to induce tumor growth in a genetically predisposed primary mouse fibroblast model (63). We previously suggested that chronic carriers, subjected to low levels of genotoxicity and DSBs for years, might develop a similar genetic predisposition (64). Together with the anti-apoptotic effects of *Salmonella* on host cells (65, 66) and persistent inflammation, the enhanced damage is likely to contribute to an increased risk of developing malignant mutations observed in chronic carriers.

By using the organoids a source of cells to develop mucosoid cultures (as previously done for the human stomach) (34), we could generate an advanced model for *S*. Paratyphi A chronic infection *in vitro*. The gene expression profile of long-term *S*. Paratyphi A-infected mucosoid revealed that infection induced an initial cell cycle arrest that did not depend on the DNA damage caused by the typhoid toxin. It has been reported that bacteria use particular cell cycle phases for their invasion or replication. For these reasons they are equipped with factors known as cyclomodulins. *Salmonella* is known to preferentially invade mitotic cells (67) and is equipped with diverse cyclomodulins, including SpvB and PheA, that induce cell cycle arrest at different phases of the cell cycle depending on the type of infected cell (68). The typhoid toxin and other CdtB containing toxins, such as the cytolethal distending toxins (CDT), are also cyclomodulins since the DNA damage that they induce is known to induce cell cycle arrest, typically at the G_2_/M checkpoint (69–72). In the physiological settings of the mucosoids and using *S*. Paratyphi A, a wild-type typhoid toxin-producing strain, we could again detect DNA damage. However, we could not observe any stronger or longer effect due to the typhoid toxin over other effectors in blocking the cell cycle.

To distinguish the effect of the typhoid toxin over other bacterial effectors, we intoxicated primary epithelial cells derived from the organoids with a functional typhoid toxin obtained from bacterial supernatants. Our data confirm that the DNA damage is due to the action of the CdtB subunit of the typhoid toxin, but we showed in addition that damaged cells failed to arrest their cell cycle, and cells with higher levels of damage actually maintained proliferation. Chronic exposure to sublethal levels of recombinant CDT was previously found to induce genomic instability and anchorage-independent growth in Big Blue rat fibroblasts (25), suggesting that the duration of the exposure rather than the dose of the toxin is the key element that increases the risk of cellular transformation. In chronic carriers, healthy GB cells might get intoxicated with the typhoid toxin from neighboring infected *Salmonella* Paratyphi A cells or from gallstones coated with the same bacterium. The secretion of the toxin is at such a level that does not impair the cell cycle but still provokes DNA damage.

Future investigations should seek to understand whether the typhoid toxin leaves a genetic mutational signature in the gallbladder, as has been observed for other cancer types that have a signature reflecting the original mutagenic insult (15, 73, 74). Such a signature would provide an important molecular link between *Salmonella* and associated GBC.

## MATERIALS AND METHODS

### Human organoid culture.

Human gallbladder epithelial cells were derived from patients that underwent cholecystectomy (for details, see Table 5). The samples were stored in ice-cold phosphate-buffered saline (PBS) for up to 2 h, and then epithelial cell isolation was performed as described previously (75). Briefly, the tissue was washed with PBS from the residual bile and mucus, and it was then incubated at 37°C with the mucosal side facing a solution of 0.2% collagenase type IV. The mucosa was abraded thoroughly with the end of a glass microscope slide held at an angle of 45° every 5 min four times. The isolated cells were passed through a cell strainer with 70-μm pores and spun down, and 1 to 3 million cells were resuspended in a drop of 50 μl of Matrigel. The polymerized Matrigel drop was then supplemented with a medium based on Advanced/DMEM/F-12 (Invitrogen; described in Table 1). The medium was replaced twice a week. Every 7 to 10 days, the organoids were split at a ratio 1 to 3 or 4 by treatment with trypsin and then passed 10 times through a heat-narrowed Pasteur pipette. In the experiments in which single cells were seeded, trypsin-treated organoids were also passed through a 40-μm-pore cell strainer before seeding them in Matrigel.

**TABLE 5:** Patient information

ID	Age (yr)	Gender	Comments
GB6	66	F	Cholecystectomy due to presence of gallstones and inflammation.
GB10	61	F	Cholecystectomy due to presence of gallstones and inflammation.
GB11	56	M	Cholecystectomy due to presence of gallstones and inflammation.
GB12	37	M	Cholecystectomy due to polyps in the gallbladder; we got a healthy piece
GB13	66	M	Adenocarcinoma of the gastroesophageal junction, type III, otherwise healthy GB; patient received neoadjuvant chemotherapy before surgery and preventive cholecystectomy.
GB16	65	F	Gastric carcinoma, otherwise healthy GB; patient underwent gastrectomy and preventive cholecystectomy.

### Murine organoid culture.

Murine gallbladders were derived from mice with C57BL/6J genetic background. After sacrificing the mouse, the gallbladder was resected, cut in four pieces, and incubated in a thermal mixer at 37°C in 2 ml of TrypLE (Thermo Scientific) for 45 min. The tissue pieces were pipetted up and down five times to release the cells, big pieces were removed, and isolated cells were centrifuged, washed with Dulbecco modified Eagle medium (DMEM), and then seeded in 50-μl Matrigel drops. The polymerized Matrigel drop was then supplemented with the medium described in Table 1. The medium was replaced twice a week. The splitting procedure was the same as that described for the human organoids.

### Human gallbladder mucosoid culture.

The generation of the human gallbladder mucosoids follows a protocol that was previously published for the healthy human stomach (34). Briefly, single cells derived from organoid cultures were seeded on collagen-coated filters of Millicell standing cell culture inserts (Millipore, PIHP01250) at 150,000 cells/insert in primary cell medium (refer to Table 1 for more detail). Cells were incubated at 37°C, and the medium in the surrounding well was changed daily for the first 5 days, followed by twice a week. After 3 days, the medium on the filter was removed, and cells started to produce mucus that was withdrawn during medium change. Once a month, the mucosoids were split at a ratio of 1:3 by incubating the apical and basal sides of the mucosoids with trypsin-EDTA (0.5%). Single cells were reseeded again on new coated cell culture inserts.

### Lineage tracing.

For lineage tracing experiments, we used murine gallbladder organoids derived from C57BL/6J, Lgr5-EGFP-IRES-CreERT2, ROSA-mTmG^floxed^ mice. At 5 days after seeding, 10 μM 4-hydroxytamoxifen (4HT; Sigma) was added to the medium, and the mixture was kept for 2 days. The induction was performed only once.

### Microarray.

Organoids were harvested 4 or 14 days (small and big organoids, respectively) after seeding. The Matrigel drops containing the organoids were dissolved in 1 ml of TRIzol (Life Technologies), and RNA was isolated as described in the manufacturer’s protocol using glycogen as a coprecipitant. For mucosoids, filters were cut from the insert and dissolved thoroughly in 1 ml of TRIzol. Quality control and quantification of total RNA was assessed using a 2100 bioanalyzer (Agilent Technologies) and a NanoDrop 1000 UV-Vis spectrophotometer (Kisker).

**(i) Organoids.** Microarray experiments were performed as independent dual-color dye-reversal color-swap hybridizations using two biological replicates each. Total RNA was amplified and labeled with a dual-color Quick-Amp labeling kit (Agilent Technologies). In brief, mRNA was reverse transcribed and amplified using an oligo-dT-T7 promoter primer and labeled with cyanine 3-CTP or cyanine 5-CTP. After precipitation, purification, and quantification, 0.75 μg of each labeled cRNA was fragmented and hybridized to custom whole-genome human multipack microarrays (8 × 60k; Agilent, 048908) according to the supplier’s protocol (Agilent Technologies). Scanning of microarrays was performed at 3-μm resolution using a G2565CA high-resolution laser microarray scanner (Agilent Technologies). Microarray image data were processed with Image Analysis/Feature Extraction software G2567AA vA.11.5.1.1 (Agilent Technologies) using default settings and the GE2_1105_Oct12 extraction protocol. The extracted dual-color raw data .txt files were further analyzed using R and the associated BioConductor package limma (76). Microarray data have been deposited in the Gene Expression Omnibus (GEO; www.ncbi.nlm.nih.gov/geo/) of the National Center for Biotechnology Information and can be assessed with the GEO accession number GSE100656.

For GSEA, a gene set of β-catenin target genes published previously (44) and human pluripotent stem cell genes published by Mallon et al. (42) (Tables 2 and 3) were used, and GSEA was performed on genes preranked by gene expression-based *t* score between early and differentiated organoids, using the fgsea R package (77) with 5,000 permutations. Wnt family member’s average intensities were calculated by global average in all the conditions. They were then filtered for an average intensity of >6. The differentially expressed ones were then identified as having a *P* value of <0.05.

**(ii) Mucosoids.** Single-color hybridizations using two technical replicates each were conducted. Microarrays used had design Agilent-014850 whole human genome microarray 4x44K G4112F (Agilent Technologies) and were read using the machines and software of the same manufacturer. The extracted raw data .txt files were further analyzed using R and the associated BioConductor package limma (76). Since MSigDB gene sets use human gene symbols to map genes to pathways, mouse symbols were translated to homologous human symbols using HomologeneDB from NCBI. GSEA was also performed on gene sets for cell cycle associated genes (58) from MSigDB v7.0 (PMID 21546393) (Table 4) between wild-type (w.t.) and Δ*cdtB* strain-infected mucosoid versus non-infected at 2 and 7 days post infection.

For human gene sets (i.e., MSigDB and those derived from human experiments), the full set of genes in the DGE results after collapsing *t* scores by gene and ranking was used. To analyze the mouse gene sets, the DGE data were restricted to probe sets that have a homologous gene in mice and humans. For these probe sets, the one with the highest *t* score and rank in the resulting list was selected and subsequently used for fGSEA analysis.

Expression data were analyzed as follows. For each of the selected comparisons, the replicates of the target condition were compared to the corresponding control using limma, producing differential expression statistics for all genes and comparisons. Analyses were performed as individual two-group unpaired comparisons: 2-day infection, w.t. versus NI; 2-day infection, Δ*cdtB* versus NI; 2-day infection, Δ*cdtB* versus w.t.; 7-day infection, w.t. versus NI; 7-day infection, Δ*cdtB* versus NI; and 7-day infection, Δ*cdtB* versus w.t.

The interpreting plotting of the results was done using Microsoft Excel, and the software R/R Studio was used to create the plots for the heatmaps. The heatmaps were plotted by using the normalized expression values (log-normalized intensity) again normalized on the non-infected control of each time point (logFC) when expression data from single genes were plotted and the calculated NES scores, respectively, for pathway analysis.

### Immunofluorescence.

Organoids were removed from Matrigel at the indicated time point by washing with ice-cold PBS and then fixed with 3.7% paraformaldehyde. Tissue pieces were washed with PBS and fixed. After fixation, organoids and tissue pieces were embedded in paraffin and cut with a microtome to get 5-μm slices. Cells seeded in 2D (two dimensions) were washed with PBS and fixed. For whole-mount staining, the organoids were fixed directly in the Matrigel drop and then stained. The staining was performed with the antibodies and dyes listed in Table 6. Images were acquired with a Leica TCS SP-8 confocal microscope. For immunofluorescence of the mucosoids, the filters were cut form the insert, and pieces of the filters were blocked in a bovine serum albumin-containing blocking buffer for 3 h for whole-mount staining. Alternatively, the filters were fixed overnight in 4% paraformaldehyde (PFA) at 4°C, washed, embedded orthogonally in Histogel (HG-4000-144) inside a casting mold, and paraffinized overnight in a Leica TP1020 tissue processor. The paraffin blocks were generated inside a casting mold on a Paraffin console (Microm). Next, 5-μm sections were cut with a paraffin rotation microtome (Microm). For dewaxing and antigen retrieval, sample slides were washed twice with xylene (10 min), followed by a descending series of alcohols (20 s each), followed by two washes with water and 30 min in target retrieval solution (Dako) at 95°C and 20 min at room temperature and 5 min under running water. Primary antibodies were diluted in the blocking solution: in-house-made anti-γH2AX conjugated to ATTO488 (a green fluorescent dye; 1:500), phalloidin-Alexa 647 (lot 1731699; 1:100), and Hoechst (1:1,000; Sigma, B2261, lot 019K4029). Antibodies were incubated overnight at room temperature in the dark. Next, filter pieces were washed three times with blocking solution for 3 h at room temperature in the dark. The stained filters were mounted in Vectashield (Vector Laboratories, H-1500) on a glass slide, and the images were acquired using an SP-8 confocal microscope. The pictures are a result of a projection of multiple z-stacks analyzed with the software ImageJ.

**TABLE 6:** Antibodies and dyes

Antibody	Supplier	Catalog no.	Source	Application(s) (dilution)^*a*^
Anti-β-Actin (AC-15)	Sigma	A5441	Mouse monoclonal	WB (1:10,000)
E-cadherin (clone CD324)	BD	562869	Mouse monoclonal	IF (1:300), WB (1:500)
Ki67 (D2H10)	Cell Signaling	9027	Rabbit monoclonal	IF (1:200)
PCNA (csPC10)	Cell Signaling	2586	Mouse monoclonal	IF (1:100)
Phospho-histone H2A.X (Ser139) (clone JBW301)	Millipore	05-636-I	Mouse monoclonal	WB (1:200)
Phospho-histone H2A.X (Ser139)-conjugated FITCS	In house		Mouse monoclonal	IF (1:500)
β-Catenin	Sigma	C2206	Rabbit polyclonal	IF (1:300), WB (1:500)
Claudin-2	Abcam	ab53032	Rabbit polyclonal	IF (1:200), WB (1:500)
Cytokeratin 19 (EP1580Y)	Abcam	ab52625	Rabbit monoclonal	IF: 1:500), WB (1:5,000)
Muc5B	Abcam	ab87376	Rabbit polyclonal	IF (1:300)
Vimentin (D21H3)	Cell Signaling	5741	Rabbit monoclonal	WB (1:1,000)
Hoechst (bisbenzimide H 33258)	Sigma	H6024		IF (1:1,000)
Draq5	Abcam	ab108410		IF (1:1,000)
Phalloidin 647	Invitrogen	A22287		IF (1:500)

a WB, Western blotting; IF, immunofluorescence.

### Transmission electron microscopy.

Infected and non-infected gallbladder mucosoids were washed in-well with PBS, fixed with 4% PFA for 30 min, and washed twice with PBS. Filters were cut from the insert and cropped into pieces with bacterial patches under visual control. Cropped filter pieces were stored in PBS at 4°C until use. For fine structural analysis, cell layers on filters were fixed with 2.5% glutaraldehyde, postfixed with 0.5% osmium tetroxide, contrasted with uranyl acetate and tannic acid, dehydrated in a graded ethanol series, and infiltrated in Polybed (Polysciences). Cut-out pieces of the filters were stacked in flat embedding molds with Polybed. After polymerization, specimens were cut at 60 nm and contrasted with lead citrate. Specimens were analyzed in a Leo 906E transmission electron microscope (Zeiss, Oberkochen, Germany) equipped with a side-mounted digital camera (Morada, SIS-Olympus, Münster, Germany). Figures were assembled with the help of a FigureJ-Plugin (78).

### Western blotting.

For the Western blots, organoids and cells seeded in 2D were harvested in Laemmli buffer, and 12% SDS-PAGE gels were run and transferred to a nitrocellulose membrane, which was then blotted with the antibodies listed in Table 6. Densitometry was calculated using ImageJ software.

### Functional assay.

The functional assay is a modified version of a previously described assay (54). Briefly, 1 week after seeding, the organoids were incubated with DMEM/F-12 (Invitrogen) containing 100 μM rhodamine-123 (Sigma) for 5 min, washed with three times with PBS, and supplemented with the regular medium. Images were taken every minute with a Leica SP-E confocal microscope for 30 min. Temperature and CO_2_ concentration were kept at 37°C and 5%, respectively. To show that transport of rhodamine-123 depends on activity of multidrug-resistant (MDR) gene products, the organoids were incubated with 10 μM verapamil (Sigma), an MDR inhibitor, for 30 min before rhodamine-123 was added. As a negative control, gastric organoids were used, cultivated as previously described (28).

### Bacterial strains.

Salmonella enterica serovar Paratyphi A (ATCC 9150) was used for the infection experiments. An isogenic mutant knockout of *cdtB* was generated by interrupting the gene with a kanamycin resistance cassette. Briefly, two sequences were amplified upstream and downstream *cdtB* using the primers TCTATAGTTGTCTCTTTGGTATTAAC and CGCGGATCCACCATAAGAATATCC for the region upstream and the primers CGCGGATCCATATAAGATATATCT and ACAGCTTCGTGCCAAAAAGG for the region downstream. After insertion in a pGEM-T Easy vector (Promega), a kanamycin resistance cassette was inserted in between by making use of the BamHI sites included in the primers. The resulting region was PCR amplified and electroporated in *Salmonella*, and the clones where homologous recombination occurred were selected, as described previously (79). If mentioned, to visualize the bacteria, the w.t. and Δ*cdtB* strains were additionally transformed with pLS002, a plasmid carrying the constitutively expressed mCherry gene and an ampicillin resistance cassette.

### Infection experiments.

Organoids were removed from Matrigel by washing with ice-cold PBS, mechanically sheared by pipetting them three times through a heat-narrowed Pasteur pipette, and incubated at 37°C with 300 μl of primary medium containing log-phase *Salmonella* to a multiplicity of infection of 100 for 2 h. The cells were pelleted and washed twice with PBS before reseeding them in Matrigel. The gentamicin protection assay was performed by incubation for 1 h in primary medium supplemented with 100 μg/ml gentamicin. At this point, the invasion assay was performed. The organoids were removed from Matrigel and washed twice with PBS, the membrane was permeabilized by 2 min of incubation with 1% Triton X-100, and then sequential dilutions were plated on LB agar plates. The following day, colonies were counted as follows: invasion percentage = (CFU recovered from the infected organoids/bacteria used for infection) × 100. In the well with the remaining infected organoids, the concentration of gentamicin was decreased to 10 μg/ml for the duration of the experiment (80). Infection of mucosoids with *Salmonella* was done accordingly: log-phase mCherry-transformed *Salmonella* was administered on the filter to a multiplicity of infection of 100 for 24 h by using a penicillin-streptomycin-free 3D gallbladder medium (see Table 1). The infection medium was then removed, the filters and wells were washed with 37°C PBS, and the gentamicin protection assay was performed by incubation for 1 h in primary medium supplemented with 100 μg/ml gentamicin. The gentamicin concentration was then reduced to 10 μg/ml and withdrawn completely at 48 h post infection. The cells were washed, and the medium was refreshed every 2 days.

### Intoxication experiments.

Organoids were split to single cells, seeded onto a type I collagen (Thermo Fisher, A10644-0)-coated plastic (10 μg/cm^2^) or glass (15 μg/cm^2^) surface, supplemented with the conventional 3D medium, and intoxicated when 50% confluence was reached. The typhoid toxin-containing *Salmonella* supernatant was prepared by using a modified version of a previously described protocol (19). Briefly, the bacteria were grown in Luria-Bertani overnight, diluted 1:50 in MM5.8 (19, 81), and then grown overnight until the optical density at 600 nm reached 0.4 to 0.5. The bacteria were then removed by centrifugation and subsequent filtration through 0.4-μm-pore filters. The supernatant was then concentrated 20-fold using an Amicon Ultra-15 column. It was then diluted 1 to 20 in primary medium and incubated for 24 h with the cells. As a positive control, the cells received 50 μM etoposide (Sigma) for 24 h.

### Neutral comet assay.

The neutral comet assay was performed after intoxication using the kit from Trevigen according to the manufacturer´s protocol. Images were acquired using fluorescence microscope (Leica DMR). The percentage of DNA in the tail (which is a measure of DNA damage) was quantified using Comet Score software (TriTek).

### Data availability.

Microarray data have been deposited in the Gene Expression Omnibus (GEO; www.ncbi.nlm.nih.gov/geo/) of the National Center for Biotechnology Information and can be accessed under GEO accession number GSE100656.
