# Supplementary figures and images for: Genotoxic Effect of Salmonella Paratyphi A Infection on Human Primary Gallbladder Cells
Source: mBio. 2020 Sep 22;11(5):e01911-20. doi: 10.1128/mBio.01911-20 (PMC7512552; doi:10.1128/mBio.01911-20)

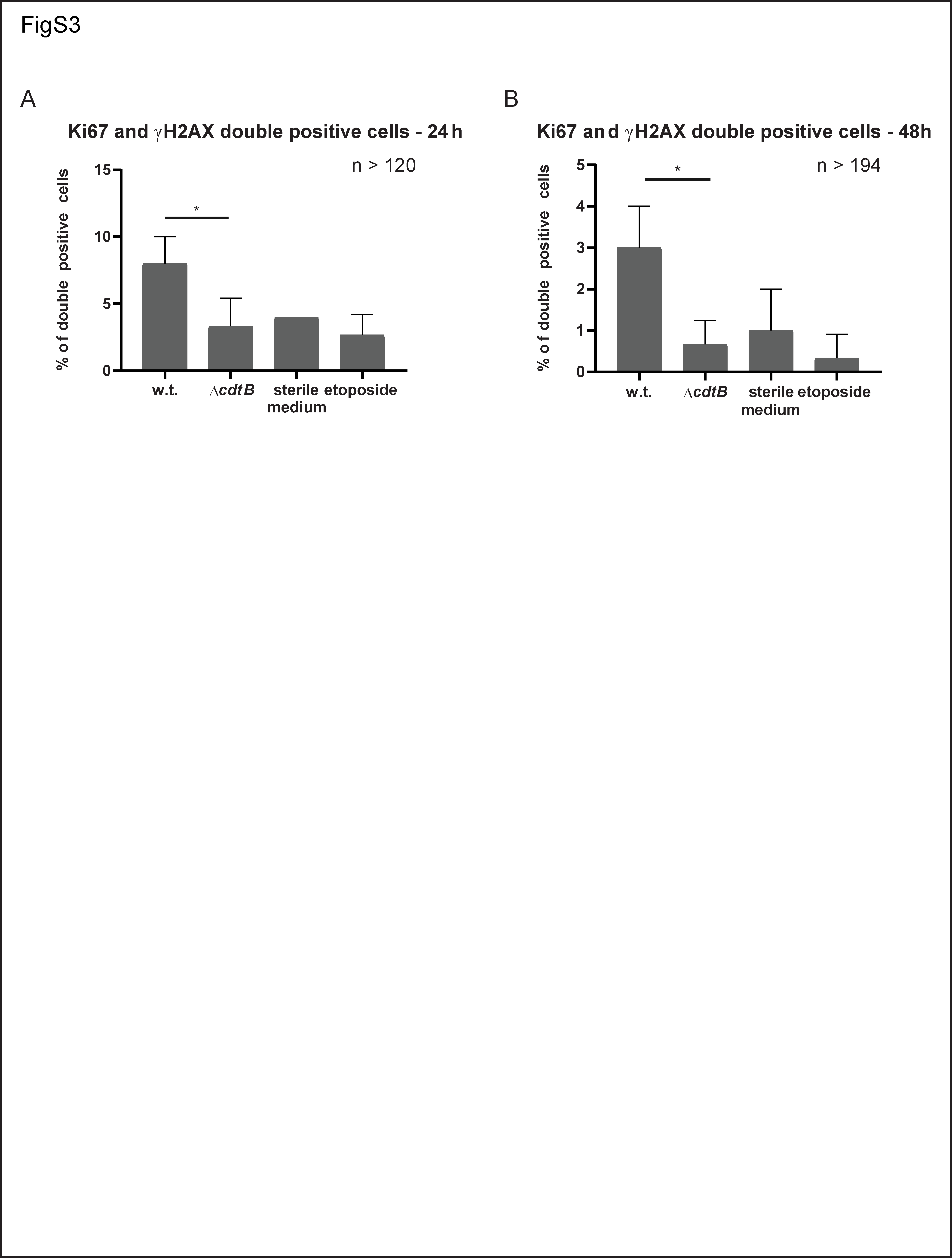

Supplement: FIG S3 [file mBio.01911-20-sf003.tif]
